# Disentangling demographic effects of red deer on chamois population dynamics

**DOI:** 10.1002/ece3.7657

**Published:** 2021-05-25

**Authors:** Valerio Donini, Luca Pedrotti, Francesco Ferretti, Luca Corlatti

**Affiliations:** ^1^ Stelvio National Park, Sustainable Development and Protected Areas Service Autonomous Province of Trento Cogolo di Pejo Italy; ^2^ Stelvio National Park Bormio Italy; ^3^ Department of Life Sciences University of Siena Siena Italy; ^4^ Chair of Wildlife Ecology and Management University of Freiburg Freiburg Germany

**Keywords:** climate, density dependence, interspecific competition, life‐history traits, population dynamics, ungulates

## Abstract

Investigating the impact of ecological factors on sex‐ and age‐specific vital rates is essential to understand animal population dynamics and detect the potential for interactions between sympatric species. We used block count data and autoregressive linear models to investigate variation in birth rate, kid survival, female survival, and male survival in a population of Alpine chamois *Rupicapra rupicapra rupicapra* monitored over 27 years within the Stelvio National Park, Central Italian Alps, as function of climatic variables, density dependence, and interspecific competition with red deer *Cervus elaphus*. We also used path analysis to assess the indirect effect of deer abundance on chamois growth rate mediated by each demographic parameter. Based on previous findings, we predicted that birth rate at [*t*] would negatively relate to red deer abundance at year [*t* − 1]; survival rates between [*t*] and [*t* + 1] would negatively relate to red deer abundance at year [*t* − 1] and to the interactive effect of winter precipitation at [*t* + 1] and chamois density at [*t*]. Our results showed that birth rate was positively related to spring–summer precipitation in the previous year, but this effect was hampered by increasing red deer abundance. Kid and female survival rates were negatively related to the combined effect of chamois abundance and winter precipitation. Male and female survival rates were negatively related to lagged red deer abundance. The path analysis supported a negative indirect effect of red deer abundance on chamois growth rate mediated by birth rate and female survival. Our results suggest that chamois population dynamics was largely explained by the synergistic effect of density dependence and winter harshness, as well as by interspecific competition with red deer, whose effects were seemingly stronger on the kid–female segment of the population.

## Graphical abstract

Understanding variation in key life‐history traits requires to disentangle the impact of ecological variables on sex‐ and age‐specific vital rates. Red deer *Cervus elaphus* can act as a potential competitor to the chamois *Rupicapra rupicapra*, hampering population growth rate. Our results support a strong negative effect of red deer abundance on chamois demographic parameters, thereby supporting the role of interspecific competition, especially on the kid–female segment of the population.

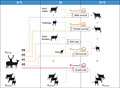

## 1 INTRODUCTION

Investigating the dynamics of animal populations is essential to understand ecological processes and to optimize decision‐making in wildlife management and conservation (Gaillard et al., 1998; Lande et al., 2003). The numerical changes in wildlife populations over time may depend on endogenous factors such as density‐dependent food limitation or demographic structure (Dennis & Otten, 2000; Coulson et al., 2001), as well as on exogenous variables such as environmental conditions, predation, interspecific competition, and hunting (Amundsen et al., 2007; Clutton‐Brock & Albon, 1982; Putman, 1996). These factors may impact several phenotypic components, such as morphological, behavioral, physiological, and life‐history traits (cf. Coltman et al., 2003; Isaac, 2009; Leclerc et al., 2017).

Several studies have investigated how ecological factors affect overall population growth rate (see, e.g., Dennis & Taper, 1994; Mysterud et al., 2002). Understanding variation in key life‐history traits, however, requires to disentangle the impact of ecological variables on sex‐ and age‐specific vital rates such as survival and reproduction (Bellier et al., 2018; Gaillard et al., 1998). In large herbivores living in temperate climates, population size variations mostly depend on density‐ and climate‐related food limitation (Clutton‐Brock et al., 1987; Gaillard et al., 2000; Putman et al., 1996). Increasing densities foster intraspecific competition, with consequent reduction in population growth rate, owing to changes in different vital rates such as juvenile survival (Bonenfant et al., 2009; Coulson et al., 2001; Pettorelli et al., 2007; Sæther, 1997), age at first reproduction and fecundity (Fowler, 1987; Gaillard et al., 1998). Climate severity also affects population growth rate through reproduction and survival. Winter harshness, for example, may increase juvenile mortality and reduce birth rate (Guinness et al., 1978), often acting in synergy with density dependence (Bonardi et al., 2017; Clutton‐Brock et al., 1985; Putman et al., 1996; Weladji et al., 2002). Spring–summer precipitation and temperature may also alter plant composition (Sæther, 1985) and influence nutritional quality of food resources, consequently impacting female forage consumption and kid survival in the first months of life (Albon et al., 1987; Sæther & Haagenrud, 1983). Furthermore, interspecific competition between large herbivores may lead to changes in resource use, behavior, and, ultimately, demography (Clutton‐Brock & Coulson, 2002; Ferretti et al., 2011; Forsyth & Hickling, 1998; Putman, 1996). For example, in female ungulates quality and abundance of forage are key elements to ensure growth and kid survival during winter (Côté & Festa‐Bianchet, 2001), and the presence of a competitor may alter resource availability, thereby leading to increased kid mortality and lower reproductive success (Ferretti et al., 2015).

The chamois *Rupicapra* spp. is the most abundant mountain ungulate in Europe (Corlatti et al., 2011). Although the chamois as a genus is not at risk, some populations have recently shown a decreasing trend, and some subspecies are threatened (Anderwald et al., 2021). Understanding the drivers of chamois population dynamics is thus important to the management and conservation of the species. Some general consensus exists about the dependence of Northern chamois *Rupicapra rupicapra* population growth rate on winter weather conditions and density, albeit with differences among study areas (cf. Capurro et al., 1997; Willisch et al., 2013; Ciach & Pęksa, 2018). Information on the drivers of chamois population demographic components, however, is scantier and somewhat contradictory. Kid/female ratio (birth rate) at year [*t*] was found either to be density‐independent (Capurro et al., 1997) or to depend on female population density in the same year (Willisch et al., 2013), but not on climatic variables (Capurro et al., 1997; Willisch et al., 2013). Kid survival between [*t*] and [*t* + 1] was found to be hampered by total population density with a 2‐year time lag (Capurro et al., 1997) or by the severity of winter conditions at [*t* + 1] or [*t*] (Willisch et al., 2013; but see Capurro et al., 1997), while adult survival between [*t*] and [*t* + 1] was found to either depend on density at time [*t* − 1] (Willisch et al., 2013) or [*t* − 2] (Capurro et al., 1997). Recently, studies on Apennine chamois *Rupicapra pyrenaica ornata* supported negative effects of competition with red deer *Cervus elaphus* and of high temperature/low rainfall during the vegetative season on survival of chamois kids between [*t*] and [*t* + 1] (Ferretti et al., 2015, 2019). More generally, studies on the demographic effects of competition are rare, and no information about the impact of interspecific competition on vital rates exists for Alpine chamois *R.r. rupicapra*.

**FIGURE 1:**
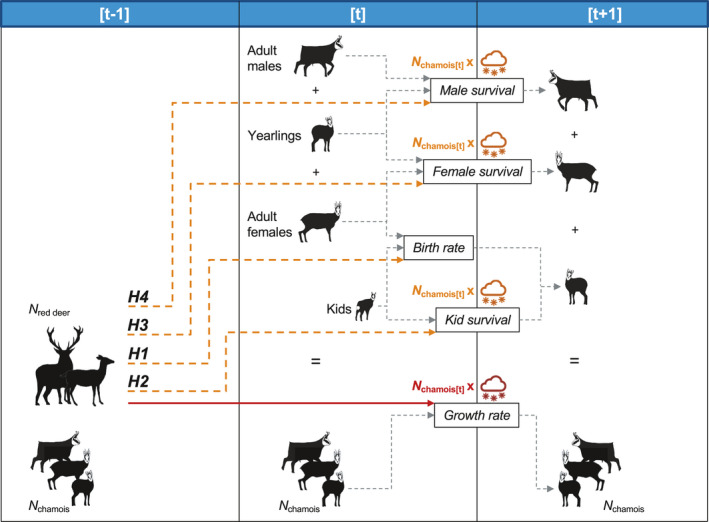
Scheme of the patterns hypothesized in this study to explain variation in demographic parameters of Alpine chamois in the Stelvio National Park. Demographic parameters are reported within rectangles (see text for details), and gray dashed arrows indicate the contribution of each sex and age classes. Different hypotheses to explain variation in chamois birth rate (*H1*) and survival rates (*H2*–*H4*) are represented with orange dashed lines, symbols, and letters. Dashed lines indicate the hypothesized negative effects of red deer abundance, while symbols and letters indicate the hypothesized negative effects of winter weather conditions at [*t* + 1] in synergy with chamois abundance at time [*t*]. Red solid line and letters indicate the known negative relationship of red deer at [*t* − 1] and of the interactive effects between chamois abundance at [*t*] and winter weather conditions at [*t* + 1] with chamois population growth rate between [*t*] and [*t* + 1]. Drawings by Luca Corlatti

In a recent study, Corlatti et al. (2019) investigated the long‐term dynamics of a chamois population in the Stelvio National Park (Central Italian Alps) and found that chamois growth rate between year [*t*] and [*t* + 1] was negatively related to red deer abundance at year [*t* − 1], as well as to the combination of winter precipitation at [*t* + 1] and chamois abundance at [*t*]. These biotic and abiotic drivers are expected to impact vital rates directly, eventually influencing population growth. In Corlatti et al. (2019), chamois growth rate was calculated excluding kids; therefore, it can be affected by birth rate at year [*t*], kid survival between year [*t*] and [*t* + 1] (thereby allowing for yearling recruitment), and adult female and male survival between year [*t*] and [*t* + 1]. In this study, we aim to assess the direct role of climatic variables, density dependence, and red deer abundance on the variation of key demographic parameters in the same chamois population. In principle, red deer abundance at [*t* − 1] may affect both survival and birth rate, while the synergistic effect of winter precipitation at [*t* + 1] and chamois abundance at [*t*] could affect survival, but not birth rate. Therefore, we put forward the nonmutually exclusive hypotheses that red deer abundance at [*t* − 1] negatively relates to chamois birth rate at time [*t*] (*H1*); red deer abundance at [*t* − 1] and the interaction between winter precipitation at [*t* + 1] and chamois abundance at [*t*] negatively relate to kid survival (*H2*), adult female survival (*H3*), and adult male survival (*H4*) between time [*t*] and [*t* + 1]. The hypothesized relationships are shown in Figure 1. Given the importance of interspecific competition in this population, we also aim to verify the occurrence of an indirect negative effect of red deer abundance on chamois growth, and test whether this effect is mediated by birth rate, kid survival, adult female survival, or adult male survival (*H5*).

## 2 MATERIALS AND METHODS

### 2.1 Study area

Our study area was located in the Trentino sector of Stelvio National Park (Central Italian Alps) and covers about 17,520 hectares with altitudes ranging from 1,500 to *c*. 3,900 meters a.s.l. (10.69567 E, 46.41486 N; see Figure 2 in Corlatti et al., 2019). The climate is alpine, with mean temperatures of about −0.4°C in winter and about 13.2°C in summer; yearly mean precipitation is 900 mm (Bonardi et al., 2017). The lower part of the study area is covered by coniferous forests of spruce *Picea abies*, larch *Larix decidua*, and Swiss pine *Pinus cembra*, while above the treeline (>2,000 m. a.s.l.) alpine and subalpine meadows include patches of Alpine sedge *Carex curvula*, Haller's fescue *Festuca halleri*, and colored fescue *Festuca varia*. Red deer was declared extinct in the area in the first half of the 19th century; following a steady increase from the early 1980s, the red deer population reached peaks of some 2,000 individuals in 2008 and the current density is about 8 individuals/km^2^, with winter density of about 20/km^2^ (Bonardi et al., 2017). Over the same time span, chamois population size has decreased considerably (cf. Corlatti et al., 2019). Hunting is not allowed within the study area, and the presence of predators is still rare. Furthermore, no severe chamois disease outbreaks have occurred during the study period.

**FIGURE 2:**
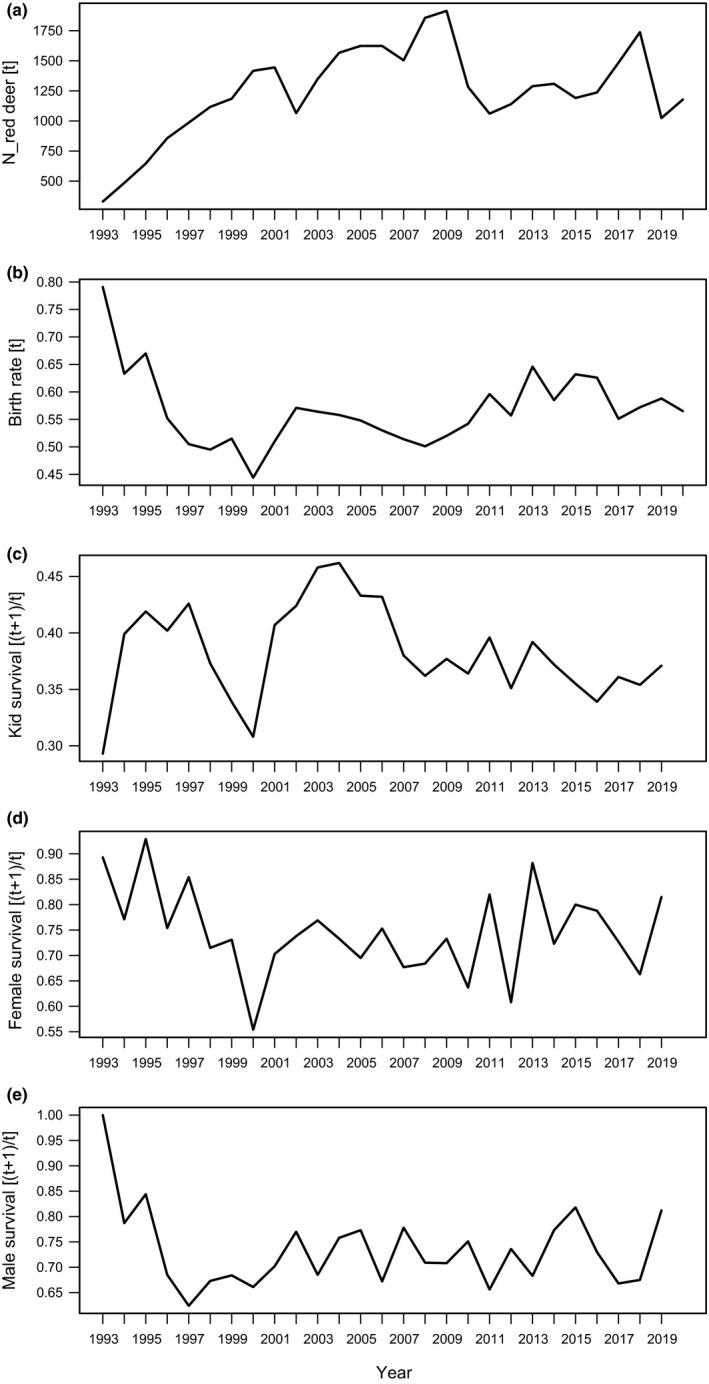
Temporal trends of red deer abundance (panel a) and chamois demographic parameters within the Stelvio National Park between 1993 and 2020: birth rate at time [*t*] (panel b); kid survival between [*t*] and [*t* + 1] (panel c); female survival between [*t*] and [*t* + 1] (panel d); and male survival between [*t*] and [*t* + 1] (panel e)

### 2.2 Data collection

Chamois demographic parameters were investigated over a 27‐year time span, from 1993 to 2020, and they were defined using block count data. Block count is arguably the most widely used technique to monitor chamois populations in high altitude open areas (cf. Loison et al., 2006; Corlatti et al., 2015) and consists of counting animals from fixed vantage points or along trails using optical instruments (Corlatti et al., 2015). The chamois survey area covered about 13,400 hectares, subdivided into smaller “blocks,” that is, sectors (*n* = 42) of *c*. 362 ha (*SD* = 179), chosen on the basis of natural boundaries such as valleys, ridges, and rivers, to favor animal detectability. Each year, counts were conducted by experienced park personnel in late July, from 06.00 to 09.00 a.m.; at least two rangers per sector were present. During counts, rangers noted down, for each group of chamois, the number of animals, their sex, and age class. Specifically, chamois were classified as kids (individuals born in the spring of the current year), yearlings (1 year of age), adult females (2+ years of age), and adult males (2+ years of age) based on physical characteristics (cf. Corlatti et al., in press). Due to chamois limited sexual dimorphism, identification of sex or age classes can at times be problematic. When uncertain, rangers usually classified animals as “unknown.” Animals falling into the age categories “unknown 2+ years” (about 14.6% of the entire dataset) or “unknown 1+ years” (7.5%) were added to the final dataset by assigning individuals to different sex and age classes based on the respective ratio of the available data; as the number of unknown individuals may change from year to year, this reassignment was necessary to avoid bias in the calculation of demographic parameters (cf. Capurro et al., 1997). During observations, rangers noted down on a map the time and position of animal groups, and they used radio transmitters to communicate chamois movements and avoid double counts. By the end of each survey, individuals observed in different sectors were summed, to obtain sex‐ and age‐specific count data pooled across the entire study site, within each year. Pooling was necessary to avoid bias in the calculation of demographic parameters due to animal movements within the study site.

To investigate the relationships between red deed abundance and chamois demographic parameters, red deer numbers were obtained on a yearly basis using spring spotlight counts, which proved effective to track changes in deer population size in Alpine environments (Corlatti et al., 2016). Each year in April–May, park rangers simultaneously drove along predefined routes in the lower part of the study area, between 11.00 p.m. and 03.00 a.m., and noted down all sighted animals. At least three surveys were conducted, and the one with the greatest number of animals counted was used for analysis. Raw counts were adjusted for an underestimate of 0.35 and filtered using state‐space models and a Kalman filter approach (see details in Bonardi et al., 2017, and next paragraph). It should be noted that for both species, counts were purposely conducted when detection probabilities of individuals are greatest, hence minimizing counting biases.

As demographic parameters were calculated pooling count data across different observation sectors within each year, to assess the relationship between climatic variables and chamois demographic parameters we had no need to account for between‐sector variation in meteorological conditions (cf. Jacobson et al., 2004). Historical climatic data were thus collected from two meteorological stations (one for snow, at 2,010 m a.s.l., one for precipitation at 1,580 m a.s.l.) within the study area (available from: www.meteotrentino.it). The set of climatic variables included only summed precipitation (in mm) in winter (January–March: *P*_winter_) and in spring–summer (April–July: *P*_spring‐summer_), as these variables proved to have the highest explanatory power in a previous study on the same population (Corlatti et al., 2019). Preliminary explorations of data supported the use of these variables over other potential climatic variables such as average daily temperature in winter or spring–summer, and mean snow depth in winter (the latter variable was nonetheless positively and significantly correlated with winter precipitation).

### 2.3 Demographic parameters

Different demographic parameters were defined to investigate *H1*–*H2*–*H3*–*H4*. Chamois raw counts usually underestimate real population size (Corlatti et al., 2015), and prior to data analysis, all sex and age classes were adjusted using a coefficient of 0.20 (cf. Corlatti et al., 2019). This adjustment simply allows to provide a closer representation of the “true” population size (though, admittedly, different sex and age classes may have different detection probabilities), and it does not affect the significance of the relationships between demographic parameters and predictors, as the former are calculated as ratios. Furthermore, time‐series count data often contain observational errors that may induce bias in the estimators. To disentangle observation errors from process errors, we therefore filtered the adjusted counts using state‐space models and a Kalman filter approach (Dennis et al., 2006) with the package “MARSS” (Holmes, Ward, & Wills, 2012, 2013). Based on state‐space filtered data, four demographic parameters were defined for chamois and used as our response variables:

1. Birth rate (*Br*) defined the number of kids per female at year [*t*]. *Br* was calculated as the ratio between the number of kids and the number of adult females in the same year: $Br_{\left[t\right]}=\frac{{\text{Number of kids}}_{\left[t\right]}}{{\text{Number of adult females}}_{\left[t\right]}}$. In this form, *Br* includes both fecundity of females and kid survival during their first months of life
2. Kid survival (*K_s*) defined the number of kids that survived between year [*t*] and [*t* + 1]. *K_s* was calculated as the ratio between the number of yearlings at [*t* + 1] and the number of kids at [*t*]: $K\_s_{\left[\left(t+1\right)/t\right]}=\frac{{\text{Number of yearlings}}_{\left[t+1\right]}}{{\text{Number of kids}}_{\left[t\right]}}$. Kid survival was assumed to be unbiased with respect to sex (cf. Bocci et al., 2010; Corlatti et al., in press).
3. Female survival (*F_s*) defined the number of females of 1+ years of age that survived between year [*t*] and [*t* + 1]. *F_s* was calculated as the ratio between the number of adult females (2+ years of age) at [*t* + 1] and the sum of adult females and half the number of yearlings at [*t*]: $F\_s_{\left[\left(t+1\right)/t\right]}=\frac{{\text{Number of adult females}}_{\left[t+1\right]}}{{\text{Number of adult females}}_{\left[t\right]}+1/2*{\text{Number of yearling}}_{\left[t\right]}}$. For yearlings, a balanced sex ratio and an equal survival irrespective of density were assumed (cf. Bocci et al., 2010; Corlatti et al., in press).
4. Male survival (*M_s*) defined the number of males of 1+ years of age that survived between year [*t*] and [*t* + 1]. *M_s* was calculated as the ratio between the number of adult males (2+ years of age) at [*t* + 1] and the sum of number of adult male and half the number of yearlings at [*t*]: $M\_s_{\left[\left(t+1\right)/t\right]}=\frac{{\text{Number of adult males}}_{\left[t+1\right]}}{{\text{Number of adult males}}_{\left[t\right]}+1/2*{\text{Number of yearling}}_{\left[t\right]}}$. For yearlings, a balanced sex ratio and an equal survival irrespective of density were assumed (cf. Bocci et al., 2010; Corlatti et al., in press).

​

To test *H5*, we calculated chamois growth rate as $Y_{\left[\left(t+1\right)/t\right]}=\text{ln}\left(N_{\left[t+1\right]}/N_{\left[t\right]}\right)$, where *Y* represents growth rate; $N_{\left[t+1\right]}$, the overall chamois population size at year [*t* + 1]; and $N_{\left[t\right]}$, the overall chamois population size at [*t*]; growth rate was calculated using state‐space filtered data and excluding kids (Corlatti et al., 2019). Figure 2 shows the temporal trend of the different demographic parameters, along with the temporal trend of red deer abundance.

### 2.4 *H1*–*H2*–*H3*–*H4*: Model fitting, selection, and validation

The hypotheses in Figure 1 require that regression models with different combinations of predictors are contrasted to explore if *H1*, *H2*, *H3*, and *H4* are supported by the data. All analyses were conducted using R *v*. 4.0.4 (R Core Team, 2020) in RStudio *v*. 1.3.1056 (RStudio Team, 2020). First, four “global” Gaussian linear models including birth rate at time [*t*], kid, adult female, and adult male survival between [*t*] and [*t* + 1] as separate response variables were fitted to check for goodness of fit and multicollinearity issues (Burnham & Anderson, 2002). Though our response variables are typically constrained between 0 and 1, their conditional distributions can be considered approximately normal, which allows for a straightforward interpretation of effect size, as opposed to, for example, Beta or binomial distributions. The global model for birth rate included, in the linear predictor, the effect of winter precipitation at time [*t*] and spring–summer precipitation at time [*t* − 1] in interaction with chamois abundance at time [*t* − 1] and deer abundance at time [*t* − 1]. Chamois abundance at time [*t* − 1] was preferred over abundance at [*t*] because the impact of density dependence on maternal conditions at birth was expected to manifest itself with some delay. Furthermore, although birth rate includes kid survival in the first months of life, preliminary analysis revealed that climatic variables such as spring–summer precipitation or temperature at [*t*] did not explain much of the variance in birth rate; thus, they were excluded from the global model. The global models for survival rates included, in the linear predictor, the effect of winter precipitation at time [*t* + 1] and spring–summer precipitation at time [*t*] in interaction with chamois abundance at time [*t*] and deer abundance at time [*t* − 1]. Deer abundance was always modeled with 1‐year delay based on Corlatti et al. (2019): Preliminary analyses on single demographic parameters also supported the use of deer abundance at [*t* − 1] over deer abundance at [*t*]. All models included an autoregressive term with 1‐year lag to account for temporal correlation (Gelman et al., 2020). All continuous predictors were centered and divided by 1 standard deviation prior to analysis.

Given the limited sample size and the complexity of the global models, we adopted a Bayesian estimation approach, which is not based on asymptotic assumptions and permits the making of inference without losing much power even in small samples (cf. Hox et al., 2012; van de Schoot & Miočević, 2020). The joint posterior distribution of regression coefficients was estimated via Markov chain Monte Carlo (MCMC) with 20,000 sampling iterations over 4 chains (including 1,000 warm‐ups for each chain), using the No‐U‐Turn Sampler (Hoffman & Gelman, 2014) with default priors in the package “rstanarm” (Goodrich et al., 2020). The goodness of fit of each global model was assessed through inspection of R‐hat values, trace plots, and posterior‐predictive checks using the package “shinystan” (Gabry, 2018). We also inspected quantile residual diagnostics (residuals vs. model predictions, residuals vs. single predictors) and checked for temporal autocorrelation using the Durbin–Watson test for nonindependence in time series (Durbin & Watson, 1971) with the package “DHARMa” (Hartig, 2020). Multicollinearity was inspected with the “vif” (variance inflation factor) function in the “car” package (Fox & Weisberg, 2019). VIF values <3 were considered inconsequential (cf. Zuur et al., 2010).

Starting from the global models, a set of 19 simpler models was built for each response variable, to reflect contrasting biological patterns and thus assess the plausibility of hypotheses *H1*, *H2*, *H3*, and *H4* (cf. Burnham & Anderson, 2002). Given the limited sample size, for each model we did not include more than three explanatory variables or one interactive term, to avoid issues in parameter estimation (assuming large effect size, the formula proposed by Green[1991] suggests that a sample size of *n* = 28 should suffice to estimate regression coefficients for up to five independent variables). The full list of models, along with the respective hypotheses, is reported in the Supplementary file. To select which of the 19 linear models best explained variation in each of our response variable, we compared different model selection procedures, as model selection may be particularly challenging when sample size is small. First, the 19 Gaussian linear models were fitted using the same Bayesian approach adopted for the global models. Bayesian models were ranked according to their value of Watanabe–Akaike information criterion (WAIC), and leave‐one‐out cross‐validation (LOO‐CV). WAIC is a generalized version of the AIC (Watanabe, 2013), while LOO‐CV is a measure of predictive accuracy obtained by training the model on *n* − 1 observations from the dataset, and validating the model on the remaining observations, repeating this procedure for all observations in the dataset (Vehtari et al., 2017). Next, the same 19 models were refitted using a classic ordinary least‐squares approach, using the “lm” function in the “stats” package (R Core Team, 2020), and subsequently ranked according to their values of Akaike information criterion corrected for small samples (AICc: Hurvich & Tsai, 1989) with the package “MuMIn” (Bartoń, 2020). Finally, the same ordinary least‐squares models were ranked according to their value of bias‐corrected root‐mean‐squared error (RMSE, a measure of predictive accuracy) using an internal validation bootstrap approach following the procedure described in Steyerberg et al. (2001) with the “rms” package (Harrell, 2020) (cf. Supplementary file for more details). Within the respective framework (Bayesian and frequentist), these selection methods should be asymptotically equivalent (cf. Stone, 1977; Watanabe, 2010); however, differences can arise with limited sample sizes, and the results of different model selection procedures were checked for consistency. These model selection methods are expected to reduce the issues of overfitting, by penalizing the model for adding extra terms (WAIC, AICc) or by testing the performance of a trained model on test data (LOO‐CV, bias‐corrected RMSE).

Given the general consensus among different selection methods and the numerical consistency between Bayesian and frequentist estimates (cf. Section 3), to account for model selection uncertainty we used the simplest pragmatic approach and averaged regression coefficients (mean and 95% confidence intervals) of ordinary least‐squares models that received substantial support from the data, that is, with delta AICc < 4, using Akaike's weights (cf. Burnham & Anderson, 2002). Marginal effects for significant predictors were plotted using the “visreg” package (Breheny & Burchett, 2017). The goodness of fit of averaged models was assessed through residual diagnostics, and their *R* ^2^ values were calculated with the “performance” package (Lüdecke et al., 2020).

### 2.5 *H5*: Model fitting, selection, and validation

To explore *H5*, that is, the relationships between red deer abundance and chamois growth rate mediated by demographic parameters, four different directed acyclic graphs were built to represent direct and indirect relationships (sensu Shipley, 2016) among the selected variables, thereby reflecting alternative biologically plausible causal chains. In the different models, we hypothesized that red deer abundance at time [*t* − 1] had an indirect negative effect on chamois population growth rate between [*t*] and [*t* + 1] mediated by (a) birth rate at time [*t*]; (b) kid survival between [*t*] and [*t* + 1]; (c) adult female survival between [*t*] and [*t* + 1]; and (d) adult male survival between [*t*] and [*t* + 1]. Model complexity was kept at a minimum (i.e., not including climatic variables, or more than one vital rate at a time) because of limited sample size: Rules of thumbs suggest that at least 10 data points for each variable are needed in causal analysis (Wolf et al., 2013). This analysis was expected to support the results of the regression models selected to explore *H1*–*H2*–*H3*–*H4*.

Alternative direct/indirect relationships among demographic parameters were tested using path analysis (Wright, 1934) with the package “lavaan” (Rosseel, 2012), assuming a normal conditional distribution for growth rate (cf. Corlatti et al. 2019). To select the best‐fitting model, we ranked the models according to their value of AIC. The consistency of the correlational structures between the hypothesized models and the sample data was assessed with a chi‐square goodness‐of‐fit test and the value of the root‐mean‐square error of approximation (RMSEA: Steiger & Lind, 1980).

The full dataset, along with a detailed description of all statistical analyses, is available in the Supplementary file.

## 3 RESULTS

### 3.1 *H1*–*H2*–*H3*–*H4*: Model validation and selection

All the global models showed no issues of MCMC convergence, as suggested by the trace plots and the values of R‐hat (all <1.02, cf. Brooks & Gelman, 1998). The posterior‐predictive checks showed that all global models predicted the actual response variable well. The residual diagnostics for the global models suggested no major violations of assumptions and nonsignificant values of the Durbin–Watson test for 1‐year lag temporal autocorrelation (birth rate: D–W = 2.520, *p*‐value = 0.157; kid survival: D–W = 1.766, *p*‐value = 0.537; adult female survival: D–W = 2.419, *p*‐value = 0.265; adult male survival: D–W = 1.789, *p*‐value = 0.578).

The 4 model selection methods (AICc, RMSE, WAIC, and LOO‐CV) showed consistency in the top‐ranked models (Table 1). Preliminary inspection of results also showed numerically consistent regression coefficient estimates between frequentist and Bayesian models (cf. Supplementary file): For final inference, we therefore relied on the least‐squares models with ΔAICc < 4. Table 2 reports the structure and biological hypotheses associated with the selected models. The residual diagnostics for all the final models did not suggest violation of assumptions (cf. Supplementary file).

**TABLE 1:** Rank of models fitted to explain variation in chamois demographic parameters within the Stelvio National Park between 1993 and 2020

Parameter	AICc		*R* ^2^	RMSE		WAIC			LOO‐CV		
	Model name	Delta AICc		Model name	RMSE	Model name	Elpd diff.	*SE* diff.	Model name	Elpd diff.	*SE* diff.
*Birth rate*_[_ *_t_*_]_	m.4.br	0	0.74	m.4.br	0.041	m.4.br	0	0	m.4.br	0	0
	m.6.br	2.25	0.69	m.6.br	0.042	m.6.br	−1.19	2.25	m.6.br	−0.32	2.58
*Kid survival*_[(_ *_t_*_+1)/_ *_t_*_]_	m.17.ks	0	0.36	m.12.ks	0.037	m.13.ks	0	0	m.13.ks	0	0
	m.13.ks	0.74	0.44	m.13.ks	0.037	m.1.ks	−0.73	0.71	m.17.ks	−0.91	2.08
	m.12.ks	1.6	0.32	m.18.ks	0.037	m.17.ks	−1.43	2.29	m.18.ks	−1.04	1.98
	m.18.ks	1.77	0.32	m.19.ks	0.037	m.18.ks	−1.53	1.9	m.1.ks	−1.25	0.8
	m.19.ks	1.82	0.32	m.1.ks	0.038	m.12.ks	−1.63	2.04	m.19.ks	−1.27	2.15
	m.9.ks	3.02	0.34	m.6.ks	0.038	m.19.ks	−1.71	2.06	m.12.ks	−1.29	2.17
	m.15.ks	3.04	0.34	m.10.ks	0.038	m.6.ks	−2.18	2.38	m.6.ks	−1.86	2.48
	m.1.ks	3.79	0.42	m.17.ks	0.038	m.9.ks	−2.43	2.21	m.15.ks	−2.15	2.06
*Female survival*_[(_ *_t_*_+1)/_ *_t_*_]_	m.1.fs	0	0.57	m.1.fs	0.065	m.1.fs	0	0	m.1.fs	0	0
*Male survival*_[(_ *_t_*_+1)/_ *_t_*_]_	m.11.ms	0	0.32	m.11.ms	0.072	m.11.ms	0	0	m.11.ms	0	0
	m.12.ms	2.11	0.21	m.8.ms	0.074	m.8.ms	−0.77	1.16	m.8.ms	−0.16	1.71
	m.8.ms	2.72	0.3	m.7.ms	0.075	m.7.ms	−1.09	0.38	m.5.ms	−0.81	3.39
	m.7.ms	2.99	0.3	m.2.ms	0.076	m.2.ms	−1.23	2.1	m.7.ms	−1.29	0.47
	m.5.ms	3.84	0.27	m.12.ms	0.076	m.1.ms	−1.61	0.97	m.12.ms	−1.51	2.13

Note

For each parameter, the table reports the name of the model, the difference in Akaike information criterion corrected for small samples (delta AICc), optimism‐corrected root‐mean‐square error (RMSE), difference in Watanabe Akaike information criterion (WAIC), and leave‐one‐out cross‐validation (LOO‐CV), including difference in *SE* for the latter two. Only models with delta AICc < 4 were selected for final inference, and they are reported with their explained variance (adjusted *R* ^2^). Model names with the same number have the same structure. For more details, see Supplementary file.

**TABLE 2:** Structure and hypothesized biological mechanisms of the models selected to explain variation in chamois demographic parameters within the Stelvio National Park between 1993 and 2020

Model structure	Hypothesized biological mechanism
*Birth rate*_[*t*]_	
m.4.br: $Br_{\left[t\right]}∼Br_{\left[t‐1\right]}+P_{\text{spring}‐\text{summer}\;\left[t‐1\right]}\times N\_{\text{deer}}_{\left[t‐1\right]}+N\_{\text{chamois}}_{\left[t‐1\right]}$	Medium‐term interactive effect of spring–summer precipitation and red deer abundance, and medium‐term effect of chamois abundance on female body condition
m.6.br: $Br_{\left[t\right]}∼Br_{\left[t‐1\right]}+P_{\text{spring}‐\text{summer}\;\left[t‐1\right]}\times N\_{\text{deer}}_{\left[t‐1\right]}$	Medium‐term interactive effect of spring–summer precipitation and red deer abundance on female body condition
*Kid survival*_[(_ *_t_*_+1)/_ *_t_*_]_	
m.17.ks: $K\_s_{\left[\left(t+1\right)/t\right]}∼K\_s_{\left[t/\left(t‐1\right)\right]}+P_{\text{winter}\;\left[t+1\right]}$	Short‐term effect of winter precipitation on kid body condition
m.13.ks: $K\_s_{\left[\left(t+1\right)/t\right]}∼K\_s_{\left[t/\left(t‐1\right)\right]}+P_{\text{winter}\;\left[t+1\right]}\times N\_{\text{chamois}}_{\left[t\right]}$	Interaction between short‐term effect of winter precipitation and medium‐term effect of chamois abundance on kid or female (i.e., maternal) body condition
m.12.ks: $K\_s_{\left[\left(t+1\right)/t\right]}∼K\_s_{\left[t/\left(t‐1\right)\right]}+N\_{\text{deer}}_{\left[t‐1\right]}$	Long‐term effect of red deer abundance on female body condition
m.18.ks: $K\_s_{\left[\left(t+1\right)/t\right]}∼K\_s_{\left[t/\left(t‐1\right)\right]}+P_{\text{spring}‐\text{summer}\;\left[t\right]}$	Short‐term effect of spring–summer precipitation on kid or female (i.e., maternal) body condition
m.19.ks: $K\_s_{\left[\left(t+1\right)/t\right]}∼K\_s_{\left[t/\left(t‐1\right)\right]}+N\_{\text{chamois}}_{\left[t\right]}$	Medium‐term effect of chamois abundance on kid or female (i.e., maternal) body condition
m.9.ks:$K\_s_{\left[\left(t+1\right)/t\right]}∼K\_s_{\left[t/\left(t‐1\right)\right]}+P_{\text{winter}\;\left[t+1\right]}+N\_{\text{deer}}_{\left[t‐1\right]}$	Short‐term effect of winter precipitation on kid body condition and long‐term effect of red deer abundance on female (i.e., maternal) body condition
m.15.ks: $K\_s_{\left[\left(t+1\right)/t\right]}∼K\_s_{\left[t/\left(t‐1\right)\right]}+P_{\text{winter}\;\left[t+1\right]}+N\_{\text{chamois}}_{\left[t\right]}$.	Short‐term effect of winter precipitation on kid body condition and medium‐term effect of chamois abundance on kid or female (i.e., maternal) body condition
m.1.ks: $K\_s_{\left[\left(t+1\right)/t\right]}∼K\_s_{\left[t/\left(t‐1\right)\right]}+P_{\text{winter}\;\left[t+1\right]}\times N\_{\text{chamois}}_{\left[t\right]}+N\_{\text{deer}}_{\left[t‐1\right]}$	Interaction between short‐term effect of winter precipitation and medium‐term effect of chamois abundance on kid or female (i.e., maternal) body condition, and long‐term effect of red deer abundance on female (i.e., maternal) body condition
*Female survival*_[(_ *_t_*_+1)/_ *_t_*_]_	
m.1.fs: $F\_s_{\left[\left(t+1\right)/t\right]}∼F\_s_{\left[t/\left(t‐1\right)\right]}+P_{\text{winter}\left[t+1\right]}\times N\_{\text{chamois}}_{\left[t\right]}+N\_{\text{deer}}_{\left[t‐1\right]}$.	Interaction between short‐term effect of winter precipitation and medium‐term effect of chamois abundance on female body condition, and long‐term effect of red deer abundance on female body condition
*Male survival*_[(_ *_t_*_+1)/_ *_t_*_]_	
m.11.ms: $M\_s_{\left[\left(t+1\right)/t\right]}∼M\_s_{\left[t/\left(t‐1\right)\right]}+N\_{\text{deer}}_{\left[t‐1\right]}+N\_{\text{chamois}}_{\left[t\right]}$	Long‐term effect of red deer abundance and medium‐term effect of chamois abundance on male body condition
m.12.ms: $M\_s_{\left[\left(t+1\right)/t\right]}∼M\_s_{\left[t/\left(t‐1\right)\right]}+N\_{\text{deer}}_{\left[t‐1\right]}$.	Long‐term effect of red deer abundance on male body condition
m.8.ms: $M\_s_{\left[\left(t+1\right)/t\right]}∼M\_s_{\left[t/\left(t‐1\right)\right]}+P_{\text{spring}‐\text{summer}\left[t\right]}+N\_{\text{deer}}_{\left[t‐1\right]}+N\_{\text{chamois}}_{\left[t\right]}$	Medium‐term effect of spring–summer precipitation and chamois abundance and long‐term effect of red deer abundance on male body condition
m.7.ms: $M\_s_{\left[\left(t+1\right)/t\right]}∼M\_s_{\left[t/\left(t‐1\right)\right]}+P_{\text{winter}\left[t+1\right]}+N_{\text{deer}\left[t‐1\right]}+N_{\text{chamois}\left[t\right]}$	Short‐term effect of winter precipitation, medium‐term effect of chamois abundance, and long‐term effect of red deer abundance on male body condition
m.5.ms: $M\_s_{\left[\left(t+1\right)/t\right]}∼M\_s_{\left[t/\left(t‐1\right)\right]}+P_{\text{winter}\;\left[t+1\right]}\times N\_{\text{deer}}_{\left[t‐1\right]}$	Interaction between short‐term effect of winter precipitation and long‐term effect of deer abundance on male body condition

Note

The table reports only models with delta AICc < 4: For each demographic parameter, the structure and the biological meaning of the model are described. Each model includes an autoregressive term. The influence of the explanatory variables on the response variable has been indicated as a “short‐term,” “medium‐term,” and “long‐term” effect when it was expected to occur, respectively, with 0‐, 1‐, or 2‐year time lag.

### 3.2 *H1*–*H2*–*H3*–*H4*: Model results

**FIGURE 3:**
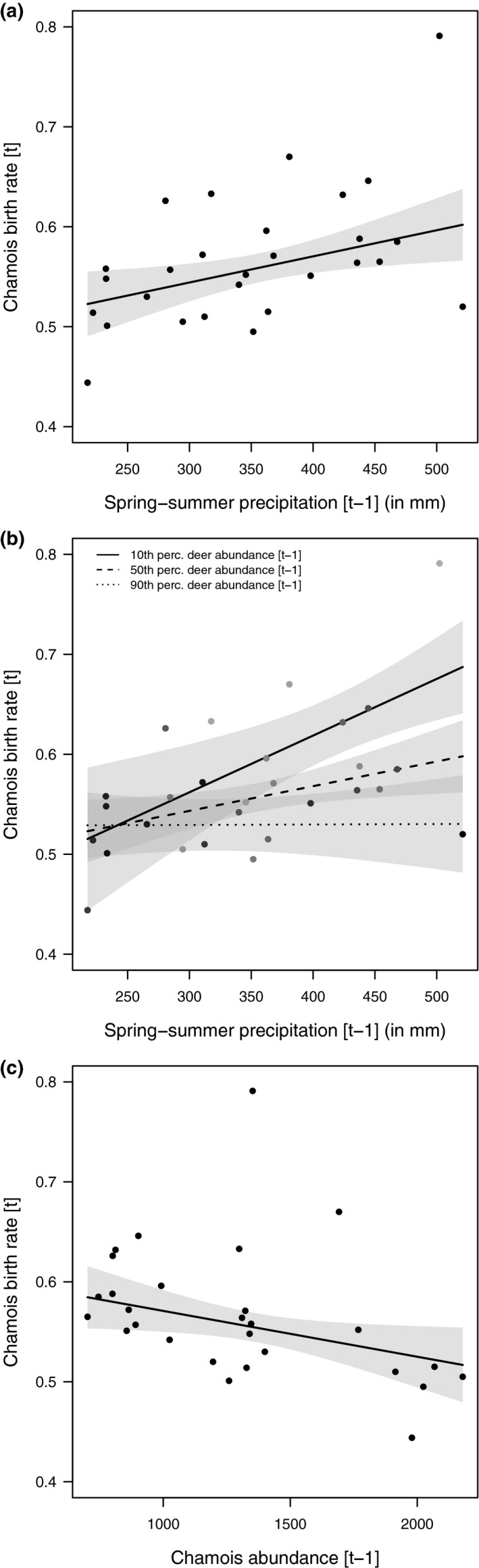
Marginal effects of the models selected to explain variation in chamois birth rate within the Stelvio National Park between 1993 and 2020. In panel a, birth rate at time [*t*] as a linear function of spring–summer precipitation at [*t* − 1] (in mm). In panel b, birth rate at [*t*] as a linear function of the interactive effect between spring–summer precipitation (in mm) and red deer abundance at [*t* − 1] expressed in percentiles (10th, 50th, 90th); thicker lines indicate low red deer abundance, and dashed line indicates higher red deer abundance. In panel c, birth rate at [*t*] as a linear function of chamois abundance at [*t* − 1]. Linear regression lines are reported with 95% confidence interval (gray shaded area)

**FIGURE 4:**
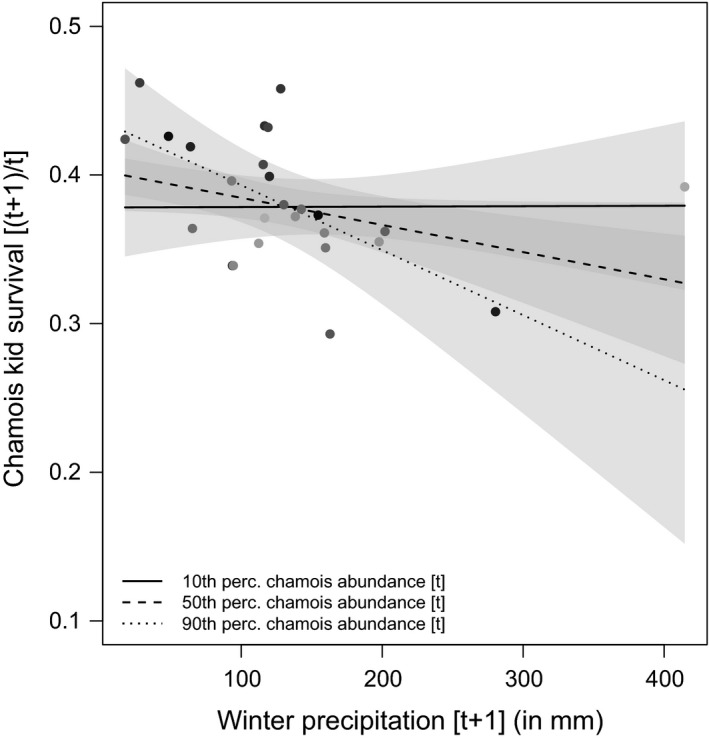
Marginal effects of the models selected to explain variation in chamois kid survival within the Stelvio National Park between 1993 and 2020. Kid survival between time [*t*] and [*t* + 1] is a linear function of the interactive effect between winter precipitation (in mm) at [*t* + 1] and chamois abundance at [*t*]. Chamois abundance is expressed in percentiles (10th, 50th, 90th): Thicker lines indicate lower chamois abundance, while dashed line indicates higher chamois abundance. Linear regression lines are reported with 95% confidence interval (gray shaded area)

**FIGURE 5:**
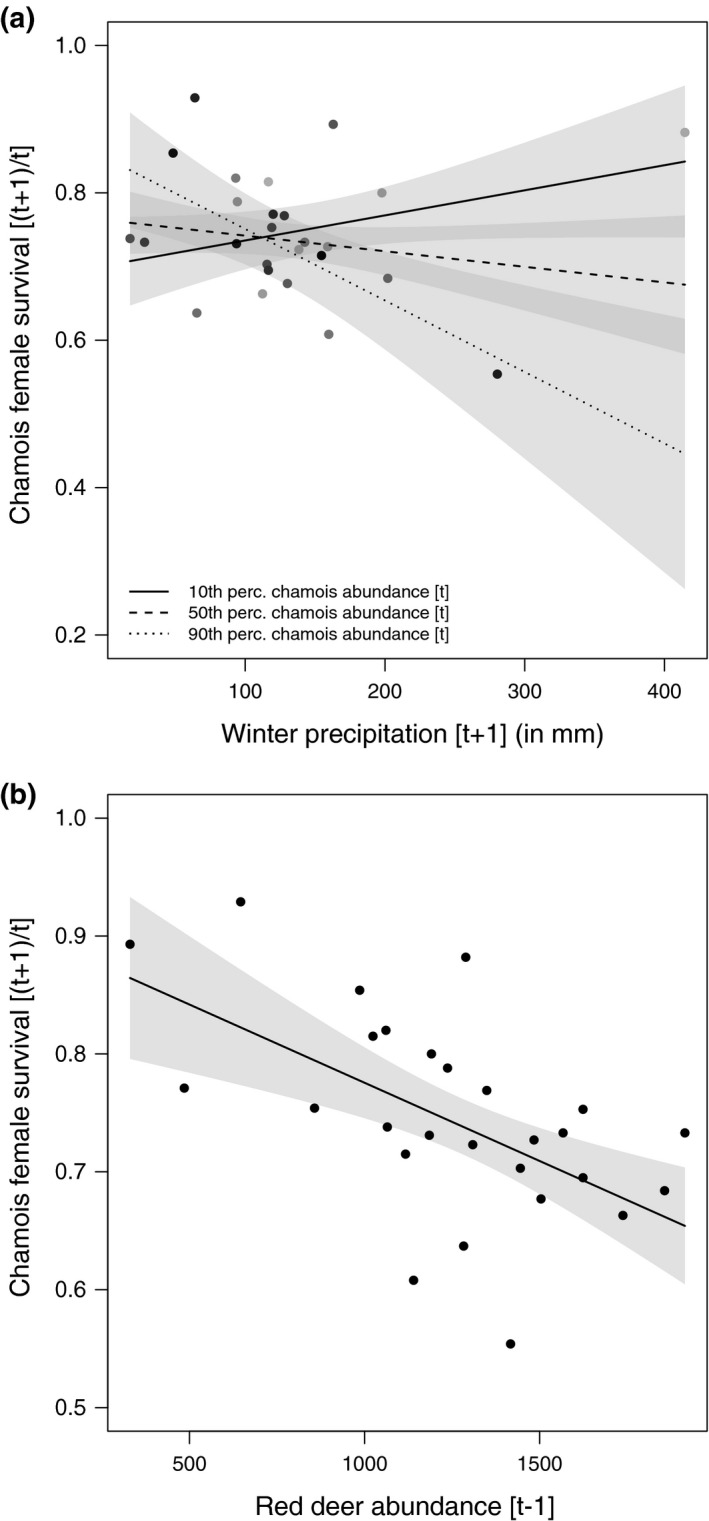
Marginal effects of the models selected to explain variation in chamois adult female survival within the Stelvio National Park between 1993 and 2020. In panel a, female survival between time [*t*] and [*t* + 1] as a linear function of the interactive effect between winter precipitation (in mm) at [*t* + 1] and chamois abundance at [*t*]. Chamois abundance is expressed in percentiles (10th, 50th, 90th): Thicker lines indicate low chamois abundance, while dashed line indicates higher chamois abundance. In panel b, female survival between [*t*] and [*t* + 1] as a linear function of red deer abundance at [*t* − 1]. Linear regression lines are reported with 95% confidence interval (gray shaded area)

**FIGURE 6:**
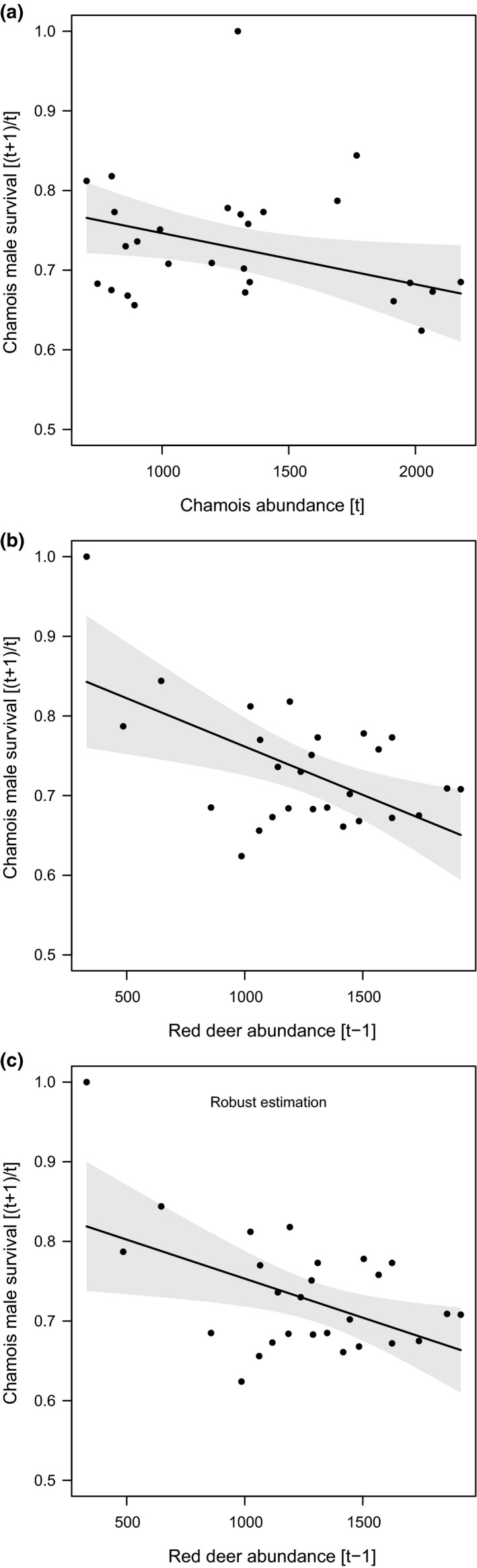
Marginal effects of the models selected to explain variation in chamois adult male survival within the Stelvio National Park between 1993 and 2020. In panel a, male survival between time [*t*] and [*t* + 1] as a linear function of chamois abundance at [*t*]. In panel b, male survival between [*t*] and [*t* + 1] as a linear function of red deer abundance at [*t* − 1]. Linear regression lines are reported with 95% confidence interval (gray shaded area). In panel b, a data point might influence the slope of the regression line. A robust linear approach (panel c) showed no evidence of significant change in the relationship

The selected models for birth rate at year [*t*] included spring–summer precipitation at [*t* − 1], chamois and red deer abundance at [*t* − 1], and the interaction between spring–summer precipitation and red deer abundance (Table 2). Averaged parameter estimates showed that chamois birth rate had a positive and significant relationship with spring–summer precipitation, and significantly negative relationships with chamois abundance and with the combination of spring–summer precipitation and red deer (Table 3; Figure 3). Kid survival model selection showed a relatively high uncertainty, and the selected models included spring–summer precipitation at [*t*], winter precipitation at [*t* + 1], red deer abundance at [*t* − 1], chamois abundance at [*t*], and the interaction between winter precipitation and chamois abundance (Table 2). Averaged parameter estimates showed that only the combined effect of winter precipitation and chamois abundance had a significant negative relationship with kid survival (Table 3; Figure 4). For female survival, only one model was selected, which included winter precipitation at [*t* + 1], red deer abundance at [*t* − 1], chamois abundance at [*t*], and the interaction between winter precipitation and chamois abundance (Table 2). Averaged parameter estimates showed that adult female survival had a significant negative relationship with red deer abundance and with the combination of winter precipitation and chamois abundance (Table 3; Figure 5). For kid and female survival, the presence of a single large data point for winter precipitation suggested potential issues of high leverage. When the single data point was removed, the interaction between abundance and winter precipitation remained significant for females, but not for kids, whose survival was affected negatively by precipitation only. The selected model for male survival included spring–summer precipitation at [*t*], winter precipitation at [*t* + 1], red deer abundance at [*t* − 1], chamois abundance at [*t*], and the interaction between winter precipitation and deer abundance (Table 2). Only red deer and chamois abundance, however, had a significant negative relationship with male survival (Table 3; Figure 6). In Figure 6b, a data point suggests a potential influence on the slope of the regression line between red deer abundance and male survival: A robust linear modeling approach using the “robustbase” package (Maechler et al., 2020), however, showed that the negative relationship remained significant even when accounting for the outlier (*ß* = −0.037; 95% CI = −0.066, −0.007) (Figure 6c). Notably, for all demographic parameters, interactive models were selected over the corresponding additive alternatives by all model selection techniques (cf. Supplementary file), thereby supporting the absence of overfitting issues when interaction terms were included in the models.

**TABLE 3:** Averaged parameter estimates of models with delta AICc <4, selected to explain variation in chamois demographic parameters within the Stelvio National Park between 1993 and 2020

Parameter	Estimate	*SE*	95 LCL	95 UCL
*Birth rate*_[*t*]_				
Intercept	0.561	0.007	0.546	0.575
Birth rate _[_ *_t_*_−1]_	0.016	0.012	−0.007	0.040
***P*_spring–summer [_*_t_*_−1]_**	**0.026**	**0.010**	**0.006**	**0.046**
*N*_deer _[_ *_t_*_−1]_	−0.022	0.013	−0.048	0.004
***N*_chamois _[_*_t_*_−1]_**	**−0.021**	**0.010**	**−0.039**	**−0.002**
***N*_deer _[_*_t_*_−1]_** × ***P*_spring–summer [_*_t_*_−1]_**	**−0.022**	**0.007**	**−0.035**	**−0.008**
*Kid survival*_[(_ *_t_*_+1)/_ *_t_*_]_				
Intercept	0.382	0.007	0.369	0.396
**Kid survival _[_*_t_*_−1]_**	**0.022**	**0.008**	**0.007**	**0.037**
*P*_spring_ **_–_**_summer__[*t*]_	−0.002	0.007	−0.017	0.012
*P*_winter [_ *_t_*_+1]_	−0.011	0.008	−0.026	0.004
*N*_deer _[_ *_t_*_−1]_	−0.001	0.008	−0.018	0.015
*N*_chamois _[*t*]_	0.001	0.007	−0.013	0.015
***N*_chamois**_[*t*]_ × ***P*_winter [_*_t_*_+1]_**	**−0.014**	**0.006**	**−0.026**	**−0.001**
*Female survival*_[(_ *_t_*_+1)/_ *_t_*_]_				
Intercept	0.737	0.011	0.714	0.760
Female survival _[_ *_t_*_−1]_	−0.023	0.013	−0.049	0.003
*P*_winter [_ *_t_*_+1]_	−0.043	0.012	−0.039	0.011
***N*_deer _[_*_t_*_−1]_**	**−0.050**	**0.012**	**−0.076**	**−0.024**
*N*_chamois _[*t*]_	−0.010	0.012	−0.035	0.016
***N*_chamois**_[*t*]_ × ***P*_winter [_*_t_*_+1]_**	**−0.040**	**0.011**	**−0.062**	**−0.017**
*Male survival*_[(_ *_t_*_+1)/_ *_t_*_]_				
Intercept	0.736	0.013	0.709	0.762
Male survival _[_ *_t_*_−1]_	0.007	0.015	−0.024	0.037
*P*_spring_ **_–_**_summer__[*t*]_	−0.010	0.014	−0.038	0.019
*P*_winter [_ *_t_*_+1]_	−0.003	0.015	−0.032	0.026
***N*_deer _[_*_t_*_−1]_**	**−0.043**	**0.016**	**−0.075**	**−0.011**
***N*_chamois**_[*t*]_	**−0.030**	**0.015**	**−0.059**	**−0.002**
*N*_deer _[_ *_t_*_−1]_ × *P*_winter [_ *_t_*_+1]_	−0.047	0.025	−0.096	0.001

Note

The table reports, for each parameter, the standardized ordinary least‐squares regression coefficient estimates (estimate), standard errors (*SE*), lower 95% confidence level (95 LCL), and upper 95% confidence level (95 UCL). Statistically significant predictors in bold.

### 3.3 *H5*: Path analysis

**FIGURE 7:**
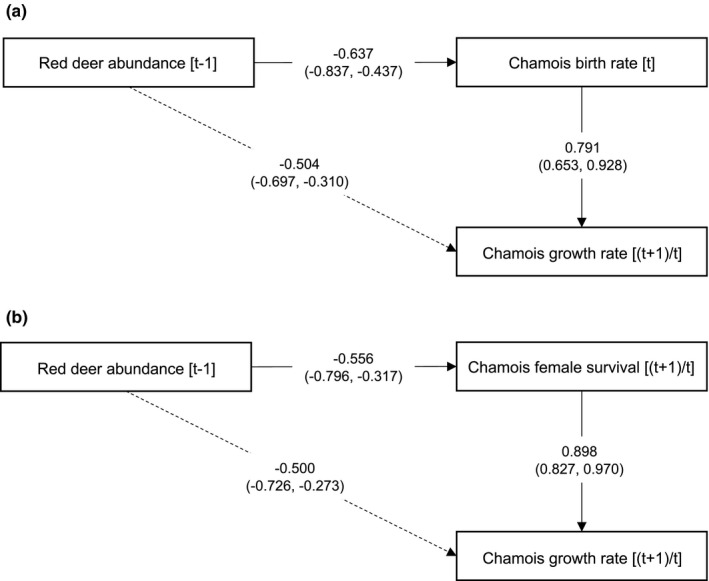
Standardized coefficients and 95% confidence interval for the direct and indirect relationships assumed in the path models selected to explain variation in chamois population growth rate within the Stelvio National Park between 1993 and 2020. Dashed lines indicate the red deer indirect effect on chamois population growth rate, mediated by birth rate (a) and female survival (b)

The path analysis showed that only two of the four models satisfied the correlational structure of the data ($\chi^{2}$ *p*‐value >0.05 and RMSEA value ≤ 0.6, cf. Tomer & Pugesek, 2003) (Table 4). Model a and Model c supported, at least partially, the results of the linear model selection and showed that red deer population size at [*t* − 1] had an indirect relationship with chamois population growth rate mediated by chamois birth rate and chamois female survival. The size of the indirect relationship through birth rate (*ß* = −0.504; 95% CI = −0.697, −0.310) was very similar to that hypothesized through female survival (*ß* = −0.500; 95% CI = −0.726, −0.273) (Figure 7). This result is also supported by the very similar values of AIC returned by the two models (Table 4).

**TABLE 4:** Path models fitted to explain the indirect relationship between red deer abundance and chamois growth rate, mediated by birth rate (Model a), kid survival (Model b), adult female survival (Model c), and adult male survival (Model d), within the Stelvio National Park between 1993 and 2020

Model	$\chi^{2}$	*df*	$\chi^{2}$ *p*‐value	RMSEA	RMSEA *p*‐value	AIC
**Model a**	**0.529**	**1**	**0.467**	**0.000**	**0.482**	**−140.4**
Model b	13.027	1	0.000	0.667	0.000	−128.8
**Model c**	**1.010**	**1**	**0.315**	**0.020**	**0.331**	**−142.2**
Model d	4.082	1	0.043	0.338	0.050	−114.5

The table reports, for each model, the chi‐square goodness‐of‐fit test ($\chi^{2}$), degrees of freedom (*df*), *p*‐values for the chi‐square test ($\chi^{2}$ *p*‐value), root‐mean‐square error of approximation (RMSEA), *p*‐values for RMSEA (RMSEA *p*‐values), and Akaike information criterion (AIC). Selected models in bold.

## 4 DISCUSSION

Our results suggest that variation in chamois vital rates was largely explained by weather stochasticity, density dependence, and interspecific competition with red deer. In particular, spring–summer precipitation was positively related to birth rate, while the combination of high winter precipitation and chamois abundance was negatively related to kid and adult female survival. Plain negative density dependence was detected in birth rate and adult male survival, while red deer abundance related negatively to all demographic parameters, except kid survival. The path analysis supported an indirect negative relationship of red deer abundance with chamois population growth rate, mediated by birth rate and adult female survival.

Environmental conditions in mountain habitats are very heterogeneous in space and in time. At high elevations, summer foraging is important for mountain ungulates to secure energy acquisition before winter, and a short vegetative season may affect key life‐history traits such as growth, reproduction, and survival (Anderwald et al., 2016; Côté & Festa‐Bianchet, 2001; Pettorelli et al., 2007; Willisch et al., 2013). Previous studies have emphasized the importance of weather conditions in spring–summer for survival and female reproductive success (Grøtan et al., 2008; Rughetti & Festa‐Bianchet, 2012; White et al., 2011). Higher spring–summer temperatures, for example, may lead to a shorter growing season of plants with negative consequences on yearling body mass, which may possibly hamper survival (Loison et al., 1999; Rughetti & Festa‐Bianchet, 2012). Furthermore, higher temperature coupled with lower rainfall would limit the nutrient supply to plants (Li et al., 2018), with consequent negative effects on the nutritional quality of forage available to females and offspring, ultimately affecting energy intake and survival (Ferretti et al., 2019; Garel et al., 2004; Loison et al., 1999). Thus, rainy springs are expected to allow females to face mating, gestation, and calving in better body conditions, possibly increasing their reproductive success through, for example, increased fecundity (cf. Albon et al., 1986) and higher kid survival (Côté & Festa‐Bianchet, 2001; Pettorelli et al., 2007). In our study area, birth rate was positively influenced by spring–summer precipitation in the previous year, but not in the same year (see methods); this suggests an indirect effect of spring–summer weather on birth rate through improved maternal conditions (cf. Chirichella et al., 2021; see also Corlatti et al., 2018 for red deer).

Winter harshness may also shape population dynamics of mountain ungulates through, for example, direct mortality under avalanches, or food limitation (e.g., Alpine ibex *Capra ibex*: Jacobson et al., 2004; Grøtan et al., 2008; chamois: Jonas et al., 2008; Rughetti et al., 2011; Mountain goat *Oreamnus americanus*: White et al., 2011). Our results suggested a negative interactive effect of winter precipitation and density in the previous year on female and kid survival, whereas no effect was supported on male survival or birth rate. The removal of a single data point with high levels of winter precipitation did not alter the results for females, while the interactive effect disappeared in models of kid survival. However, we argue that the removal of this data point is not biologically justified as chamois population dynamics is well known to be largely influenced by heavy snowfalls (Rughetti et al., 2011). Our results support previous studies on chamois. For example, Willisch et al. (2013) found a negative effect of winter harshness on survival of kids, but no effect on birth rate, as in this study. No significant relationship between winter severity and birth rate was found by Chirichella et al. (2021), as in this study. It should be noted that local conditions may affect individual‐to‐population responses to environmental variables, thereby allowing for site‐specific variations of demographic patterns in relation to weather (cf. Loison et al., 1999; Bleu et al., 2015). These results, however, generally support the importance of winter weather stochasticity and density dependence in shaping variation in chamois survival.

Density dependence largely affects population dynamics of mountain ungulates, as it may lead to competition for food resources, with consequent decline of body conditions and lower survival probabilities (Bonenfant et al., 2009; Willisch et al., 2013). Increased densities also tend to delay the age at first reproduction and increase costs of reproduction (Fowler, 1987). In ungulates, however, density dependence is not expected to affect demographic parameters equally (Bonenfant et al., 2009). Increasing abundance has been suggested to trigger first an increase in the age of primiparity, followed by increased kid mortality, yearling mortality, and, with very high density, decreased adult fecundity and survival (Gaillard et al., 2000; Lack, 1966). Our results suggest a negative effect of density dependence on survival of adult females and kids at the highest precipitation levels and on birth rate in the following year, possibly owing to negative effects on maternal physical conditions, as well as on male survival. Notably, adult female survival as defined in this study was more variable than kid survival, which seems unlikely in an ungulate population (cf. Gaillard et al., 2000). It seems plausible that part of this variation in females may owe to the inclusion of yearlings in the calculation and that most of the variation in kid survival may have occurred before 1993 (cf. Figure 1 in Corlatti et al., 2019).

Besides density dependence, red deer abundance may also influence chamois demographic parameters. An additive effect of adverse climatic conditions (high temperatures and low rainfall during the growing season of vegetation) and competition with red deer, for example, was found to hamper female foraging behavior and kid survival in Apennine chamois (Ferretti et al., 2019), with potential long‐term effects on population dynamics (Lovari et al., 2020). Here, the positive relationship between spring–summer precipitation and birth rate was hampered by increasing deer abundance. This suggests that the negative relationship between red deer and birth rate in our study population may be explained by a combination of interspecific competition and weather stochasticity that act synergistically to worsen female body conditions. Female survival and male survival were also negatively related to red deer abundance, and the path analysis suggested that the effect of increasing red deer abundance on adult female survival and birth rate was similar: As these vital rates are expected to decrease at very high density (cf. Lack, 1966), the occurrence of intense interspecific competition seems supported. The lack of effect of red deer abundance on kid survival, however, would militate against this hypothesis. It should be noted that our data were available from 1993 only, and the negative effect of red deer on kid survival might have occurred before, making it difficult to detect density‐dependent variations in this vital rate with our data. Path analysis showed no support for a negative effect of red deer abundance on male survival, although this effect was detected in the regression analysis. Overall, these results suggest that demographic growth was largely driven by the female segment of the population, as often occurs in ungulate species where the effect of male survival on population growth rate is limited (cf. Gaillard et al., 2000). Nonetheless, some caution is needed when interpreting these results, as our path models were rather simplistic: More complex models including all demographic parameters would be desirable, but they require much larger datasets (cf. Wolf et al., 2013).

Several studies have emphasized the potential for competition between red deer and chamois (e.g., Anderwald et al., 2016; Bertolino et al., 2009; Corlatti et al., 2019; Lovari et al., 2014; Schröder & Schröder, 1984). Although mechanisms of competition between red deer and chamois in our study area should be assessed (i.e., exploitation vs. interference), preliminary hypotheses could be put forward. The use of grasslands by red deer at high densities may lead to exploitation of key resources for chamois, with negative consequences on female foraging behavior, body condition, maternal care, and kid survival (Ferretti et al., 2015; Lovari et al., 2014; Scornavacca et al., 2016), or on birth rate (this study). Similarly, a study carried out in the Swiss Alps showed that high red deer densities at high altitudes hampered the breeding success of chamois females (Gamelon et al., 2020). Alternatively, or concurrently, the presence of red deer may displace chamois to suboptimal areas where the latter would face a consequent reduction in the use of high‐quality forage (Anderwald et al., 2016; cf. Chirichella et al., 2013, for interactions between chamois and mouflon). Both mechanisms may not be mutually exclusive in ultimately acting on the use of food resources for chamois, possibly affecting key life‐history traits (cf. Ferretti et al., 2015).

Our study helped unraveling a complex combination of extrinsic and intrinsic factors acting differentially on chamois demographic parameters. Although we found support for a strong negative effect of red deer abundance, density dependence and winter harshness on chamois demographic parameters, especially on the kid–female segment of the population, other drivers may impact chamois life traits, including vegetation productivity (Lovari et al., 2020) or climate‐induced altitudinal shifts (Büntgen et al., 2017). Furthermore, a potential major limitation of the study is the inclusion of yearlings in the survival estimates for adult females and adult males. While unavoidable due to field constraints, this might overestimate the impact of some drivers (e.g., red deer or climate severity) on the survival of the reproductive—that is, adult—segment of the population, as part of it might be caused by variation in yearling survival. All these limitations warrant caution in the interpretation of our results, a deeper understanding of chamois population dynamics calls for the long‐term investigation of marked individuals in populations experiencing different levels of interspecific competition and climatic conditions.

## CONFLICT OF INTEREST

We have no competing interests.

## AUTHOR CONTRIBUTIONS

**Valerio Donini:** Conceptualization (equal); Data curation (lead); Formal analysis (lead); Methodology (equal); Writing‐original draft (lead); Writing‐review & editing (equal). **Luca Pedrotti:** Conceptualization (equal); Data curation (equal); Investigation (equal); Methodology (equal); Supervision (equal); Writing‐original draft (supporting); Writing‐review & editing (equal). **Francesco Ferretti:** Conceptualization (equal); Supervision (equal); Writing‐original draft (supporting); Writing‐review & editing (equal). **Luca Corlatti:** Conceptualization (equal); Data curation (equal); Formal analysis (lead); Investigation (equal); Methodology (equal); Supervision (lead); Writing‐original draft (lead); Writing‐review & editing (equal).

## Supporting information

Supplementary Material

## Data Availability

Data are available in the Supplementary file, along with the codes used for analysis.
