# Supplementary material for: Disentangling demographic effects of red deer on chamois population dynamics
Source: Ecol Evol. 2021 May 25;11(12):8264–80. doi: 10.1002/ece3.7657 (PMC8216891; doi:10.1002/ece3.7657)
Supplement: Supplementary file 1 — Supplementary Material [file ECE3-11-8264-s001.pdf]

DISENTANGLING DEMOGRAPHIC EFFECTS OF RED DEER ON CHAMOIS POPULATION DYNAMICS

Supplementary file

Valerio Donini, Luca Pedrotti, Francesco Ferretti, Luca Corlatti

Contents

1) LOAD & INSPECT DATA
- 1.1) Create dataset
- 1.2) Define variables

2) LOAD LIBRARIES
3) PRELIMINARY GRAPHS
4) PRELIMINARY DATA ANALYSIS
5) GLOBAL MODEL INSPECTION
- 5.1) Fit Bayesian global linear models
- 5.2) Inspect trace plots and posterior predictive checks
- 5.3) Inspect residuals of Bayesian global linear models
- 5.4) Check VIF for Bayesian global linear models

6) FIT MODELS FOR CHAMOIS DEMOGRAPHIC PARAMETERS
- 6.1) Formulate hypotheses (H1-H2-H3-H4-H5)
- 6.3) Define model structure for H1-H2-H3-H4
- 6.4) FIT BAYESIAN MODELS
  - 6.4.1) Modeling birth rate
  - 6.4.2) Modeling kid survival
  - 6.4.3) Modeling adult female survival
  - 6.4.4) Modeling adult male survival
- 6.5) FIT OLS MODELS
  - 6.5.1) Modeling birth rate
  - 6.5.2) Modeling kid survival
  - 6.5.3) Modeling adult female survival
  - 6.5.4) Modeling adult male survival

7) BAYESIAN MODEL SELECTION
- 7.1) Using WAIC
- 7.2) Using LOO-CV

8) OLS MODEL SELECTION
- 8.1) Using AICc
- 8.2) Using RMSE

9) BAYESIAN MODEL VALIDATION
10) OLS MODEL VALIDATION
11) COMPARING BAYESIAN & OLS MODEL RESULTS
12) OLS MODEL AVERAGING RESULTS
13) PLOT MARGINAL EFFECTS SIGNIFICANT PREDICTORS FROM OLS MODELS
14) PATH ANALYSIS

Data analysis

To facilitate the understanding and replication of the document, we create the vectors for the variables that will be used for the analysis.

```
#==============================================================================
# 1) LOAD DATA
#==============================================================================

#-----------------------------------------------------------------------------
# 1.1) Create dataset
#-----------------------------------------------------------------------------
rm(list=ls())


Year_N<-c(1993, 1994, 1995, 1996, 1997, 1998, 1999, 2000, 2001, 2002, 2003, 2004, 2005, 2006, 2007, 2008, 2009, 2010, 2011, 2012, 2013, 2014, 2015, 2016, 2017, 2018, 2019, 2020)
N_chamois <- c(1299, 1692, 1768, 2179, 2024, 2068, 1979, 1914, 1323, 1310, 1346, 1340, 1400, 1328, 1259, 1196, 1025, 992, 891, 902, 745, 812, 801, 856, 864, 800, 702, 757)
N_chamois_1 <- c(1352, 1299, 1692, 1768, 2179, 2024, 2068, 1979, 1914, 1323, 1310, 1346, 1340, 1400, 1328, 1259, 1196, 1025, 992, 891, 902, 745, 812, 801, 856, 864, 800, 702)
N_deer_1 <- c(330, 485, 646, 857, 986, 1117, 1185, 1417, 1445, 1065, 1349, 1567, 1624, 1624, 1504, 1857, 1915, 1283, 1061, 1140, 1289, 1309, 1191, 1237, 1484, 1738, 1024, 1179)
Y_chamois <- c(0.264, 0.044, 0.209, -0.074, 0.022, -0.044, -0.033, -0.369, -0.010, 0.027, -0.004, 0.044, -0.053, -0.053, -0.051, -0.154, -0.033, -0.107, 0.012, -0.191, 0.086, -0.014, 0.066, 0.009,
-0.077, -0.131, 0.075, NA)
Birth_rate_t <- c(0.791, 0.633, 0.670, 0.552, 0.505, 0.495, 0.515, 0.444, 0.510, 0.571, 0.564, 0.558, 0.548, 0.530, 0.514, 0.501, 0.520, 0.542, 0.596, 0.557, 0.646, 0.585, 0.632, 0.626, 0.551, 0.572,
0.588, 0.565)
Birth_rate_t_1 <- c(0.644, 0.791, 0.633, 0.670, 0.552, 0.505, 0.495, 0.515, 0.444, 0.510, 0.571, 0.564, 0.558, 0.548, 0.530, 0.514, 0.501, 0.520, 0.542, 0.596, 0.557, 0.646, 0.585, 0.632, 0.626, 0.551,
0.572, 0.588)
Kid_survival <- c(0.293, 0.399, 0.419, 0.402, 0.426, 0.373, 0.339, 0.308, 0.407, 0.424, 0.458, 0.462, 0.433, 0.432, 0.380, 0.362, 0.377, 0.364, 0.396, 0.351, 0.392, 0.372, 0.355, 0.339, 0.361, 0.354,
0.371, NA)
Kid_survival_1 <- c(0.284, 0.293, 0.399, 0.419, 0.402, 0.426, 0.373, 0.339, 0.308, 0.407, 0.424, 0.458, 0.462, 0.433, 0.432, 0.380, 0.362, 0.377, 0.364, 0.396, 0.351, 0.392, 0.372, 0.355, 0.339, 0.361,
0.354, 0.371)
Female_survival <- c(0.893, 0.771, 0.929, 0.754, 0.854, 0.715, 0.731, 0.554, 0.703, 0.738, 0.769, 0.733, 0.695, 0.753, 0.677, 0.684, 0.733, 0.637, 0.820, 0.608, 0.882, 0.723, 0.800, 0.788, 0.727,
0.663, 0.815, NA)
Female_survival_1 <- c(0.762, 0.893, 0.771, 0.929, 0.754, 0.854, 0.715, 0.731, 0.554, 0.703, 0.738, 0.769, 0.733, 0.695, 0.753, 0.677, 0.684, 0.733, 0.637, 0.820, 0.608, 0.882, 0.723, 0.800, 0.788, 0.7
27,0.663, 0.815)
Male_survival <- c(1.000, 0.787, 0.844, 0.685, 0.624, 0.673, 0.684, 0.661, 0.702, 0.770, 0.685, 0.758, 0.773, 0.672, 0.778, 0.709, 0.708, 0.751, 0.656, 0.736, 0.683, 0.773, 0.818, 0.730, 0.668, 0.675,
0.812, NA)
Male_survival_1 <- c(0.823, 1.000, 0.787, 0.844, 0.685, 0.624, 0.673, 0.684, 0.661, 0.702, 0.770, 0.685, 0.758, 0.773, 0.672, 0.778, 0.709, 0.708, 0.751, 0.656, 0.736, 0.683, 0.773, 0.818, 0.730,
0.668, 0.675, 0.812)
P_jan.mar <- c(163.0, 120.0, 64.0, 72.4, 48.4, 154.6, 93.8, 280.4, 115.6, 17.6, 128.0, 28.0, 116.6, 119.0, 130.2, 202.0, 142.6, 65.4, 93.2, 159.8, 414.6, 138.4, 197.8, 94.4, 159.0, 112.4, 116.6, NA)
P1_jan.mar <- c(42.6, 163.0, 120.0, 64.0, 72.4, 48.4, 154.6, 93.8, 280.4, 115.6, 17.6, 128.0, 28.0, 116.6, 119.0, 130.2, 202.0, 142.6, 65.4, 93.2, 159.8, 414.6, 138.4, 197.8, 94.4, 159.0, 112.4, 116.6)
P_apr.jul <- c(317.6, 380.8, 345.6, 294.6, 351.6, 363.6, 217.6, 312.2, 368.0, 435.6, 232.4, 232.6, 265.6, 222.0, 233.4, 521.0, 340.0, 362.2, 284.4, 444.6, 468.0, 424.0, 280.6, 398.0, 310.6, 437.6,
453.8, 330.2)
P1_apr.jul <- c(502.2, 317.6, 380.8, 345.6, 294.6, 351.6, 363.6, 217.6, 312.2, 368.0, 435.6, 232.4, 232.6, 265.6, 222.0, 233.4, 521.0, 340.0, 362.2, 284.4, 444.6, 468.0, 424.0, 280.6, 398.0, 310.6,
437.6, 453.8)


data_dem_chamois_deer <- as.data.frame(cbind(Year_N, N_chamois, N_chamois_1, N_deer_1, Y_chamois, Birth_rate_t, Birth_rate_t_1, Kid_survival, Kid_survival_1, Female_survival, Female_survival_1,
Male_survival, Male_survival_1, P_apr.jul, P1_apr.jul, P_jan.mar, P1_jan.mar)) # this dataset contains filtered values derived from state space models
```

```
#-------------------------------------------------------------------------------
# 1.2) Define variables
#-------------------------------------------------------------------------------



# Year_N = year t
# N_chamois = chamois population size at year t
# N_chamois_1 = chamois population size at year t-1
# N_deer_1 = deer population size at year t-1
# Y_chamois = chamois growth rate[t] = N_chamois[t+1] / N_chamois[t]
# Birth_rate_t = Kids[t] / Female_adult[t]

# Kid survival = proportion of kids that survive from year t to year t+1. Yearling[t+1] / kids[t]
# Female survival = proportion of females that survive from year t to year t+1. Female_adult[t+1] / [Female_adult(t) + 1/2(Yearling(t))]
# Male survival = proportion of male that survive from year t to year t+1. Male_adult[t+1] / [Male_adult(t) + 1/2(Yearling(t))]
# Birth_rate_t_1 = Kids[t-1] / Female_adult[t-1]
# Kid survival_1 = proportion of kids that survive from year t-1 to year t. Yearling[t] / kids[t-1]
# Female survival_1 = proportion of females that survive from year t-1 to year t. Female_adult[t] / [Female_adult(t-1) + 1/2(Yearling(t-1))]
# Male survival_1 = proportion of males that survive from year t-1 to year t. Male_adult[t] / [Male_adult(t-1) + 1/2(Yearling(t-1))]
# P_jan.mar = cumulated precipitation (in mm) between January and March of year t+1
# P1_jan.mar = cumulated precipitation (in mm) between January and March of year t
# P_apr.jul = cumulated precipitation (in mm) between April and July of year t
# P1_apr.jul = cumulated precipitation (in mm) between April and July of year t-1



#===============================================================================
# 2) LOAD LIBRARIES
#===============================================================================
```

First we load libraries we needed for the analysis

```
library(PerformanceAnalytics) # to inspect pairwise correlations
library(rstanarm) # to fit Bayesian models and rank them based on WAIC and LOO-CV
library(shinystan) # to inspect Bayesian model diagnostics
library(DHARMa) # to inspect model residual diagnostics and temporal correlation
library(car) # to inspect VIF
library(performance) # to calculate R2 and inspect temporal correlation
library(MuMIn) # to rank and average OLS models based on AICc
library(rms) # to rank models based on RMSE
library(dplyr) # to rearrange dataframes
library(hcci) # to run wild bootstrapping
library(parameters) # to inspect model results
library(visreg) # to plot marginal effects
library(robustbase) # to fit robust OLS models
library(lavaan) # to run path analysis
```

```
#===============================================================================
# 3) PRELIMINARY GRAPHS
#===============================================================================
par(mfrow=c(5,1), mar=c(4,5,1,2), oma = c(0, 0, 0, 0))

# Red deer population size
plot(data_dem_chamois_deer$Year_N, data_dem_chamois_deer$N_deer, col = "white", xaxt = 'n', yaxt = 'n', las = 1, ann = TRUE, ylab = "N_red deer [t]", xlab = NA)
axis(side = 2, at = seq(500, 2000, by = 250), las = 2, cex.axis = 0.75, lwd.ticks = 0.75)
axis(side = 1, at=seq(1993, 2020, by = 1), las = 1, cex.axis = 0.75, lwd.ticks = 0.75)
lines(data_dem_chamois_deer$Year_N, data_dem_chamois_deer$N_deer, col = "black", lwd = 1.5)
text(2020, 1775, "A", cex = 1, font = 2)

# Birth rate
plot(data_dem_chamois_deer$Year_N, data_dem_chamois_deer$Birth_rate_t, col = "white", xaxt = 'n', yaxt = 'n', las = 1, ann = TRUE, ylab = "Birth rate [t]", xlab = NA)
axis(side = 2, at = seq(0, 1, by = 0.05), las = 2, cex.axis = 0.75, lwd.ticks = 0.75)
axis(side = 1, at = seq(1993, 2020, by = 1), las = 1, cex.axis = 0.75, lwd.ticks = 0.75)
lines(data_dem_chamois_deer$Year_N, data_dem_chamois_deer$Birth_rate_t, col = "black", lwd = 1.5)
text(2020, 0.76, "B", cex = 1, font = 2)

# Kid survival
```

```r
plot(data_dem_chamois_deer$Year_N, data_dem_chamois_deer$Kid_survival, col = "white", xaxt = 'n', yaxt = 'n', las = 1,
ann = TRUE, ylab = "Kid survival [(t+1)/t]", xlab = NA)
axis(side = 2, at = seq(0, 1, by = 0.05), las = 2, cex.axis = 0.75, lwd.ticks = 0.75)
axis(side = 1, at = seq(1993, 2019, by = 1), las = 1, cex.axis = 0.75, lwd.ticks = 0.75)
lines(data_dem_chamois_deer$Year_N, data_dem_chamois_deer$Kid_survival, col = "black", lwd = 1.5)
text(2020, 0.45, "C", cex = 1, font = 2)

# Female survival
plot(data_dem_chamois_deer$Year_N, data_dem_chamois_deer$Female_survival, col = "white", xaxt = 'n', yaxt = 'n', las = 1,
ann = TRUE, ylab = "Female survival [(t+1)/t]", xlab = NA)
axis(side = 2, at = seq(0, 1, by = 0.05), las = 2, cex.axis = 0.75, lwd.ticks = 0.75)
axis(side = 1, at = seq(1993, 2019, by = 1), las = 1, cex.axis = 0.75, lwd.ticks = 0.75)
lines(data_dem_chamois_deer$Year_N, data_dem_chamois_deer$Female_survival, col = "black", lwd = 1.5)
text(2020, 0.9, "D", cex = 1, font = 2)

# Male survival
plot(data_dem_chamois_deer$Year_N, data_dem_chamois_deer$Male_survival, col = "white", xaxt = 'n', yaxt = 'n', las = 1,
ann = TRUE, ylab = "Male survival [(t+1)/t]", xlab = "Year")
axis(side = 2, at = seq(0, 1, by = 0.05), las = 2, cex.axis = 0.75, lwd.ticks = 0.75)
lines(data_dem_chamois_deer$Year_N, data_dem_chamois_deer$Male_survival, col="black",lwd=1.5)
axis(side = 1, at = seq(1993, 2019, by = 1), las = 1, cex.axis = 0.75, lwd.ticks = 0.75)
text(2020, 0.97, "E", cex = 1, font = 2)
```

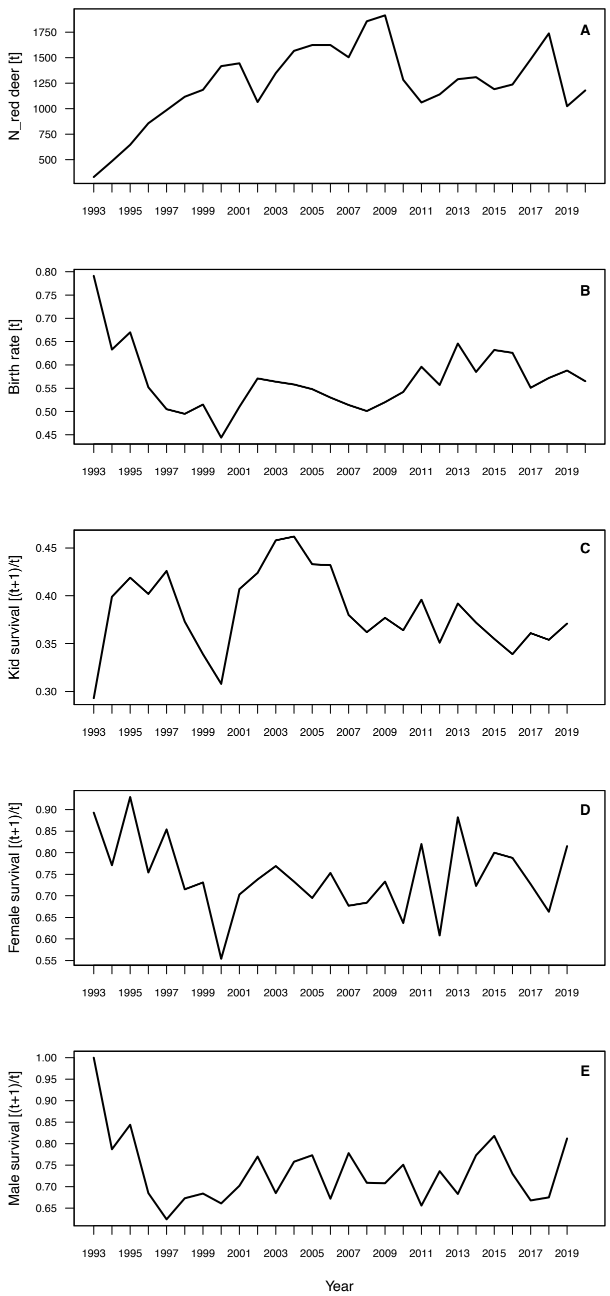

```r
#==============================================================================
# 4) PRELIMINARY DATA ANALYSIS
#==============================================================================

# Graphical check of pairwise correlations

PerformanceAnalytics::chart.Correlation(data_dem_chamois_deer)
```

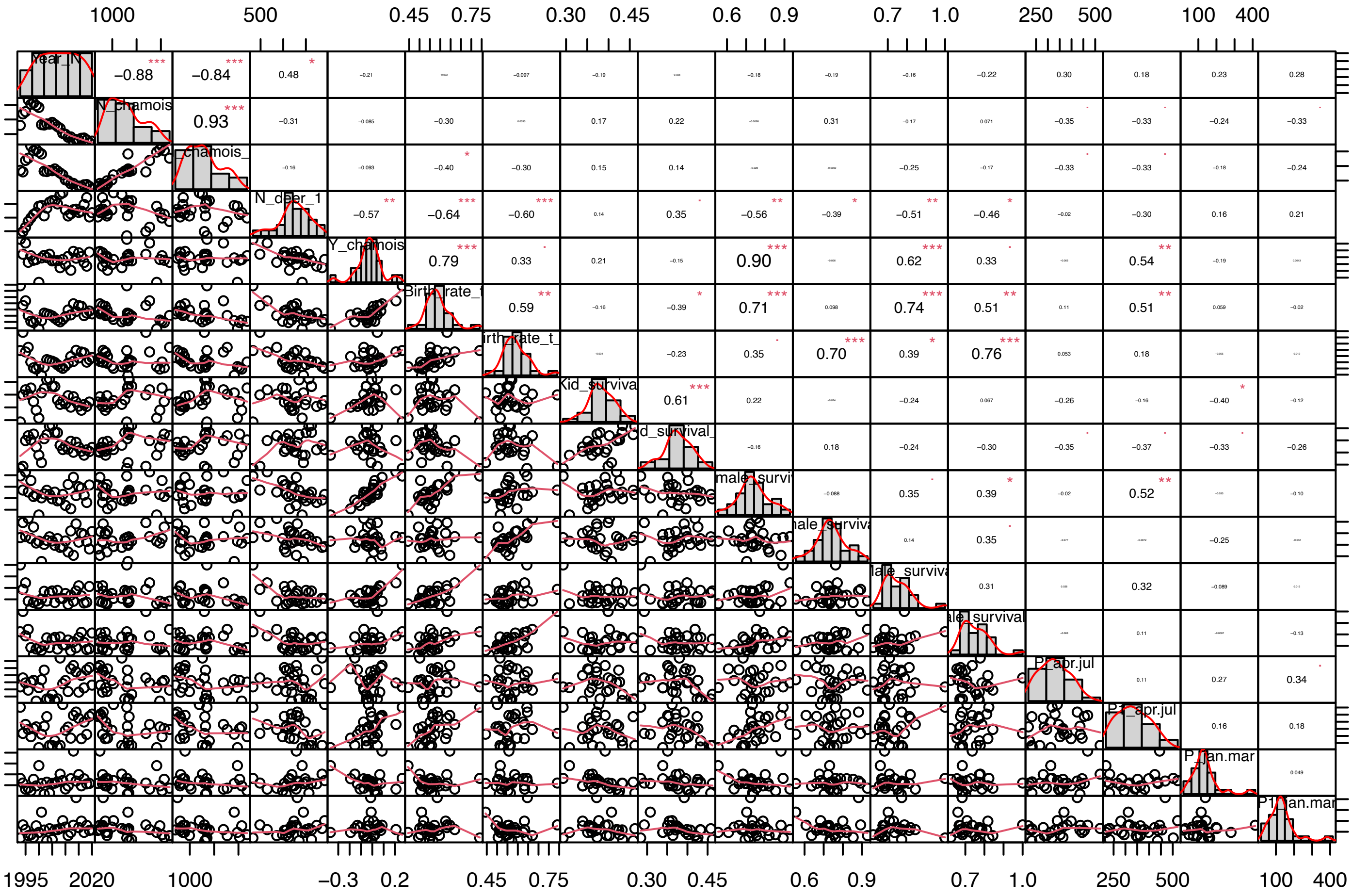

```
#==============================================================================
# 5) GLOBAL MODEL INSPECTION
#==============================================================================

#------------------------------------------------------------------------------
# 5.1) Fit Bayesian global linear models
#------------------------------------------------------------------------------
```

We fitted 4 different Bayesian global models, one for each parameter. The joint posterior distribution of regression coefficients was estimated via Markov Chain Monte Carlo (MCMC) with 20000 sampling iterations over 4 chains including 1000 warmups for each chains.

```
mod.global.1.br.stan <- stan_glm(Birth_rate_t ~ scale(Birth_rate_t_1) + (scale(P1_jan.mar) + scale(P1_apr.jul)) * (scale(N_chamois_1)+scale(N_deer_1)),
                        data = data_dem_chamois_deer, chains = 4, iter = 5000, warmup = 1000)
mod.global.2.ks.stan <- stan_glm(Kid_survival ~ scale(Kid_survival_1) + (scale(P_jan.mar) + scale(P_apr.jul)) * (scale(N_chamois)+scale(N_deer_1)),
                        data = data_dem_chamois_deer, chains = 4, iter = 5000, warmup = 1000)
mod.global.3.fs.stan <- stan_glm(Female_survival ~ scale(Female_survival_1) + (scale(P_jan.mar) + scale(P_apr.jul)) * (scale(N_chamois)+scale(N_deer_1)),
                        data = data_dem_chamois_deer, chains = 4, iter = 5000, warmup = 1000)
mod.global.4.ms.stan <- stan_glm(Male_survival ~ scale(Male_survival_1) + (scale(P_jan.mar) + scale(P_apr.jul)) * (scale(N_chamois)+scale(N_deer_1)),
                        data = data_dem_chamois_deer, chains = 4, iter = 5000, warmup = 1000)
```

```
#------------------------------------------------------------------------------
# 5.2) Inspect trace plots and posterior predictive checks
#------------------------------------------------------------------------------
```

We checked for convergenge issues. Trace plots shows the sampled values per chain while posterior predictive check simulate replicated data under a fitted model and and compare to the bserved data. Visual inspection did not suggest major violation of convergence fo all the 4 models.

```
# mod.global.1.br.stan
plot(mod.global.1.br.stan, "trace") # the trace plots are OK!
pp_check(mod.global.1.br.stan) # the posterior predictive check is OK!
launch_shinystan(mod.global.1.br.stan) # we can thoroughly check the fit ofthe model with Shinystan
```

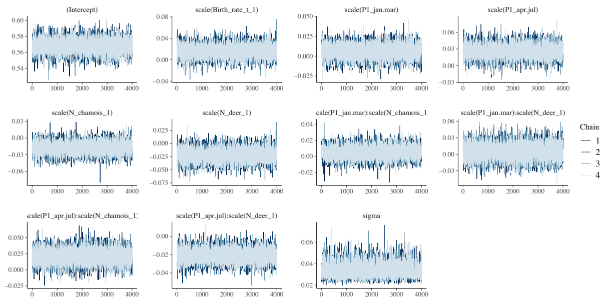

```
# mod.global.2.ks.stan
plot(mod.global.2.ks.stan, "trace") # the trace plots are OK!
pp_check(mod.global.2.ks.stan) # the posterior predictive check is OK!
launch_shinystan(mod.global.2.ks.stan) # we can thoroughly check the fit of the model with Shinystan
```

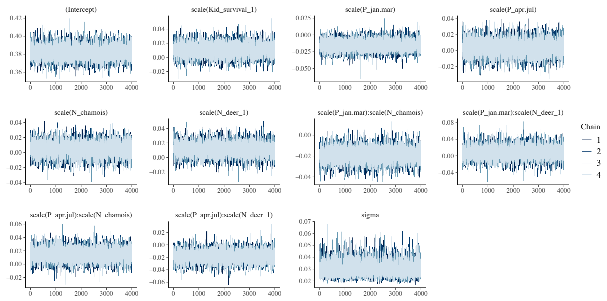

```
# mod.global.3.fs.stan
plot(mod.global.3.fs.stan, "trace") # the trace plots are OK!
pp_check(mod.global.3.fs.stan) # the posterior predictive check is OK!
launch_shinystan(mod.global.3.fs.stan) # we can thoroughly check the fit of the model with Shinystan
```

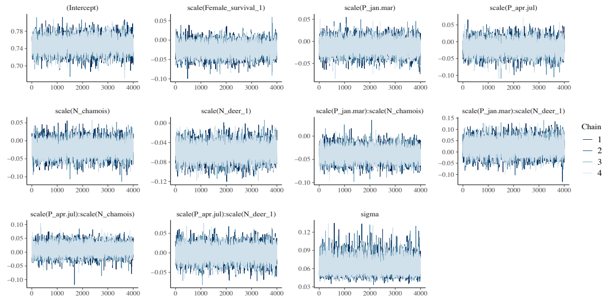


```
# mod.global.4.ms.stan
plot(mod.global.4.ms.stan, "trace") # the trace plots are OK!
pp_check(mod.global.4.ms.stan) # the posterior predictive check is OK!
launch_shinystan(mod.global.4.ms.stan) # we can thoroughly check the fit of the model with Shinystan
```

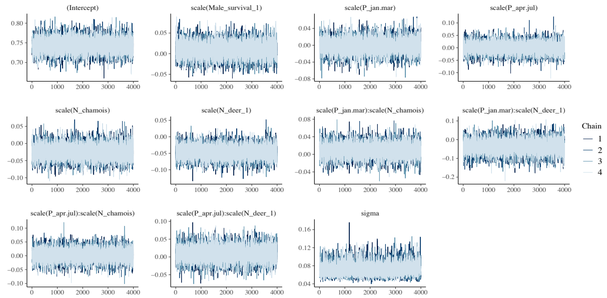


```
#-----------------------------------------------------------------------------
# 5.3) Inspect residuals of Bayesian global linear models
#-----------------------------------------------------------------------------

# mod.global.1.br.stan
par(mar=c(1,1,1,1))
predict.mod.global.1.br.stan <- t(posterior_predict(mod.global.1.br.stan)) # extract posterior predictions to feed to DHARMa
sim.resid.mod.global.1.br.stan <- createDHARMa(simulatedResponse = predict.mod.global.1.br.stan, observedResponse = data_dem_chamois_deer$Birth_rate_t)
plot(sim.resid.mod.global.1.br.stan) # the overall fit is OK!
par(mfrow = c(4,1))
plotResiduals(sim.resid.mod.global.1.br.stan, form = data_dem_chamois_deer$P1_jan.mar, xlab = "P1_jan.mar")
plotResiduals(sim.resid.mod.global.1.br.stan, form = data_dem_chamois_deer$P1_apr.jul, xlab = "P1_apr.jul")
plotResiduals(sim.resid.mod.global.1.br.stan, form = data_dem_chamois_deer$N_chamois_1, xlab = "N_chamois_1")
plotResiduals(sim.resid.mod.global.1.br.stan, form = data_dem_chamois_deer$N_deer_1, xlab = "N_deer_1")
testTemporalAutocorrelation(sim.resid.mod.global.1.br.stan, time = data_dem_chamois_deer$Year_N) # test temporal correlation
check_autocorrelation(mod.global.1.br.stan) # test autocorrelation
OK: Residuals appear to be independent and not autocorrelated (p = 0.298).
```

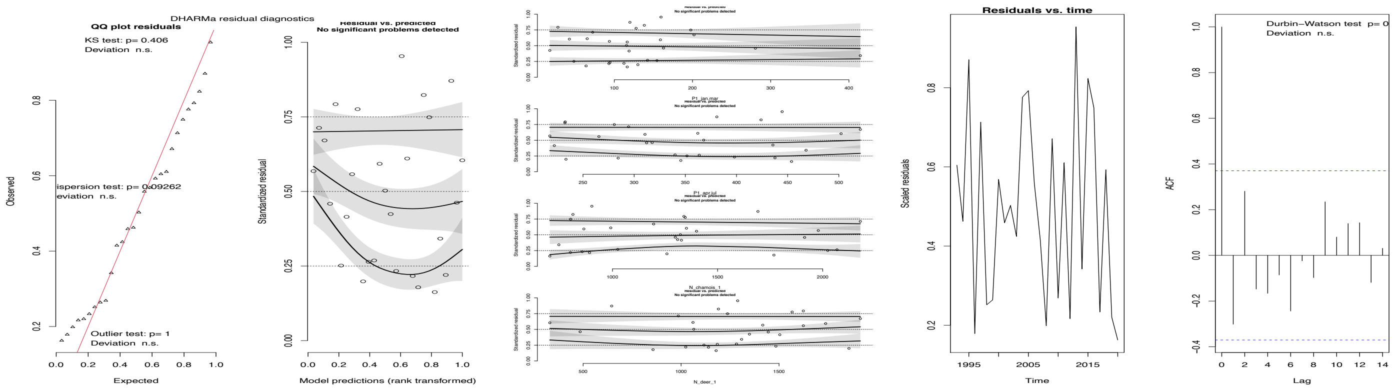


```
# mod.global.2.ks.stan
par(mar=c(1,1,1,1))
predict.mod.global.2.ks.stan  <- t(posterior_predict(mod.global.2.ks.stan)) # extract posterior predictions to feed to DHARMa
sim.resid.mod.global.2.ks.stan  <- createDHARMa(simulatedResponse = predict.mod.global.2.ks.stan , observedResponse=data_dem_chamois_deer$Kid_survival)
plot(sim.resid.mod.global.2.ks.stan) # the overall fit is OK!
par(mfrow=c(4,1))
plotResiduals(sim.resid.mod.global.2.ks.stan, form = data_dem_chamois_deer$P_jan.mar, xlab = "P_jan.mar")
plotResiduals(sim.resid.mod.global.2.ks.stan, form = data_dem_chamois_deer$P_apr.jul, xlab = "P_apr.jul")
plotResiduals(sim.resid.mod.global.2.ks.stan, form = data_dem_chamois_deer$N_chamois, xlab = "N_chamois")
plotResiduals(sim.resid.mod.global.2.ks.stan, form = data_dem_chamois_deer$N_deer_1, xlab = "N_deer_1")
testTemporalAutocorrelation(sim.resid.mod.global.2.ks.stan, time = time = (na.omit(data_dem_chamois_deer)$Year_N) # test temporal correlation
check_autocorrelation(mod.global.2.ks.stan) # test autocorrelation
OK: Residuals appear to be independent and not autocorrelated (p = 0.284).
```

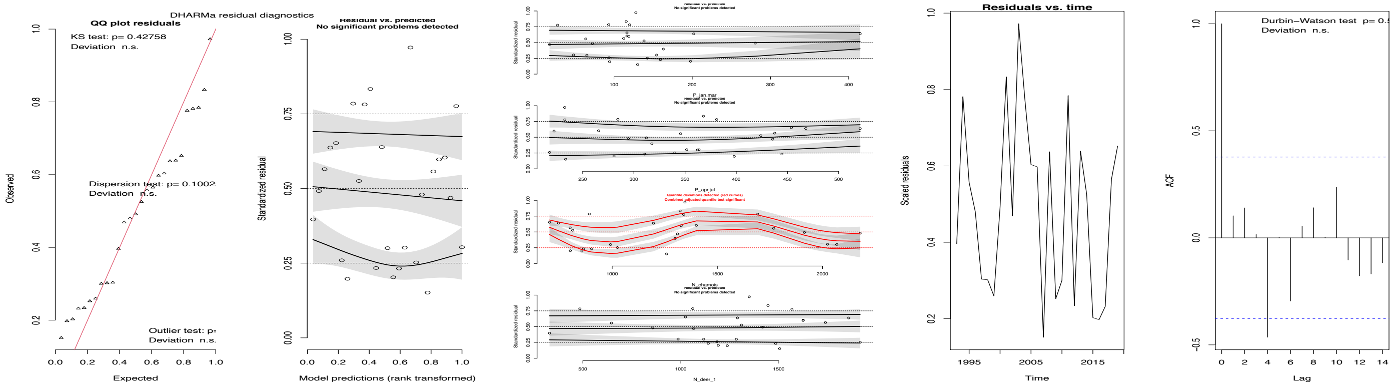


```
# mod.global.3.fs.stan
par(mar=c(1,1,1,1))
predict.mod.global.3.fs.stan  <- t(posterior_predict(mod.global.3.fs.stan)) # extract posterior predictions to feed to DHARMa
sim.resid.mod.global.3.fs.stan  <- createDHARMa(simulatedResponse = predict.mod.global.3.fs.stan , observedResponse=data_dem_chamois_deer$Female_survival)
plot(sim.resid.mod.global.3.fs.stan) # the overall fit is OK!
par(mfrow=c(4,1))
plotResiduals(sim.resid.mod.global.3.fs.stan, form = data_dem_chamois_deer$P_jan.mar, xlab = "P_jan.mar")
plotResiduals(sim.resid.mod.global.3.fs.stan, form = data_dem_chamois_deer$P_apr.jul, xlab = "P_apr.jul")
```

```
plotResiduals(sim.resid.mod.global.3.fs.stan, form = data_dem_chamois_deer$N_chamois, xlab = "N_chamois")
plotResiduals(sim.resid.mod.global.3.fs.stan, form = data_dem_chamois_deer$N_deer_1, xlab = "N_deer_1")
testTemporalAutocorrelation(sim.resid.mod.global.3.fs.stan, time = (na.omit(data_dem_chamois_deer)$Year_N) # test temporal correlation
check_autocorrelation(mod.global.3.fs.stan) # test autocorrelation
OK: Residuals appear to be independent and not autocorrelated (p = 0.596).
```

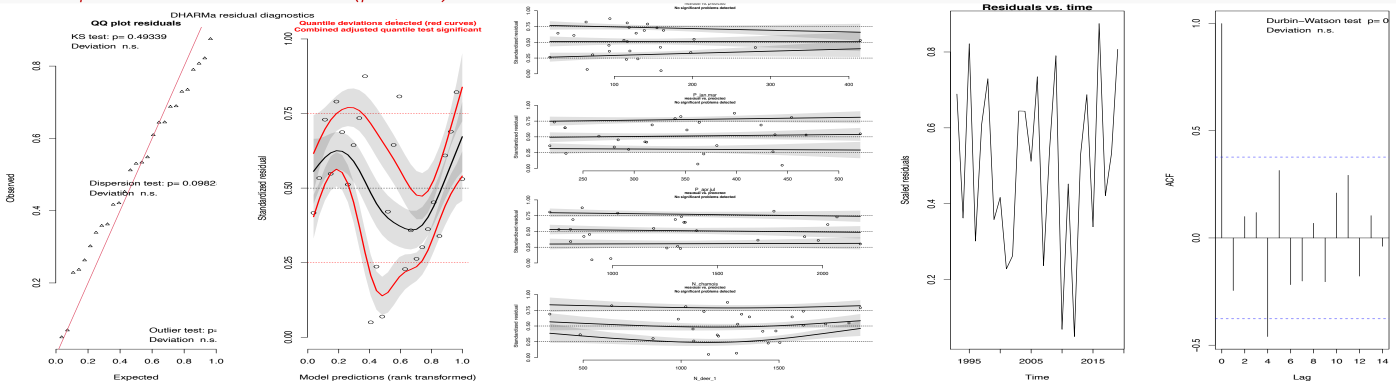


```
# mod.global.4.ms.stan
par(mar=c(1,1,1,1))
predict.mod.global.4.ms.stan  <- t(posterior_predict(mod.global.4.ms.stan)) # extract posterior predictions to feed to DHARMa
sim.resid.mod.global.4.ms.stan  <- createDHARMa(simulatedResponse = predict.mod.global.4.ms.stan , observedResponse=data_dem_chamois_deer$Male_survival)
plot(sim.resid.mod.global.4.ms.stan) # the overall fit is OK!
par(mfrow=c(4,1))
plotResiduals(sim.resid.mod.global.4.ms.stan, form = data_dem_chamois_deer$P_jan.mar, xlab = "P_jan.mar")
plotResiduals(sim.resid.mod.global.4.ms.stan, form = data_dem_chamois_deer$P_apr.jul, xlab = "P_apr.jul")
plotResiduals(sim.resid.mod.global.4.ms.stan, form = data_dem_chamois_deer$N_chamois, xlab = "N_chamois")
plotResiduals(sim.resid.mod.global.4.ms.stan, form = data_dem_chamois_deer$N_deer_1, xlab = "N_deer_1")
testTemporalAutocorrelation(sim.resid.mod.global.4.ms.stan, time = (na.omit(data_dem_chamois_deer)$Year_N) # test temporal correlation
check_autocorrelation(mod.global.4.ms.stan) # test autocorrelation
OK: Residuals appear to be independent and not autocorrelated (p = 0.306).
```

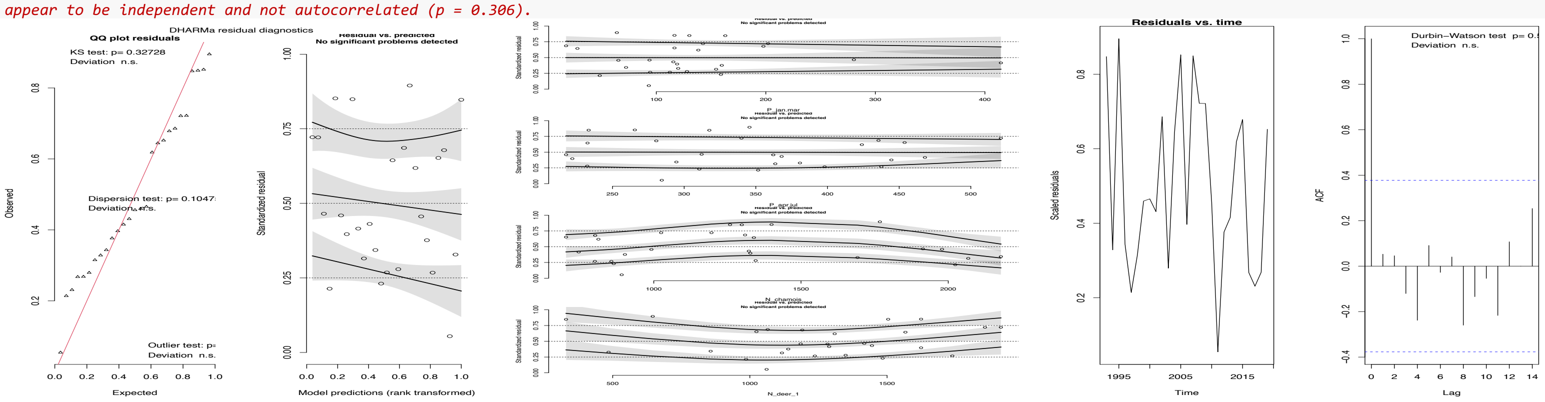

```r
#------------------------------------------------------------------------------
# 5.4) Check VIF for Bayesian global linear models
#------------------------------------------------------------------------------

vif(mod.global.1.br.stan) # > 3 for scale(N_deer_1)
```

scale(Birth_rate_t_1)	scale(P1_jan.mar)	scale(N_deer_1)	scale(P1_jan.mar):scale(N_chamois_1)	scale(P1_apr.jul):scale(N_deer_1)	scale(P1_apr.jul)	scale(N_chamois_1)	scale(P1_jan.mar):scale(N_deer_1)	scale(P1_apr.jul):scale(N_chamois_1)
2.780042	1.457517	3.090655	1.362028	1.592523	2.864391	2.170175	2.052198	1.285531

```r
# refit a model without scale(Birth_rate_t_1) and check parameters
mod.global.1.br.stan.check <- stan_glm(Birth_rate_t ~ (scale(P1_jan.mar) + scale(P1_apr.jul)) * (scale(N_chamois_1)+scale(N_deer_1)), data = data_dem_chamois_deer, chains = 4, iter = 5000, warmup = 100
0)
parameters(mod.global.1.br.stan)
Parameter                            |   Median |         89% CI |     pd | % in ROPE |  Rhat |      ESS |                 Prior
---------------------------------------------------------------------------------------------------------------------------------
(Intercept)                          |     0.57 | [ 0.56,  0.58] |   100% |        0% | 1.000 | 16867.48 | Normal (0.57 +- 0.17)
scale(Birth_rate_t_1)                |     0.02 | [ 0.00,  0.03] | 93.33% |    16.64% | 1.000 |  8011.26 | Normal (0.00 +- 0.17)
scale(P1_jan.mar)                    | 6.45e-03 | [-0.01,  0.02] | 79.83% |    47.08% | 1.000 | 12805.11 | Normal (0.00 +- 0.17)
scale(P1_apr.jul)                    |     0.03 | [ 0.01,  0.04] | 98.94% |     3.91% | 1.000 |  7190.35 | Normal (0.00 +- 0.17)
scale(N_chamois_1)                   |    -0.02 | [-0.03,  0.00] | 96.16% |    12.56% | 1.001 |  7685.52 | Normal (0.00 +- 0.17)
scale(N_deer_1)                      |    -0.02 | [-0.04,  0.00] | 96.94% |     8.24% | 1.000 |  7512.40 | Normal (0.00 +- 0.17)
scale(P1_jan.mar):scale(N_chamois_1) | 4.96e-03 | [-0.01,  0.02] | 76.72% |    56.07% | 1.000 | 12788.61 | Normal (0.00 +- 0.15)
scale(P1_jan.mar):scale(N_deer_1)    | 5.02e-03 | [-0.02,  0.03] | 65.21% |    37.99% | 1.000 |  9604.16 | Normal (0.00 +- 0.23)
scale(P1_apr.jul):scale(N_chamois_1) |     0.02 | [ 0.00,  0.04] | 97.85% |     6.93% | 1.000 | 12570.62 | Normal (0.00 +- 0.23)
scale(P1_apr.jul):scale(N_deer_1)    |    -0.02 | [-0.03, -0.01] | 99.74% |     2.21% | 1.000 |  9422.24 | Normal (0.00 +- 0.14)

parameters(mod.global.1.br.stan.check)
Parameter                            |   Median |         89% CI |     pd | % in ROPE |  Rhat |      ESS |                 Prior
---------------------------------------------------------------------------------------------------------------------------------
(Intercept)                          |     0.57 | [ 0.56,  0.58] |   100% |        0% | 1.000 | 17450.50 | Normal (0.57 +- 0.17)
scale(P1_jan.mar)                    | 6.95e-03 | [-0.01,  0.02] | 81.04% |    44.04% | 1.000 | 13244.99 | Normal (0.00 +- 0.17)
scale(P1_apr.jul)                    |     0.02 | [ 0.00,  0.04] | 98.01% |     6.38% | 1.000 |  8081.52 | Normal (0.00 +- 0.17)
scale(N_chamois_1)                   |    -0.02 | [-0.04, -0.01] | 99.67% |     2.43% | 1.000 |  9303.46 | Normal (0.00 +- 0.17)
scale(N_deer_1)                      |    -0.03 | [-0.05, -0.02] | 99.98% |     0.08% | 1.000 | 11744.85 | Normal (0.00 +- 0.17)
scale(P1_jan.mar):scale(N_chamois_1) | 1.03e-03 | [-0.01,  0.01] | 56.19% |    69.52% | 1.000 | 16461.09 | Normal (0.00 +- 0.15)
scale(P1_jan.mar):scale(N_deer_1)    | 1.69e-03 | [-0.02,  0.02] | 55.36% |    40.09% | 1.000 |  9556.18 | Normal (0.00 +- 0.23)
scale(P1_apr.jul):scale(N_chamois_1) |     0.02 | [ 0.01,  0.04] | 98.46% |     5.70% | 1.000 | 12933.92 | Normal (0.00 +- 0.23)
scale(P1_apr.jul):scale(N_deer_1)    |    -0.02 | [-0.03, -0.01] | 99.52% |     3.49% | 1.000 | 10407.79 | Normal (0.00 +- 0.14)
# the two models return similar estimates for all parameters, declare collinearity inconsequential

vif(mod.global.2.ks.stan) # < 3
```

scale(Kid_survival_1)	scale(P_jan.mar)	scale(N_deer_1)	scale(P_jan.mar):scale(N_chamois)	scale(P_apr.jul):scale(N_deer_1)	scale(P_apr.jul)	scale(N_chamois)	scale(P_jan.mar):scale(N_deer_1)	scale(P_apr.jul):scale(N_chamois)
2.030340	1.478837	1.980677	1.365286	1.630079	2.159733	2.024005	1.641551	1.202040

```r
vif(mod.global.3.fs.stan) # < 3
```

scale(Female_survival_1)	scale(P_jan.mar)	scale(N_deer_1)	scale(P_jan.mar):scale(N_chamois)	scale(P_apr.jul):scale(N_deer_1)
1.354923	1.265200	1.406601	1.221822	1.470622

scale(P_apr.jul)	scale(N_chamois)	scale(P_jan.mar):scale(N_deer_1)	scale(P_apr.jul):scale(N_chamois)
2.190987	2.110221	1.741819	1.207156

```
vif(mod.global.4.ms.stan) # < 3
```

scale(Male_survival_1)	scale(P_jan.mar)	scale(N_deer_1)	scale(P_jan.mar):scale(N_chamois)	scale(P_apr.jul):scale(N_deer_1)
1.472157	1.232917	1.714462	1.219828	1.502615

scale(P_apr.jul)	scale(N_chamois)	scale(P_jan.mar):scale(N_deer_1)	scale(P_apr.jul):scale(N_chamois)
2.164731	1.954360	1.771227	1.264617

```
#==============================================================================
# 6) FIT MODELS FOR CHAMOIS GROWTH RATE PARAMETERS
#==============================================================================
```

```
#------------------------------------------------------------------------------
# 6.1) Formulate hypotheses (H1-H2-H3-H4-H5)
#------------------------------------------------------------------------------
```

In Corlatti et al. (2019), chamois growth rate was calculated excluding kids, therefore we expect it to be affected by birth rate at year [t], kid survival between year [t] and [t+1] (thereby allowing for yearling recruitment), adult female and male survival between year [t] and [t+1]. Corlatti et al. (2019) found that chamois growth rate between year [t] and [t+1] was affected by red deer abundance at year [t-1]. In principle, each one of these parameters may thus be influenced by red deer abundance with 1-year time lag. Chamois growth rate, however, was not only negatively affected by deer abundance, but also by the synergistic effect of winter precipitation at [t+1] and chamois abundance at [t] (Corlatti et al., 2019). Starting from these findings, in this study we aim to assess the non-mutually exclusive hypotheses that, in chamois: *H1*) red deer abundance at [t-1] negatively affect birth rate at time [t]; red deer abundance at [t-1] and the interaction of winter precipitation at [t+1] and chamois abundance at [t] negatively affect *H2*) kid survival, *H3*) adult female survival and *H4*) adult male survival between time [t] and [t+1]. The hypothesized effects are shown in Fig. 1. We also aim to test if the red deer negative effect on chamois growth rate is mediated by birth rate, kid survival, adult female survival or adult male survival (*H5*).

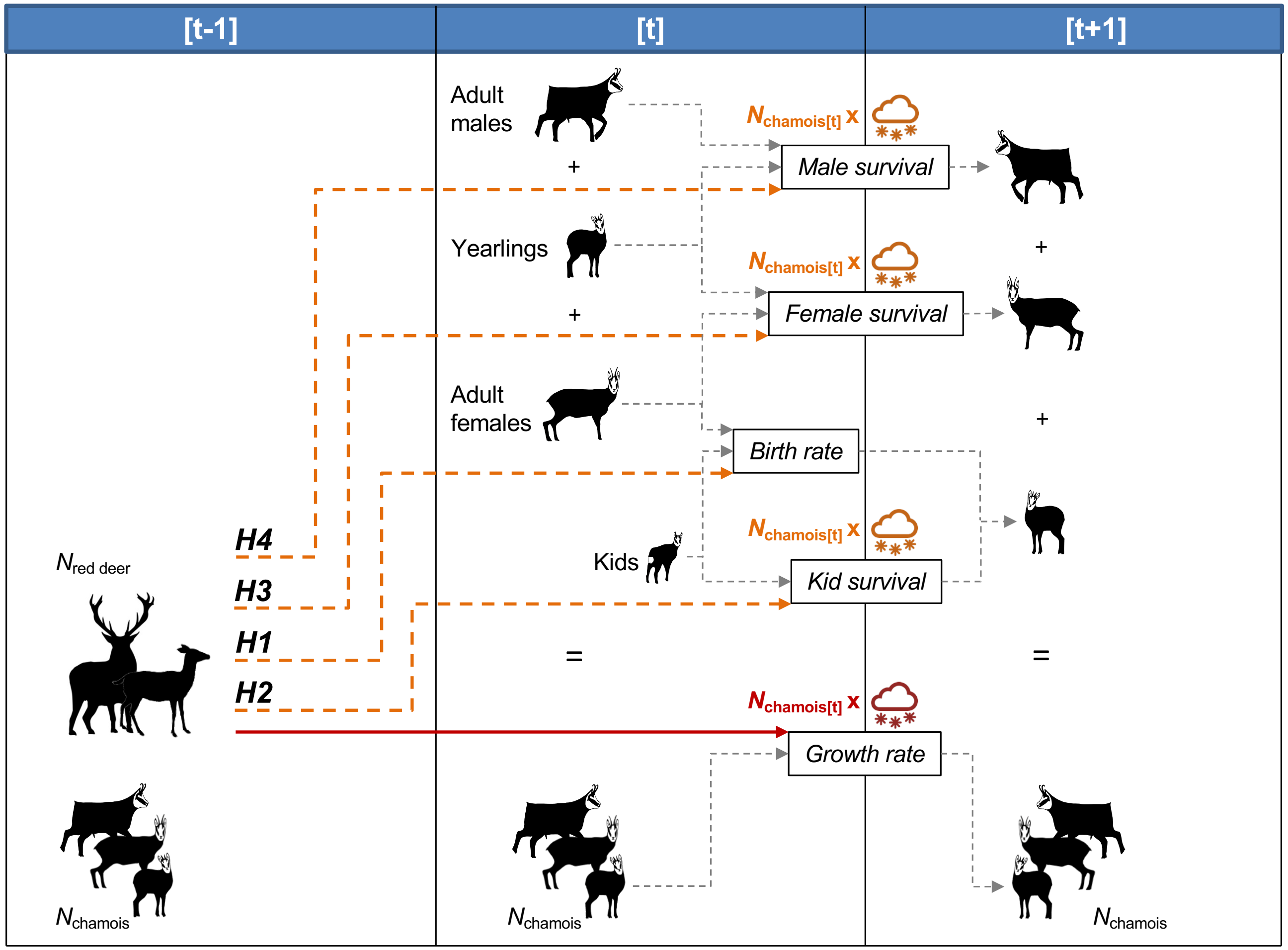

Figure 2. Scheme of the hypothetical patterns tested in this study to explain variation in demographic parameters of Alpine chamois in the Stelvio National Park. Demographic parameters are reported within rectangles (see text for details). Grey dashed arrows indicate the contribution of each sex- and age class to different parameters. Red solid line and letters indicate the known negative relationship of red deer at [t-1] and of the interactive effects between chamois abundance at [t] and winter weather conditions at [t+1] with chamois growth rate between [t] and [t+1]. Orange dashed line represents the hypothesized negative effects of red deer abundance at [t-1], while orange symbols and letters indicate the hypothesized negative effects of winter weather conditions at [t] or [t+1] in synergy with chamois abundance at time [t-1] or [t] on: birth rate at time [t] (*H*1); kid survival between [t] and [t+1] (*H*2); adult females survival between [t] and [t+1] (*H*3); adult male survival between [t] and [t+1] (*H*4). Drawings by Luca Corlatti.

Here we present the full list of models (19 for each demographic parameter) and the biological meaning associated to each model.

```
#-----------------------------------------------------------------------------
# 6.3) FIT BAYESIAN MODELS
#-----------------------------------------------------------------------------
# all models include an autoregressive term to account for temporal correlation issues detected in preliminary analyses. IN BOLD CORRESPONDING MODELS TO H1,H2,H3,H4

# For birth rate [t]
mod.1.br: "Birth_rate_t ~ scale(P1_jan.mar) * scale(N_chamois_1) + scale(N_deer_1)" # medium term interactive effect of winter precipitation and chamois density on females condition and
                                                                                    # medium term effect of red deer abundance on females condition.
mod.2.br: "Birth_rate_t ~ scale(P1_jan.mar) * scale(N_deer_1) + scale(N_chamois_1)" # medium term interactive effect of winter precipitation and red deer abundance on females condition and
                                                                                    # medium term effect of chamois density on females condition.
mod.3.br: "Birth_rate_t ~ scale(P1_apr.jul) * scale(N_chamois_1) + scale(N_deer_1) "# medium term interactive effect of spring-summer precipitation and chamois density on females condition and
                                                                                    # medium term effect of red deer abundance on females condition.
mod.4.br: "Birth_rate_t ~ scale(P1_apr.jul) * scale(N_deer_1) + scale(N_chamois_1)" # medium term interactive effect of spring-summer precipitation and red deer abundance on female condition and
                                                                                    # medium term effect of chamois density on female condition.
mod.5.br: "Birth_rate_t ~ scale(P1_jan.mar) * scale(N_deer_1)" # medium term interactive effect of winter precipitation and red deer abundance on females condition.
mod.6.br: "Birth_rate_t ~ scale(P1_apr.jul) * scale(N_deer_1)" # medium term interactive effect of spring-summer precipitation and red deer abundance on females condition.
mod.7.br: "Birth_rate_t ~ scale(P1_jan.mar) + scale(N_chamois_1) + scale(N_deer_1)" # medium term addictive effect of winter precipitation, chamois density and red deer abundance on females condition.
mod.8.br: "Birth_rate_t ~ scale(P1_apr.jul) + scale(N_deer_1) + scale(N_chamois_1)" # medium term addictive effect of spring-summer precipitation, chamois density and red deer abundance on f. condition
mod.9.br: "Birth_rate_t ~ scale(P1_jan.mar) + scale(N_deer_1)" # medium term addictive effect of winter precipitation and red deer abundance on females condition.
mod.10.br: "Birth_rate_t ~ scale(P1_apr.jul) + scale(N_deer_1)" # medium term addictive effect of spring-summer precipitation and red deer abundance on females condition.
mod.11.br: "Birth_rate_t ~ scale(N_deer_1) + scale(N_chamois_1)" # medium term effect of chamois density and medium term effect of red deer abundance on females condition.
mod.12.br: "Birth_rate_t ~ scale(N_deer_1)" # medium term effect of red deer abundance on female condition.
mod.13.br: "Birth_rate_t ~ scale(P1_jan.mar) * scale(N_chamois_1)" # medium term interactive effect of winter precipitation and chamois density on females condition.
mod.14.br: "Birth_rate_t ~ scale(P1_apr.jul) * scale(N_chamois_1)" # medium term interactive effect of spring-summer precipitation and chamois density on females condition.
mod.15.br: "Birth_rate_t ~ scale(P1_jan.mar) + scale(N_chamois_1)" # medium term addictive effect of winter precipitation and chamois density on females condition.
mod.16.br: "Birth_rate_t ~ scale(P1_apr.jul) + scale(N_chamois_1)" # medium term addictive effect of spring-summer precipitation and chamois density on females condition.
mod.17.br: "Birth_rate_t ~ scale(P1_jan.mar)" # medium term effect of winter precipitation on females condition.
mod.18.br: "Birth_rate_t ~ scale(P1_apr.jul)" # medium term effect of spring-summer precipitation on females condition.
mod.19.br: "Birth_rate_t ~ scale(N_chamois_1)" # medium term effect of chamois density on females condition.

# For kid survival [(t+1) / t]
mod.1.ks: "Kid_survival ~ scale(P_jan.mar) * scale(N_chamois) + scale(N_deer_1)" # interaction between short term effect of winter precipitation and medium term effect of chamois density on kids
                                                                                  # condition and long term effect of red deer abundance on females condition.
mod.2.ks: "Kid_survival ~ scale(P_jan.mar) * scale(N_deer_1) + scale(N_chamois)" # interaction between short term effect of winter precipitation and long term effect of red deer abundance on
                                                                                  # females condition and medium term effect of chamois density on females condition.
mod.3.ks: "Kid_survival ~ scale(P_apr.jul) * scale(N_chamois) + scale(N_deer_1)" # interaction between medium term effect of spring-summer precipitation and medium term effect of chamois density
                                                                                  # on kids condition and long term effect of red deer on females condition.
mod.4.ks: "Kid_survival ~ scale(P_apr.jul) * scale(N_deer_1) + scale(N_chamois)" # interaction between medium term effect of  spring-summer precipitation and long term effect of red deer
                                                                                  # abundance on females condition and medium term effect of chamois density on kids condition.
mod.5.ks: "Kid_survival ~ scale(P_jan.mar) * scale(N_deer_1)" # interaction between short term effect of winter precipitation and long term effect of red deer abundance on females condition.
mod.6.ks: "Kid_survival ~ scale(P_apr.jul) * scale(N_deer_1)" # interaction between medium term effect of spring-summer precipitation and long term effect of red deer abundance on females condition.
mod.7.ks: "Kid_survival ~ scale(P_jan.mar) + scale(N_chamois) + scale(N_deer_1)" # short term effect of winter precipitation and medium term effect of chamois density on kids condition and long
                                                                                  # term effect of red deer abundance on females condition.
mod.8.ks: "Kid_survival ~ scale(P_apr.jul) + scale(N_deer_1) + scale(N_chamois)" # medium term effect of spring-summer precipitation and medium term effect of chamois density on kids condition
                                                                                  # and long term effect of red deer abundance on females condition.
mod.9.ks: "Kid_survival ~ scale(P_jan.mar) + scale(N_deer_1) "# short term effect of winter precipitation on kids condition and long term effect of red deer abundance on females condition.
mod.10.ks: "Kid_survival ~ scale(P_apr.jul) + scale(N_deer_1)" # medium term effect of winter precipitation on kids/females condition and long term effect of red deer abundance on females condition.
mod.11.ks: "Kid_survival ~ scale(N_deer_1) + scale(N_chamois)" # medium term effect of chamois density and long term effect of red deer abundance on females condition.
mod.12.ks: "Kid_survival ~ scale(N_deer_1)" # long term effect of red deer abundance on females condition.
mod.13.ks: "Kid_survival ~ scale(P_jan.mar) * scale(N_chamois)" # interaction between short term effect of winter precipitation and medium term effect of chamois density on kids condition.
mod.14.ks: "Kid_survival ~ scale(P_apr.jul) * scale(N_chamois)" # interaction between short term effect of spring-summer precipitation and medium term effect of chamois density on kids/fem. condition.
mod.15.ks: "Kid_survival ~ scale(P_jan.mar) + scale(N_chamois)" # short term effect of winter precipitation on kids condition and medium term effect of chamois density on kids/females condition.
mod.16.ks: "Kid_survival ~ scale(P_apr.jul) + scale(N_chamois)" # medium term effect of spring-summer precipitation on kids condition and medium term effect of chamois density on kids/females condition
mod.17.ks: "Kid_survival ~ scale(P_jan.mar)" # short term effect of winter precipitation on kids condition.
mod.18.ks: "Kid_survival ~ scale(P_apr.jul)" # short term effect of spring-summer precipitation on kids/females condition.
mod.19.ks: "Kid_survival ~ scale(N_chamois)" # medium term effect of chamois density on kids/females condition.

# For adult female survival [(t+1) / t]
mod.1.fs: "Female_survival ~ scale(P_jan.mar) * scale(N_chamois) + scale(N_deer_1)" # short term interactive effect of winter precipitation and chamois density and long term effect of red deer
                                                                                     # abundance on females condition.
mod.2.fs: "Female_survival ~ scale(P_jan.mar) * scale(N_deer_1) + scale(N_chamois)" # short term interactive effect of winter precipitation and long term effect of red deer abundance on females
                                                                                     # condition and short term effect of chamois density on females condition.
mod.3.fs: "Female_survival ~ scale(P_apr.jul) * scale(N_chamois) + scale(N_deer_1)" # medium term interactive effect of spring-summer precipitation and chamois density and long term effect of
                                                                                     # red deer abundance on females condition.
mod.4.fs: "Female_survival ~ scale(P_apr.jul) * scale(N_deer_1) + scale(N_chamois)" # interaction between medium-term spring-summer precipitation and long term effect of red deer abundance on
                                                                                     # females condition and medium term effect of chamois density on females condition.
mod.5.fs: "Female_survival ~ scale(P_jan.mar) * scale(N_deer_1)" # interaction between short term winter precipitation and long-term effect of red deer abundance on females condition.
```

```
mod.6.fs: "Female_survival ~ scale(P_apr.jul) * scale(N_deer_1)" # interaction between medium term spring-summer precipitation and long term effect of deer abundance on females condition.
mod.7.fs: "Female_survival ~ scale(P_jan.mar) + scale(N_chamois) + scale(N_deer_1)" # short term effect of winter precipitation, medium term effect of chamois density and long term effect of red
                                                                                      # deer abundance on females condition.
mod.8.fs: "Female_survival ~ scale(P_apr.jul) + scale(N_deer_1) + scale(N_chamois)" # medium term effect of spring-summer precipitation, medium term effect of chamois density and long term
                                                                                      # effect of red deer abundance on females condition.
mod.9.fs: "Female_survival ~ scale(P_jan.mar) + scale(N_deer_1)" # short term effect of winter precipitation and long term effect of red deer abundance on females condition.
mod.10.fs: "Female_survival ~ scale(P_apr.jul) + scale(N_deer_1)" # medium term effect of spring-summer precipitation and long term effect of red deer abundance on females condition.
mod.11.fs: "Female_survival ~ scale(N_deer_1) + scale(N_chamois)" # medium term effect of chamois density and long term effect of red deer abundance on females condition.
mod.12.fs: "Female_survival ~ scale(N_deer_1)" # long term effect of red deer abundance on females condition.
mod.13.fs: "Female_survival ~ scale(P_jan.mar) * scale(N_chamois)" # interaction between short term effect of winter precipitation and medium term effect of chamois density on females condition.
mod.14.fs: "Female_survival ~ scale(P_apr.jul) * scale(N_chamois)" # interaction between medium term effect of spring-summer precipitation and medium term effect of chamois density on females condition
mod.15.fs: "Female_survival ~ scale(P_jan.mar) + scale(N_chamois)" # short term effect of winter precipitation and medium term effect of chamois density on females condition.
mod.16.fs: "Female_survival ~ scale(P_apr.jul) + scale(N_chamois)" # medium term effect of spring-summer precipitation and medium term effect of chamois density on females condition.
mod.17.fs: "Female_survival ~ scale(P_jan.mar)" # short term effect of winter precipitation on females condition.
mod.18.fs: "Female_survival ~ scale(P_apr.jul)" # medium term effect of spring-summer precipitation on females condition.
mod.19.fs: "Female_survival ~ scale(N_chamois)" # medium term effect of chamois density on females condition.


# For adult male survival [(t+1) / t]
mod.1.ms: "Male_survival ~ scale(P_jan.mar) * scale(N_chamois) + scale(N_deer_1)" # short term interactive effect of winter precipitation and chamois density and long term effect of red deer
                                                                                   # abundance on males condition.
mod.2.ms: "Male_survival ~ scale(P_jan.mar) * scale(N_deer_1) + scale(N_chamois)" # interaction between short term effect of winter precipitation and long term effect of red deer abundance on
                                                                                   # males condition and short term effect of chamois density on males condition.
mod.3.ms: "Male_survival ~ scale(P_apr.jul) * scale(N_chamois) + scale(N_deer_1)" # medium term interactive effect of spring-summer precipitation and chamois density and long term effect of red
                                                                                   # deer abundance on males condition.
mod.4.ms: "Male_survival ~ scale(P_apr.jul) * scale(N_deer_1) + scale(N_chamois)" # interaction between medium term effect of spring-summer precipitation and long term effect of deer abundance
                                                                                   # on males condition and medium term effect of chamois density on males condition.
mod.5.ms: "Male_survival ~ scale(P_jan.mar) * scale(N_deer_1)" # interaction between short term effect of winter precipitation and long-term effect of red deer abundance on males condition.
mod.6.ms: "Male_survival ~ scale(P_apr.jul) * scale(N_deer_1)" # interaction between medium term effect of spring-summer precipitation and long-term effect of red deer abundance on males condition.
mod.7.ms: "Male_survival ~ scale(P_jan.mar) + scale(N_chamois) + scale(N_deer_1)" # short term effect of winter precipitation, medium term effect of chamois density and long term effect of red
                                                                                   # deer abundance on males condition.
mod.8.ms: "Male_survival ~ scale(P_apr.jul) + scale(N_deer_1) + scale(N_chamois)" # medium term effect of spring-summer precipitation, medium term effect of chamois density and long term effect
                                                                                   # of red deer abundance on males condition.
mod.9.ms: "Male_survival ~ scale(P_jan.mar) + scale(N_deer_1)" # short term effect of winter precipitation and long term effect of red deer abundance on males condition.
mod.10.ms: "Male_survival ~ scale(P_apr.jul) + scale(N_deer_1)" # medium term effect of spring-summer precipitation and long term effect of red deer abundance on males condition.
mod.11.ms: "Male_survival ~ scale(N_deer_1) + scale(N_chamois)" # medium term effect of chamois density and long term effect of red deer abundance on males condition.
mod.12.ms: "Male_survival ~ scale(N_deer_1)" # long term effect of red deer abundance on males condition.
mod.13.ms: "Male_survival ~ scale(P_jan.mar) * scale(N_chamois)" # interaction between short term effect of winter precipitation and medium term effect of chamois density on males condition.
mod.14.ms: "Male_survival ~ scale(P_apr.jul) * scale(N_chamois)" # interaction between medium term effect of spring-summer precipitation and medium term effect of chamois density on males condition.
mod.15.ms: "Male_survival ~ scale(P_jan.mar) + scale(N_chamois)" # short term effect of winter precipitation and medium term effect of chamois density on males condition.
mod.16.ms: "Male_survival ~ scale(P_apr.jul) + scale(N_chamois)" # medium term effect of spring-summer precipitation and medium term effect of chamois density on males condition.
mod.17.ms: "Male_survival ~ scale(P_jan.mar)" # medium term effect of winter precipitation on males condition.
mod.18.ms: "Male_survival ~ scale(P_apr.jul)" # medium term effect of spring-summer precipitation on males condition.
mod.19.ms: "Male_survival ~ scale(N_chamois)" # medium term effect of chamois density on males condition.


#++++++++++++++++++++++++++++++++++++++++++++++++++++++++++++++++++++++++++++++++++
# 6.3.1) Modeling birth rate
#++++++++++++++++++++++++++++++++++++++++++++++++++++++++++++++++++++++++++++++++++

# we start with models including red deer
mod.1.br.stan <- stan_glm(Birth_rate_t ~ scale(Birth_rate_t_1) + scale(P1_jan.mar) * scale(N_chamois_1) + scale(N_deer_1), data = data_dem_chamois_deer, chains = 4, iter = 5000, warmup=1000)
mod.2.br.stan <- stan_glm(Birth_rate_t ~ scale(Birth_rate_t_1) + scale(P1_jan.mar) * scale(N_deer_1) + scale(N_chamois_1), data = data_dem_chamois_deer, chains = 4, iter = 5000, warmup=1000)
mod.3.br.stan <- stan_glm(Birth_rate_t ~ scale(Birth_rate_t_1) + scale(P1_apr.jul) * scale(N_chamois_1) + scale(N_deer_1), data = data_dem_chamois_deer, chains = 4, iter = 5000, warmup=1000)
mod.4.br.stan <- stan_glm(Birth_rate_t ~ scale(Birth_rate_t_1) + scale(P1_apr.jul) * scale(N_deer_1) + scale(N_chamois_1), data = data_dem_chamois_deer, chains = 4, iter = 5000, warmup=1000)
mod.5.br.stan <- stan_glm(Birth_rate_t ~ scale(Birth_rate_t_1) + scale(P1_jan.mar) * scale(N_deer_1), data = data_dem_chamois_deer, chains = 4, iter = 5000, warmup=1000)
mod.6.br.stan <- stan_glm(Birth_rate_t ~ scale(Birth_rate_t_1) + scale(P1_apr.jul) * scale(N_deer_1), data = data_dem_chamois_deer, chains = 4, iter = 5000, warmup=1000)
mod.7.br.stan <- stan_glm(Birth_rate_t ~ scale(Birth_rate_t_1) + scale(P1_jan.mar) + scale(N_chamois_1) + scale(N_deer_1), data = data_dem_chamois_deer, chains = 4, iter = 5000, warmup=1000)
mod.8.br.stan <- stan_glm(Birth_rate_t ~ scale(Birth_rate_t_1) + scale(P1_apr.jul) + scale(N_deer_1) + scale(N_chamois_1), data = data_dem_chamois_deer, chains = 4, iter = 5000, warmup=1000)
mod.9.br.stan <- stan_glm(Birth_rate_t ~ scale(Birth_rate_t_1) + scale(P1_jan.mar) + scale(N_deer_1), data = data_dem_chamois_deer, chains = 4, iter = 5000, warmup=1000)
mod.10.br.stan <- stan_glm(Birth_rate_t ~ scale(Birth_rate_t_1) + scale(P1_apr.jul) + scale(N_deer_1), data = data_dem_chamois_deer, chains = 4, iter = 5000, warmup=1000)
mod.11.br.stan <- stan_glm(Birth_rate_t ~ scale(Birth_rate_t_1) + scale(N_chamois_1) + scale(N_deer_1), data = data_dem_chamois_deer, chains = 4, iter = 5000, warmup=1000)
mod.12.br.stan <- stan_glm(Birth_rate_t ~ scale(Birth_rate_t_1) + scale(N_deer_1) , data = data_dem_chamois_deer, chains = 4, iter = 5000, warmup=1000)
# then we fit models without red deer
mod.13.br.stan <- stan_glm(Birth_rate_t ~ scale(Birth_rate_t_1) + scale(P1_jan.mar) * scale(N_chamois_1), data = data_dem_chamois_deer, chains = 4, iter = 5000, warmup=1000)
mod.14.br.stan <- stan_glm(Birth_rate_t ~ scale(Birth_rate_t_1) + scale(P1_apr.jul) * scale(N_chamois_1), data = data_dem_chamois_deer, chains = 4, iter = 5000, warmup=1000)
mod.15.br.stan <- stan_glm(Birth_rate_t ~ scale(Birth_rate_t_1) + scale(P1_jan.mar) + scale(N_chamois_1), data = data_dem_chamois_deer, chains = 4, iter = 5000, warmup=1000)
mod.16.br.stan <- stan_glm(Birth_rate_t ~ scale(Birth_rate_t_1) + scale(P1_apr.jul) + scale(N_chamois_1), data = data_dem_chamois_deer, chains = 4, iter = 5000, warmup=1000)
mod.17.br.stan <- stan_glm(Birth_rate_t ~ scale(Birth_rate_t_1) + scale(P1_jan.mar), data = data_dem_chamois_deer, chains = 4, iter = 5000, warmup=1000)
```

```
mod.18.br.stan <- stan_glm(Birth_rate_t ~ scale(Birth_rate_t_1) + scale(P1_apr.jul), data = data_dem_chamois_deer, chains = 4, iter = 5000, warmup=1000)
mod.19.br.stan <- stan_glm(Birth_rate_t ~ scale(Birth_rate_t_1) + scale(N_chamois_1), data = data_dem_chamois_deer, chains = 4, iter = 5000, warmup=1000)
```

```
#++++++++++++++++++++++++++++++++++++++++++++++++++++++++++++++++++++++++++++++
# 6.3.2) Modeling kid survival
#++++++++++++++++++++++++++++++++++++++++++++++++++++++++++++++++++++++++++++++

# we start with models including red deer
mod.1.ks.stan <- stan_glm(Kid_survival ~ scale(Kid_survival_1) + scale(P_jan.mar) * scale(N_chamois) + scale(N_deer_1), data = data_dem_chamois_deer , chains = 4, iter = 5000, warmup=1000)
mod.2.ks.stan <- stan_glm(Kid_survival ~ scale(Kid_survival_1) + scale(P_jan.mar) * scale(N_deer_1) + scale(N_chamois), data = data_dem_chamois_deer, chains = 4, iter = 5000, warmup=1000)
mod.3.ks.stan <- stan_glm(Kid_survival ~ scale(Kid_survival_1) + scale(P_apr.jul) * scale(N_chamois) + scale(N_deer_1), data = data_dem_chamois_deer, chains = 4, iter = 5000, warmup=1000)
mod.4.ks.stan <- stan_glm(Kid_survival ~ scale(Kid_survival_1) + scale(P_apr.jul) * scale(N_deer_1) + scale(N_chamois), data = data_dem_chamois_deer, chains = 4, iter = 5000, warmup=1000)
mod.5.ks.stan <- stan_glm(Kid_survival ~ scale(Kid_survival_1) + scale(P_jan.mar) * scale(N_deer_1), data = data_dem_chamois_deer, chains = 4, iter = 5000, warmup=1000)
mod.6.ks.stan <- stan_glm(Kid_survival ~ scale(Kid_survival_1) + scale(P_apr.jul) * scale(N_deer_1), data = data_dem_chamois_deer, chains = 4, iter = 5000, warmup=1000)
mod.7.ks.stan <- stan_glm(Kid_survival ~ scale(Kid_survival_1) + scale(P_jan.mar) + scale(N_chamois) + scale(N_deer_1), data = data_dem_chamois_deer, chains = 4, iter = 5000, warmup=1000)
mod.8.ks.stan <- stan_glm(Kid_survival ~ scale(Kid_survival_1) + scale(P_apr.jul) + scale(N_deer_1) + scale(N_chamois), data = data_dem_chamois_deer, chains = 4, iter = 5000, warmup=1000)
mod.9.ks.stan <- stan_glm(Kid_survival ~ scale(Kid_survival_1) + scale(P_jan.mar) + scale(N_deer_1), data = data_dem_chamois_deer, chains = 4, iter = 5000, warmup=1000)
mod.10.ks.stan <- stan_glm(Kid_survival ~ scale(Kid_survival_1) + scale(P_apr.jul) + scale(N_deer_1), data = data_dem_chamois_deer, chains = 4, iter = 5000, warmup=1000)
mod.11.ks.stan <- stan_glm(Kid_survival ~ scale(Kid_survival_1) + scale(N_deer_1) + scale(N_chamois), data = data_dem_chamois_deer, chains = 4, iter = 5000, warmup=1000)
mod.12.ks.stan <- stan_glm(Kid_survival ~ scale(Kid_survival_1) + scale(N_deer_1), data = data_dem_chamois_deer, chains = 4, iter = 5000, warmup=1000)
# then we fit models without red deer
mod.13.ks.stan <- stan_glm(Kid_survival ~ scale(Kid_survival_1) + scale(P_jan.mar) * scale(N_chamois), data = data_dem_chamois_deer, chains = 4, iter = 5000, warmup=1000)
mod.14.ks.stan <- stan_glm(Kid_survival ~ scale(Kid_survival_1) + scale(P_apr.jul) * scale(N_chamois), data = data_dem_chamois_deer, chains = 4, iter = 5000, warmup=1000)
mod.15.ks.stan <- stan_glm(Kid_survival ~ scale(Kid_survival_1) + scale(P_jan.mar) + scale(N_chamois), data = data_dem_chamois_deer, chains = 4, iter = 5000, warmup=1000)
mod.16.ks.stan <- stan_glm(Kid_survival ~ scale(Kid_survival_1) + scale(P_apr.jul) + scale(N_chamois), data = data_dem_chamois_deer, chains = 4, iter = 5000, warmup=1000)
mod.17.ks.stan <- stan_glm(Kid_survival ~ scale(Kid_survival_1) + scale(P_jan.mar), data = data_dem_chamois_deer, chains = 4, iter = 5000, warmup=1000)
mod.18.ks.stan <- stan_glm(Kid_survival ~ scale(Kid_survival_1) + scale(P_apr.jul), data = data_dem_chamois_deer, chains = 4, iter = 5000, warmup=1000)
mod.19.ks.stan <- stan_glm(Kid_survival ~ scale(Kid_survival_1) + scale(N_chamois), data = data_dem_chamois_deer, chains = 4, iter = 5000, warmup=1000)
```

```
#++++++++++++++++++++++++++++++++++++++++++++++++++++++++++++++++++++++++++++++
# 6.3.3) Modeling adult female survival
#++++++++++++++++++++++++++++++++++++++++++++++++++++++++++++++++++++++++++++++

# we start with models including red deer
mod.1.fs.stan <- stan_glm(Female_survival ~ scale(Female_survival_1) + scale(P_jan.mar) * scale(N_chamois) + scale(N_deer_1), data = data_dem_chamois_deer, chains = 4, iter = 5000, warmup=1000)
mod.2.fs.stan <- stan_glm(Female_survival ~ scale(Female_survival_1) + scale(P_jan.mar) * scale(N_deer_1) + scale(N_chamois), data = data_dem_chamois_deer, chains = 4, iter = 5000, warmup=1000)
mod.3.fs.stan <- stan_glm(Female_survival ~ scale(Female_survival_1) + scale(P_apr.jul) * scale(N_chamois) + scale(N_deer_1), data = data_dem_chamois_deer, chains = 4, iter = 5000, warmup=1000)
mod.4.fs.stan <- stan_glm(Female_survival ~ scale(Female_survival_1) + scale(P_apr.jul) * scale(N_deer_1) +  scale(N_chamois), data = data_dem_chamois_deer, chains = 4, iter = 5000, warmup=1000)
mod.5.fs.stan <- stan_glm(Female_survival ~ scale(Female_survival_1) + scale(P_jan.mar) * scale(N_deer_1), data = data_dem_chamois_deer, chains = 4, iter = 5000, warmup=1000)
mod.6.fs.stan <- stan_glm(Female_survival ~ scale(Female_survival_1) + scale(P_apr.jul) * scale(N_deer_1), data = data_dem_chamois_deer, chains = 4, iter = 5000, warmup=1000)
mod.7.fs.stan <- stan_glm(Female_survival ~ scale(Female_survival_1) + scale(P_jan.mar) + scale(N_chamois) + scale(N_deer_1), data = data_dem_chamois_deer, chains = 4, iter = 5000, warmup=1000)
mod.8.fs.stan <- stan_glm(Female_survival ~ scale(Female_survival_1) + scale(P_apr.jul) + scale(N_deer_1) +  scale(N_chamois), data = data_dem_chamois_deer, chains = 4, iter = 5000, warmup=1000)
mod.9.fs.stan <- stan_glm(Female_survival ~ scale(Female_survival_1) + scale(P_jan.mar) + scale(N_deer_1), data = data_dem_chamois_deer, chains = 4, iter = 5000, warmup=1000)
mod.10.fs.stan <- stan_glm(Female_survival ~ scale(Female_survival_1) + scale(P_apr.jul) + scale(N_deer_1), data = data_dem_chamois_deer, chains = 4, iter = 5000, warmup=1000)
mod.11.fs.stan <- stan_glm(Female_survival ~ scale(Female_survival_1) + scale(N_deer_1) + scale(N_chamois), data = data_dem_chamois_deer, chains = 4, iter = 5000, warmup=1000)
mod.12.fs.stan <- stan_glm(Female_survival ~ scale(Female_survival_1) + scale(N_deer_1), data = data_dem_chamois_deer, chains = 4, iter = 5000, warmup=1000)
# then we fit models without red deer
mod.13.fs.stan <- stan_glm(Female_survival ~ scale(Female_survival_1) + scale(P_jan.mar) * scale(N_chamois), data = data_dem_chamois_deer, chains = 4, iter = 5000, warmup=1000)
mod.14.fs.stan <- stan_glm(Female_survival ~ scale(Female_survival_1) + scale(P_apr.jul) * scale(N_chamois), data = data_dem_chamois_deer, chains = 4, iter = 5000, warmup=1000)
mod.15.fs.stan <- stan_glm(Female_survival ~ scale(Female_survival_1) + scale(P_jan.mar) + scale(N_chamois), data = data_dem_chamois_deer, chains = 4, iter = 5000, warmup=1000)
mod.16.fs.stan <- stan_glm(Female_survival ~ scale(Female_survival_1) + scale(P_apr.jul) + scale(N_chamois), data = data_dem_chamois_deer, chains = 4, iter = 5000, warmup=1000)
mod.17.fs.stan <- stan_glm(Female_survival ~ scale(Female_survival_1) + scale(P_jan.mar), data = data_dem_chamois_deer, chains = 4, iter = 5000, warmup=1000)
mod.18.fs.stan <- stan_glm(Female_survival ~ scale(Female_survival_1) + scale(P_apr.jul), data = data_dem_chamois_deer, chains = 4, iter = 5000, warmup=1000)
mod.19.fs.stan <- stan_glm(Female_survival ~ scale(Female_survival_1) + scale(N_chamois), data = data_dem_chamois_deer, chains = 4, iter = 5000, warmup=1000)
```

```
#++++++++++++++++++++++++++++++++++++++++++++++++++++++++++++++++++++++++++++++
# 6.3.4) Modeling adult male survival
#++++++++++++++++++++++++++++++++++++++++++++++++++++++++++++++++++++++++++++++

# we start with models including red deer
mod.1.ms.stan <- stan_glm(Male_survival ~ scale(Male_survival_1) + scale(P_jan.mar) * scale(N_chamois) + scale(N_deer_1), data = data_dem_chamois_deer, chains = 4, iter = 5000, warmup=1000)
mod.2.ms.stan <- stan_glm(Male_survival ~ scale(Male_survival_1) + scale(P_jan.mar) * scale(N_deer_1) + scale(N_chamois), data = data_dem_chamois_deer, chains = 4, iter = 5000, warmup=1000)
mod.3.ms.stan <- stan_glm(Male_survival ~ scale(Male_survival_1) + scale(P_apr.jul) * scale(N_chamois) + scale(N_deer_1), data = data_dem_chamois_deer, chains = 4, iter = 5000, warmup=1000)
mod.4.ms.stan <- stan_glm(Male_survival ~ scale(Male_survival_1) + scale(P_apr.jul) * scale(N_deer_1) + scale(N_chamois), data = data_dem_chamois_deer, chains = 4, iter = 5000, warmup=1000)
mod.5.ms.stan <- stan_glm(Male_survival ~ scale(Male_survival_1) + scale(P_jan.mar) * scale(N_deer_1), data = data_dem_chamois_deer, chains = 4, iter = 5000, warmup=1000)
mod.6.ms.stan <- stan_glm(Male_survival ~ scale(Male_survival_1) + scale(P_apr.jul) * scale(N_deer_1), data = data_dem_chamois_deer, chains = 4, iter = 5000, warmup=1000)
mod.7.ms.stan <- stan_glm(Male_survival ~ scale(Male_survival_1) + scale(P_jan.mar) + scale(N_chamois) + scale(N_deer_1), data = data_dem_chamois_deer, chains = 4, iter = 5000, warmup=1000)
mod.8.ms.stan <- stan_glm(Male_survival ~ scale(Male_survival_1) + scale(P_apr.jul) + scale(N_deer_1) + scale(N_chamois), data = data_dem_chamois_deer, chains = 4, iter = 5000, warmup=1000)
mod.9.ms.stan <- stan_glm(Male_survival ~ scale(Male_survival_1) + scale(P_jan.mar) + scale(N_deer_1), data = data_dem_chamois_deer, chains = 4, iter = 5000, warmup=1000)
mod.10.ms.stan <- stan_glm(Male_survival ~ scale(Male_survival_1) + scale(P_apr.jul) + scale(N_deer_1), data = data_dem_chamois_deer, chains = 4, iter = 5000, warmup=1000)
```

```r
mod.11.ms.stan <- stan_glm(Male_survival ~ scale(Male_survival_1) + scale(N_deer_1) + scale(N_chamois), data = data_dem_chamois_deer, chains = 4, iter = 5000, warmup=1000)
mod.12.ms.stan <- stan_glm(Male_survival ~ scale(Male_survival_1) + scale(N_deer_1), data = data_dem_chamois_deer, chains = 4, iter = 5000, warmup=1000)
# then we fit models without red deer
mod.13.ms.stan <- stan_glm(Male_survival ~ scale(Male_survival_1) + scale(P_jan.mar) * scale(N_chamois), data = data_dem_chamois_deer, chains = 4, iter = 5000, warmup=1000)
mod.14.ms.stan <- stan_glm(Male_survival ~ scale(Male_survival_1) + scale(P_apr.jul) * scale(N_chamois), data = data_dem_chamois_deer, chains = 4, iter = 5000, warmup=1000)
mod.15.ms.stan <- stan_glm(Male_survival ~ scale(Male_survival_1) + scale(P_jan.mar) + scale(N_chamois), data = data_dem_chamois_deer, chains = 4, iter = 5000, warmup=1000)
mod.16.ms.stan <- stan_glm(Male_survival ~ scale(Male_survival_1) + scale(P_apr.jul) + scale(N_chamois), data = data_dem_chamois_deer, chains = 4, iter = 5000, warmup=1000)
mod.17.ms.stan <- stan_glm(Male_survival ~ scale(Male_survival_1) + scale(P_jan.mar), data = data_dem_chamois_deer, chains = 4, iter = 5000, warmup=1000)
mod.18.ms.stan <- stan_glm(Male_survival ~ scale(Male_survival_1) + scale(P_apr.jul), data = data_dem_chamois_deer, chains = 4, iter = 5000, warmup=1000)
mod.19.ms.stan <- stan_glm(Male_survival ~ scale(Male_survival_1) + scale(N_chamois), data = data_dem_chamois_deer, chains = 4, iter = 5000, warmup=1000)
```

```r
#-------------------------------------------------------------------------------
# 6.4) FIT OLS MODELS
#-------------------------------------------------------------------------------

# all models include an autoregressive term to account for temporal correlation issues detected in preliminary analyses

#+++++++++++++++++++++++++++++++++++++++++++++++++++++++++++++++++++++++++++++++
# 6.4.1) Modeling birth rate
#+++++++++++++++++++++++++++++++++++++++++++++++++++++++++++++++++++++++++++++++

# we start with models including red deer
mod.1.br.lm <- lm(Birth_rate_t ~ scale(Birth_rate_t_1) + scale(P1_jan.mar) * scale(N_chamois_1) + scale(N_deer_1), data = data_dem_chamois_deer)
mod.2.br.lm <- lm(Birth_rate_t ~ scale(Birth_rate_t_1) + scale(P1_jan.mar) * scale(N_deer_1) + scale(N_chamois_1), data = data_dem_chamois_deer)
mod.3.br.lm <- lm(Birth_rate_t ~ scale(Birth_rate_t_1) + scale(P1_apr.jul) * scale(N_chamois_1) + scale(N_deer_1), data = data_dem_chamois_deer)
mod.4.br.lm <- lm(Birth_rate_t ~ scale(Birth_rate_t_1) + scale(P1_apr.jul) * scale(N_deer_1) + scale(N_chamois_1), data = data_dem_chamois_deer)
mod.5.br.lm <- lm(Birth_rate_t ~ scale(Birth_rate_t_1) + scale(P1_jan.mar) * scale(N_deer_1), data = data_dem_chamois_deer)
mod.6.br.lm <- lm(Birth_rate_t ~ scale(Birth_rate_t_1) + scale(P1_apr.jul) * scale(N_deer_1), data = data_dem_chamois_deer)
mod.7.br.lm <- lm(Birth_rate_t ~ scale(Birth_rate_t_1) + scale(P1_jan.mar) + scale(N_chamois_1) + scale(N_deer_1), data = data_dem_chamois_deer)
mod.8.br.lm <- lm(Birth_rate_t ~ scale(Birth_rate_t_1) + scale(P1_apr.jul) + scale(N_deer_1) + scale(N_chamois_1), data = data_dem_chamois_deer)
mod.9.br.lm <- lm(Birth_rate_t ~ scale(Birth_rate_t_1) + scale(P1_jan.mar) + scale(N_deer_1), data = data_dem_chamois_deer)
mod.10.br.lm <- lm(Birth_rate_t ~ scale(Birth_rate_t_1) + scale(P1_apr.jul) + scale(N_deer_1), data = data_dem_chamois_deer)
mod.11.br.lm <- lm(Birth_rate_t ~ scale(Birth_rate_t_1) + scale(N_deer_1) + scale(N_chamois_1), data = data_dem_chamois_deer)
mod.12.br.lm <- lm(Birth_rate_t ~ scale(Birth_rate_t_1) + scale(N_deer_1), data = data_dem_chamois_deer)
# then we fit models without red deer
mod.13.br.lm <- lm(Birth_rate_t ~ scale(Birth_rate_t_1) + scale(P1_jan.mar) * scale(N_chamois_1), data = data_dem_chamois_deer)
mod.14.br.lm <- lm(Birth_rate_t ~ scale(Birth_rate_t_1) + scale(P1_apr.jul) * scale(N_chamois_1), data = data_dem_chamois_deer)
mod.15.br.lm <- lm(Birth_rate_t ~ scale(Birth_rate_t_1) + scale(P1_jan.mar) + scale(N_chamois_1), data = data_dem_chamois_deer)
mod.16.br.lm <- lm(Birth_rate_t ~ scale(Birth_rate_t_1) + scale(P1_apr.jul) + scale(N_chamois_1), data = data_dem_chamois_deer)
mod.17.br.lm <- lm(Birth_rate_t ~ scale(Birth_rate_t_1) + scale(P1_jan.mar), data = data_dem_chamois_deer)
mod.18.br.lm <- lm(Birth_rate_t ~ scale(Birth_rate_t_1) + scale(P1_apr.jul), data = data_dem_chamois_deer)
mod.19.br.lm <- lm(Birth_rate_t ~ scale(Birth_rate_t_1) + scale(N_chamois_1), data = data_dem_chamois_deer)
```

```r
#+++++++++++++++++++++++++++++++++++++++++++++++++++++++++++++++++++++++++++++++
# 6.4.2) Modeling kid survival
#+++++++++++++++++++++++++++++++++++++++++++++++++++++++++++++++++++++++++++++++

# we start with models including red deer
mod.1.ks.lm <- lm(Kid_survival ~ scale(Kid_survival_1) + scale(P_jan.mar) * scale(N_chamois) + scale(N_deer_1), data = data_dem_chamois_deer)
mod.2.ks.lm <- lm(Kid_survival ~ scale(Kid_survival_1) + scale(P_jan.mar) * scale(N_deer_1) + scale(N_chamois), data = data_dem_chamois_deer)
mod.3.ks.lm <- lm(Kid_survival ~ scale(Kid_survival_1) + scale(P_apr.jul) * scale(N_chamois) + scale(N_deer_1), data = data_dem_chamois_deer)
mod.4.ks.lm <- lm(Kid_survival ~ scale(Kid_survival_1) + scale(P_apr.jul) * scale(N_deer_1) + scale(N_chamois), data = data_dem_chamois_deer)
mod.5.ks.lm <- lm(Kid_survival ~ scale(Kid_survival_1) + scale(P_jan.mar) * scale(N_deer_1), data = data_dem_chamois_deer)
mod.6.ks.lm <- lm(Kid_survival ~ scale(Kid_survival_1) + scale(P_apr.jul) * scale(N_deer_1), data = data_dem_chamois_deer)
mod.7.ks.lm <- lm(Kid_survival ~ scale(Kid_survival_1) + scale(P_jan.mar) + scale(N_chamois) + scale(N_deer_1), data = data_dem_chamois_deer)
mod.8.ks.lm <- lm(Kid_survival ~ scale(Kid_survival_1) + scale(P_apr.jul) + scale(N_deer_1) + scale(N_chamois), data = data_dem_chamois_deer)
mod.9.ks.lm <- lm(Kid_survival ~ scale(Kid_survival_1) + scale(P_jan.mar) + scale(N_deer_1), data = data_dem_chamois_deer)
mod.10.ks.lm <- lm(Kid_survival ~ scale(Kid_survival_1) + scale(P_apr.jul) + scale(N_deer_1), data = data_dem_chamois_deer)
mod.11.ks.lm <- lm(Kid_survival ~ scale(Kid_survival_1) + scale(N_deer_1) + scale(N_chamois), data = data_dem_chamois_deer)
mod.12.ks.lm <- lm(Kid_survival ~ scale(Kid_survival_1) + scale(N_deer_1), data = data_dem_chamois_deer)
# then we fit models without red deer
mod.13.ks.lm <- lm(Kid_survival ~ scale(Kid_survival_1) + scale(P_jan.mar) * scale(N_chamois), data = data_dem_chamois_deer)
mod.14.ks.lm <- lm(Kid_survival ~ scale(Kid_survival_1) + scale(P_apr.jul) * scale(N_chamois), data = data_dem_chamois_deer)
mod.15.ks.lm <- lm(Kid_survival ~ scale(Kid_survival_1) + scale(P_jan.mar) + scale(N_chamois), data = data_dem_chamois_deer)
mod.16.ks.lm <- lm(Kid_survival ~ scale(Kid_survival_1) + scale(P_apr.jul) + scale(N_chamois), data = data_dem_chamois_deer)
mod.17.ks.lm <- lm(Kid_survival ~ scale(Kid_survival_1) + scale(P_jan.mar), data = data_dem_chamois_deer)
mod.18.ks.lm <- lm(Kid_survival ~ scale(Kid_survival_1) + scale(P_apr.jul), data = data_dem_chamois_deer)
mod.19.ks.lm <- lm(Kid_survival ~ scale(Kid_survival_1) + scale(N_chamois), data = data_dem_chamois_deer)
```

```r
#+++++++++++++++++++++++++++++++++++++++++++++++++++++++++++++++++++++++++++++++
# 6.4.3) Modeling adult female survival
#+++++++++++++++++++++++++++++++++++++++++++++++++++++++++++++++++++++++++++++++

# we start with models including red deer
mod.1.fs.lm <- lm(Female_survival ~ scale(Female_survival_1) + scale(P_jan.mar) * scale(N_chamois) + scale(N_deer_1), data = data_dem_chamois_deer)
mod.2.fs.lm <- lm(Female_survival ~ scale(Female_survival_1) + scale(P_jan.mar) * scale(N_deer_1) + scale(N_chamois), data = data_dem_chamois_deer)
mod.3.fs.lm <- lm(Female_survival ~ scale(Female_survival_1) + scale(P_apr.jul) * scale(N_chamois) + scale(N_deer_1), data = data_dem_chamois_deer)
mod.4.fs.lm <- lm(Female_survival ~ scale(Female_survival_1) + scale(P_apr.jul) * scale(N_deer_1) + scale(N_chamois), data = data_dem_chamois_deer)
mod.5.fs.lm <- lm(Female_survival ~ scale(Female_survival_1) + scale(P_jan.mar) * scale(N_deer_1), data = data_dem_chamois_deer)
mod.6.fs.lm <- lm(Female_survival ~ scale(Female_survival_1) + scale(P_apr.jul) * scale(N_deer_1), data = data_dem_chamois_deer)
mod.7.fs.lm <- lm(Female_survival ~ scale(Female_survival_1) + scale(P_jan.mar) + scale(N_chamois) + scale(N_deer_1), data = data_dem_chamois_deer)
mod.8.fs.lm <- lm(Female_survival ~ scale(Female_survival_1) + scale(P_apr.jul) + scale(N_deer_1) + scale(N_chamois), data = data_dem_chamois_deer)
mod.9.fs.lm <- lm(Female_survival ~ scale(Female_survival_1) + scale(P_jan.mar) + scale(N_deer_1), data = data_dem_chamois_deer)
mod.10.fs.lm <- lm(Female_survival ~ scale(Female_survival_1) + scale(P_apr.jul) + scale(N_deer_1), data = data_dem_chamois_deer)
mod.11.fs.lm <- lm(Female_survival ~ scale(Female_survival_1) + scale(N_deer_1) + scale(N_chamois), data = data_dem_chamois_deer)
mod.12.fs.lm <- lm(Female_survival ~ scale(Female_survival_1) + scale(N_deer_1), data = data_dem_chamois_deer)
# then we fit models without red deer
mod.13.fs.lm <- lm(Female_survival ~ scale(Female_survival_1) + scale(P_jan.mar) * scale(N_chamois), data = data_dem_chamois_deer)
mod.14.fs.lm <- lm(Female_survival ~ scale(Female_survival_1) + scale(P_apr.jul) * scale(N_chamois), data = data_dem_chamois_deer)
mod.15.fs.lm <- lm(Female_survival ~ scale(Female_survival_1) + scale(P_jan.mar) + scale(N_chamois), data = data_dem_chamois_deer)
mod.16.fs.lm <- lm(Female_survival ~ scale(Female_survival_1) + scale(P_apr.jul) + scale(N_chamois), data = data_dem_chamois_deer)
mod.17.fs.lm <- lm(Female_survival ~ scale(Female_survival_1) + scale(P_jan.mar), data = data_dem_chamois_deer)
mod.18.fs.lm <- lm(Female_survival ~ scale(Female_survival_1) + scale(P_apr.jul), data = data_dem_chamois_deer)
mod.19.fs.lm <- lm(Female_survival ~ scale(Female_survival_1) + scale(N_chamois), data = data_dem_chamois_deer)
```

```r
#+++++++++++++++++++++++++++++++++++++++++++++++++++++++++++++++++++++++++++++++
# 6.4.4) Modeling adult male  survival
#+++++++++++++++++++++++++++++++++++++++++++++++++++++++++++++++++++++++++++++++

# we start with models including red deer
mod.1.ms.lm <- lm(Male_survival ~ scale(Male_survival_1) + scale(P_jan.mar) * scale(N_chamois) + scale(N_deer_1), data = data_dem_chamois_deer)
mod.2.ms.lm <- lm(Male_survival ~ scale(Male_survival_1) + scale(P_jan.mar) * scale(N_deer_1) + scale(N_chamois), data = data_dem_chamois_deer)
mod.3.ms.lm <- lm(Male_survival ~ scale(Male_survival_1) + scale(P_apr.jul) * scale(N_chamois) + scale(N_deer_1), data = data_dem_chamois_deer)
mod.4.ms.lm <- lm(Male_survival ~ scale(Male_survival_1) + scale(P_apr.jul) * scale(N_deer_1) + scale(N_chamois), data = data_dem_chamois_deer)
mod.5.ms.lm <- lm(Male_survival ~ scale(Male_survival_1) + scale(P_jan.mar) * scale(N_deer_1), data = data_dem_chamois_deer)
mod.6.ms.lm <- lm(Male_survival ~ scale(Male_survival_1) + scale(P_apr.jul) * scale(N_deer_1), data = data_dem_chamois_deer)
mod.7.ms.lm <- lm(Male_survival ~ scale(Male_survival_1) + scale(P_jan.mar) + scale(N_chamois) + scale(N_deer_1), data = data_dem_chamois_deer)
mod.8.ms.lm <- lm(Male_survival ~ scale(Male_survival_1) + scale(P_apr.jul) + scale(N_deer_1) + scale(N_chamois), data = data_dem_chamois_deer)
mod.9.ms.lm <- lm(Male_survival ~ scale(Male_survival_1) + scale(P_jan.mar) + scale(N_deer_1), data = data_dem_chamois_deer)
mod.10.ms.lm <- lm(Male_survival ~ scale(Male_survival_1) + scale(P_apr.jul) + scale(N_deer_1), data = data_dem_chamois_deer)
mod.11.ms.lm <- lm(Male_survival ~ scale(Male_survival_1) + scale(N_deer_1) + scale(N_chamois), data = data_dem_chamois_deer)
mod.12.ms.lm <- lm(Male_survival ~ scale(Male_survival_1) + scale(N_deer_1), data = data_dem_chamois_deer)
# then we fit models without red deer
mod.13.ms.lm <- lm(Male_survival ~ scale(Male_survival_1) + scale(P_jan.mar) * scale(N_chamois), data = data_dem_chamois_deer)
mod.14.ms.lm <- lm(Male_survival ~ scale(Male_survival_1) + scale(P_apr.jul) * scale(N_chamois), data = data_dem_chamois_deer)
mod.15.ms.lm <- lm(Male_survival ~ scale(Male_survival_1) + scale(P_jan.mar) + scale(N_chamois), data = data_dem_chamois_deer)
mod.16.ms.lm <- lm(Male_survival ~ scale(Male_survival_1) + scale(P_apr.jul) + scale(N_chamois), data = data_dem_chamois_deer)
mod.17.ms.lm <- lm(Male_survival ~ scale(Male_survival_1) + scale(P_jan.mar), data = data_dem_chamois_deer)
mod.18.ms.lm <- lm(Male_survival ~ scale(Male_survival_1) + scale(P_apr.jul), data = data_dem_chamois_deer)
mod.19.ms.lm <- lm(Male_survival ~ scale(Male_survival_1) + scale(N_chamois), data = data_dem_chamois_deer)
```

```r
#==============================================================================
# 7) BAYESIAN MODEL SELECTION
#==============================================================================
```

```r
#------------------------------------------------------------------------------
# 7.1) USING WAIC
#------------------------------------------------------------------------------

# birth rate
waic.1.br.stan <- waic(mod.1.br.stan)
waic.2.br.stan <- waic(mod.2.br.stan)
waic.3.br.stan <- waic(mod.3.br.stan)
waic.4.br.stan <- waic(mod.4.br.stan)
waic.5.br.stan <- waic(mod.5.br.stan)
waic.6.br.stan <- waic(mod.6.br.stan)
waic.7.br.stan <- waic(mod.7.br.stan)
```

```
waic.8.br.stan <- waic(mod.8.br.stan)
waic.9.br.stan <- waic(mod.9.br.stan)
waic.10.br.stan <- waic(mod.10.br.stan)
waic.11.br.stan <- waic(mod.11.br.stan)
waic.12.br.stan <- waic(mod.12.br.stan)
waic.13.br.stan <- waic(mod.13.br.stan)
waic.14.br.stan <- waic(mod.14.br.stan)
waic.15.br.stan <- waic(mod.15.br.stan)
waic.16.br.stan <- waic(mod.16.br.stan)
waic.17.br.stan <- waic(mod.17.br.stan)
waic.18.br.stan <- waic(mod.18.br.stan)
waic.19.br.stan <- waic(mod.19.br.stan)

print(loo_compare(waic.1.br.stan, waic.2.br.stan, waic.3.br.stan, waic.4.br.stan, waic.5.br.stan, waic.6.br.stan, waic.7.br.stan, waic.8.br.stan, waic.9.br.stan, waic.10.br.stan, waic.11.br.stan,
                  waic.12.br.stan, waic.13.br.stan, waic.14.br.stan, waic.15.br.stan, waic.16.br.stan, waic.17.br.stan, waic.18.br.stan, waic.19.br.stan), digits = 2)
               elpd_diff se_diff
mod.4.br.stan    0.00      0.00
mod.6.br.stan   -1.37      2.21
mod.3.br.stan   -3.23      4.34
mod.2.br.stan   -3.74      3.07
mod.8.br.stan   -4.98      4.17
mod.11.br.stan  -5.12      4.91
mod.7.br.stan   -6.03      4.70
mod.1.br.stan   -6.93      4.74
mod.10.br.stan  -7.72      4.00
mod.14.br.stan  -7.95      5.28
mod.18.br.stan  -8.99      4.98
mod.5.br.stan   -9.55      4.35
mod.16.br.stan -10.12      5.80
mod.12.br.stan -10.29      5.27
mod.9.br.stan  -11.98      5.19
mod.19.br.stan -12.38      7.74
mod.15.br.stan -13.20      7.30
mod.17.br.stan -13.33      6.63
mod.13.br.stan -13.76      7.21

# kid survival
waic.1.ks.stan <- waic(mod.1.ks.stan)
waic.2.ks.stan <- waic(mod.2.ks.stan)
waic.3.ks.stan <- waic(mod.3.ks.stan)
waic.4.ks.stan <- waic(mod.4.ks.stan)
waic.5.ks.stan <- waic(mod.5.ks.stan)
waic.6.ks.stan <- waic(mod.6.ks.stan)
waic.7.ks.stan <- waic(mod.7.ks.stan)
waic.8.ks.stan <- waic(mod.8.ks.stan)
waic.9.ks.stan <- waic(mod.9.ks.stan)
waic.10.ks.stan <- waic(mod.10.ks.stan)
waic.11.ks.stan <- waic(mod.11.ks.stan)
waic.12.ks.stan <- waic(mod.12.ks.stan)
waic.13.ks.stan <- waic(mod.13.ks.stan)
waic.14.ks.stan <- waic(mod.14.ks.stan)
waic.15.ks.stan <- waic(mod.15.ks.stan)
waic.16.ks.stan <- waic(mod.16.ks.stan)
waic.17.ks.stan <- waic(mod.17.ks.stan)
waic.18.ks.stan <- waic(mod.18.ks.stan)
waic.19.ks.stan <- waic(mod.19.ks.stan)

print(loo_compare(waic.1.ks.stan, waic.2.ks.stan, waic.3.ks.stan, waic.4.ks.stan, waic.5.ks.stan, waic.6.ks.stan, waic.7.ks.stan, waic.8.ks.stan, waic.9.ks.stan, waic.10.ks.stan, waic.11.ks.stan,
                  waic.12.ks.stan, waic.13.ks.stan, waic.14.ks.stan, waic.15.ks.stan, waic.16.ks.stan, waic.17.ks.stan, waic.18.ks.stan, waic.19.ks.stan), digits = 2)
               elpd_diff se_diff
mod.13.ks.stan  0.00      0.00
mod.1.ks.stan  -0.73      0.71
mod.17.ks.stan -1.43      2.29
mod.18.ks.stan -1.53      1.90
mod.12.ks.stan -1.62      2.04
mod.19.ks.stan -1.71      2.06
mod.6.ks.stan  -2.18      2.38
mod.9.ks.stan  -2.43      2.21
mod.15.ks.stan -2.55      2.26
mod.10.ks.stan -2.57      1.92
```

```
mod.14.ks.stan -2.64      2.38
mod.16.ks.stan -2.74      1.99
mod.11.ks.stan -2.85      2.13
mod.5.ks.stan  -3.41      2.31
mod.4.ks.stan  -3.46      2.79
mod.7.ks.stan  -3.63      2.20
mod.8.ks.stan  -3.77      2.06
mod.3.ks.stan  -3.88      2.41
mod.2.ks.stan  -4.67      2.27

# adult female survival
waic.1.fs.stan <- waic(mod.1.fs.stan)
waic.2.fs.stan <- waic(mod.2.fs.stan)
waic.3.fs.stan <- waic(mod.3.fs.stan)
waic.4.fs.stan <- waic(mod.4.fs.stan)
waic.5.fs.stan <- waic(mod.5.fs.stan)
waic.6.fs.stan <- waic(mod.6.fs.stan)
waic.7.fs.stan <- waic(mod.7.fs.stan)
waic.8.fs.stan <- waic(mod.8.fs.stan)
waic.9.fs.stan <- waic(mod.9.fs.stan)
waic.10.fs.stan <- waic(mod.10.fs.stan)
waic.11.fs.stan <- waic(mod.11.fs.stan)
waic.12.fs.stan <- waic(mod.12.fs.stan)
waic.13.fs.stan <- waic(mod.13.fs.stan)
waic.14.fs.stan <- waic(mod.14.fs.stan)
waic.15.fs.stan <- waic(mod.15.fs.stan)
waic.16.fs.stan <- waic(mod.16.fs.stan)
waic.17.fs.stan <- waic(mod.17.fs.stan)
waic.18.fs.stan <- waic(mod.18.fs.stan)
waic.19.fs.stan <- waic(mod.19.fs.stan)

print(loo_compare(waic.1.fs.stan, waic.2.fs.stan, waic.3.fs.stan, waic.4.fs.stan, waic.5.fs.stan, waic.6.fs.stan, waic.7.fs.stan, waic.8.fs.stan, waic.9.fs.stan, waic.10.fs.stan, waic.11.fs.stan,
                  waic.12.fs.stan, waic.13.fs.stan, waic.14.fs.stan, waic.15.fs.stan, waic.16.fs.stan, waic.17.fs.stan, waic.18.fs.stan, waic.19.fs.stan), digits = 2)
               elpd_diff se_diff
mod.1.fs.stan    0.00      0.00
mod.12.fs.stan  -3.74      3.73
mod.10.fs.stan  -4.63      3.85
mod.11.fs.stan  -4.74      3.19
mod.8.fs.stan   -5.40      3.31
mod.6.fs.stan   -5.49      3.81
mod.9.fs.stan   -5.73      4.25
mod.4.fs.stan   -6.08      3.31
mod.13.fs.stan  -6.33      3.69
mod.3.fs.stan   -6.39      3.26
mod.7.fs.stan   -6.64      3.68
mod.5.fs.stan   -6.93      4.41
mod.2.fs.stan   -7.53      3.72
mod.18.fs.stan -10.76      4.15
mod.19.fs.stan -11.09      4.33
mod.17.fs.stan -11.67      4.57
mod.16.fs.stan -11.93      4.29
mod.14.fs.stan -12.08      4.23
mod.15.fs.stan -12.96      4.76

# adult male survival
waic.1.ms.stan <- waic(mod.1.ms.stan)
waic.2.ms.stan <- waic(mod.2.ms.stan)
waic.3.ms.stan <- waic(mod.3.ms.stan)
waic.4.ms.stan <- waic(mod.4.ms.stan)
waic.5.ms.stan <- waic(mod.5.ms.stan)
waic.6.ms.stan <- waic(mod.6.ms.stan)
waic.7.ms.stan <- waic(mod.7.ms.stan)
waic.8.ms.stan <- waic(mod.8.ms.stan)
waic.9.ms.stan <- waic(mod.9.ms.stan)
waic.10.ms.stan <- waic(mod.10.ms.stan)
waic.11.ms.stan <- waic(mod.11.ms.stan)
waic.12.ms.stan <- waic(mod.12.ms.stan)
waic.13.ms.stan <- waic(mod.13.ms.stan)
waic.14.ms.stan <- waic(mod.14.ms.stan)
waic.15.ms.stan <- waic(mod.15.ms.stan)
```

```
waic.16.ms.stan <- waic(mod.16.ms.stan)
waic.17.ms.stan <- waic(mod.17.ms.stan)
waic.18.ms.stan <- waic(mod.18.ms.stan)
waic.19.ms.stan <- waic(mod.19.ms.stan)

print(loo_compare(waic.1.ms.stan, waic.2.ms.stan, waic.3.ms.stan, waic.4.ms.stan, waic.5.ms.stan, waic.6.ms.stan, waic.7.ms.stan, waic.8.ms.stan, waic.9.ms.stan, waic.10.ms.stan, waic.11.ms.stan,
                  waic.12.ms.stan, waic.13.ms.stan, waic.14.ms.stan, waic.15.ms.stan, waic.16.ms.stan, waic.17.ms.stan, waic.18.ms.stan, waic.19.ms.stan), digits = 2)
               elpd_diff se_diff
mod.11.ms.stan  0.00      0.00
mod.8.ms.stan  -0.77      1.16
mod.7.ms.stan  -1.09      0.38
mod.2.ms.stan  -1.23      2.10
mod.1.ms.stan  -1.61      0.97
mod.5.ms.stan  -1.68      2.93
mod.12.ms.stan -1.67      2.14
mod.3.ms.stan  -1.87      1.33
mod.4.ms.stan  -2.17      1.44
mod.10.ms.stan -2.51      2.05
mod.9.ms.stan  -2.76      2.09
mod.19.ms.stan -3.80      3.46
mod.6.ms.stan  -3.93      2.23
mod.17.ms.stan -4.27      3.53
mod.18.ms.stan -4.29      3.41
mod.15.ms.stan -4.40      3.50
mod.16.ms.stan -4.54      3.17
mod.13.ms.stan -5.24      3.23
mod.14.ms.stan -5.44      2.95

#------------------------------------------------------------------------------
# 7.2) USING LOO-CV
#------------------------------------------------------------------------------

# birth rate
loo.1.br.stan <- loo(mod.1.br.stan, k_threshold = 0.7)
loo.2.br.stan <- loo(mod.2.br.stan, k_threshold = 0.7)
loo.3.br.stan <- loo(mod.3.br.stan, k_threshold = 0.7)
loo.4.br.stan <- loo(mod.4.br.stan, k_threshold = 0.7)
loo.5.br.stan <- loo(mod.5.br.stan, k_threshold = 0.7)
loo.6.br.stan <- loo(mod.6.br.stan, k_threshold = 0.7)
loo.7.br.stan <- loo(mod.7.br.stan, k_threshold = 0.7)
loo.8.br.stan <- loo(mod.8.br.stan, k_threshold = 0.7)
loo.9.br.stan <- loo(mod.9.br.stan, k_threshold = 0.7)
loo.10.br.stan <- loo(mod.10.br.stan, k_threshold = 0.7)
loo.11.br.stan <- loo(mod.11.br.stan, k_threshold = 0.7)
loo.12.br.stan <- loo(mod.12.br.stan, k_threshold = 0.7)
loo.13.br.stan <- loo(mod.13.br.stan, k_threshold = 0.7)
loo.14.br.stan <- loo(mod.14.br.stan, k_threshold = 0.7)
loo.15.br.stan <- loo(mod.15.br.stan, k_threshold = 0.7)
loo.16.br.stan <- loo(mod.16.br.stan, k_threshold = 0.7)
loo.17.br.stan <- loo(mod.17.br.stan, k_threshold = 0.7)
loo.18.br.stan <- loo(mod.18.br.stan, k_threshold = 0.7)
loo.19.br.stan <- loo(mod.19.br.stan, k_threshold = 0.7)

print(loo_compare(loo.1.br.stan, loo.2.br.stan, loo.3.br.stan, loo.4.br.stan, loo.5.br.stan, loo.6.br.stan, loo.7.br.stan, loo.8.br.stan, loo.9.br.stan, loo.10.br.stan,
                  loo.11.br.stan, loo.12.br.stan, loo.13.br.stan, loo.14.br.stan, loo.15.br.stan, loo.16.br.stan, loo.17.br.stan, loo.18.br.stan, loo.19.br.stan), digits = 2)
               elpd_diff se_diff
mod.4.br.stan    0.00      0.00
mod.6.br.stan   -0.32      2.58
mod.3.br.stan   -3.21      4.51
mod.2.br.stan   -4.55      3.73
mod.8.br.stan   -4.87      4.42
mod.11.br.stan  -5.11      5.27
mod.7.br.stan   -6.16      5.10
mod.1.br.stan   -6.75      5.04
mod.10.br.stan  -7.04      3.81
mod.14.br.stan  -7.50      5.10
mod.18.br.stan  -7.94      4.36
mod.16.br.stan  -9.45      5.48
mod.12.br.stan  -9.46      5.01
mod.5.br.stan   -9.51      4.63
```

```
mod.9.br.stan  -9.86       4.98
mod.19.br.stan -11.50      7.30
mod.17.br.stan -12.30      6.06
mod.15.br.stan -12.48      6.94
mod.13.br.stan -12.57      6.86


# kid survival
loo.1.ks.stan <- loo(mod.1.ks.stan, k_threshold = 0.7)
loo.2.ks.stan <- loo(mod.2.ks.stan, k_threshold = 0.7)
loo.3.ks.stan <- loo(mod.3.ks.stan, k_threshold = 0.7)
loo.4.ks.stan <- loo(mod.4.ks.stan, k_threshold = 0.7)
loo.5.ks.stan <- loo(mod.5.ks.stan, k_threshold = 0.7)
loo.6.ks.stan <- loo(mod.6.ks.stan, k_threshold = 0.7)
loo.7.ks.stan <- loo(mod.7.ks.stan, k_threshold = 0.7)
loo.8.ks.stan <- loo(mod.8.ks.stan, k_threshold = 0.7)
loo.9.ks.stan <- loo(mod.9.ks.stan, k_threshold = 0.7)
loo.10.ks.stan <- loo(mod.10.ks.stan, k_threshold = 0.7)
loo.11.ks.stan <- loo(mod.11.ks.stan, k_threshold = 0.7)
loo.12.ks.stan <- loo(mod.12.ks.stan, k_threshold = 0.7)
loo.13.ks.stan <- loo(mod.13.ks.stan, k_threshold = 0.7)
loo.14.ks.stan <- loo(mod.14.ks.stan, k_threshold = 0.7)
loo.15.ks.stan <- loo(mod.15.ks.stan, k_threshold = 0.7)
loo.16.ks.stan <- loo(mod.16.ks.stan, k_threshold = 0.7)
loo.17.ks.stan <- loo(mod.17.ks.stan, k_threshold = 0.7)
loo.18.ks.stan <- loo(mod.18.ks.stan, k_threshold = 0.7)
loo.19.ks.stan <- loo(mod.19.ks.stan, k_threshold = 0.7)

print(loo_compare(loo.1.ks.stan, loo.2.ks.stan, loo.3.ks.stan, loo.4.ks.stan, loo.5.ks.stan, loo.6.ks.stan, loo.7.ks.stan, loo.8.ks.stan, loo.9.ks.stan, loo.10.ks.stan,
                  loo.11.ks.stan, loo.12.ks.stan, loo.13.ks.stan, loo.14.ks.stan, loo.15.ks.stan, loo.16.ks.stan, loo.17.ks.stan, loo.18.ks.stan, loo.19.ks.stan), digits = 2)
               elpd_diff se_diff
mod.13.ks.stan  0.00      0.00
mod.17.ks.stan -0.91      2.08
mod.18.ks.stan -1.04      1.98
mod.1.ks.stan  -1.25      0.80
mod.19.ks.stan -1.27      2.15
mod.12.ks.stan -1.29      2.17
mod.6.ks.stan  -1.86      2.48
mod.15.ks.stan -2.15      2.06
mod.10.ks.stan -2.22      2.03
mod.9.ks.stan  -2.23      2.13
mod.14.ks.stan -2.37      2.37
mod.16.ks.stan -2.40      2.08
mod.11.ks.stan -2.60      2.27
mod.5.ks.stan  -3.34      2.22
mod.4.ks.stan  -3.52      2.98
mod.8.ks.stan  -3.57      2.18
mod.7.ks.stan  -3.65      2.24
mod.3.ks.stan  -3.90      2.44
mod.2.ks.stan  -4.90      2.26


# adult female survival
loo.1.fs.stan <- loo(mod.1.fs.stan, k_threshold = 0.7)
loo.2.fs.stan <- loo(mod.2.fs.stan, k_threshold = 0.7)
loo.3.fs.stan <- loo(mod.3.fs.stan, k_threshold = 0.7)
loo.4.fs.stan <- loo(mod.4.fs.stan, k_threshold = 0.7)
loo.5.fs.stan <- loo(mod.5.fs.stan, k_threshold = 0.7)
loo.6.fs.stan <- loo(mod.6.fs.stan, k_threshold = 0.7)
loo.7.fs.stan <- loo(mod.7.fs.stan, k_threshold = 0.7)
loo.8.fs.stan <- loo(mod.8.fs.stan, k_threshold = 0.7)
loo.9.fs.stan <- loo(mod.9.fs.stan, k_threshold = 0.7)
loo.10.fs.stan <- loo(mod.10.fs.stan, k_threshold = 0.7)
loo.11.fs.stan <- loo(mod.11.fs.stan, k_threshold = 0.7)
loo.12.fs.stan <- loo(mod.12.fs.stan, k_threshold = 0.7)
loo.13.fs.stan <- loo(mod.13.fs.stan, k_threshold = 0.7)
loo.14.fs.stan <- loo(mod.14.fs.stan, k_threshold = 0.7)
loo.15.fs.stan <- loo(mod.15.fs.stan, k_threshold = 0.7)
loo.16.fs.stan <- loo(mod.16.fs.stan, k_threshold = 0.7)
loo.17.fs.stan <- loo(mod.17.fs.stan, k_threshold = 0.7)
```

```
loo.18.fs.stan <- loo(mod.18.fs.stan, k_threshold = 0.7)
loo.19.fs.stan <- loo(mod.19.fs.stan, k_threshold = 0.7)

print(loo_compare(loo.1.fs.stan, loo.2.fs.stan, loo.3.fs.stan, loo.4.fs.stan, loo.5.fs.stan, loo.6.fs.stan, loo.7.fs.stan, loo.8.fs.stan, loo.9.fs.stan, loo.10.fs.stan,
                  loo.11.fs.stan, loo.12.fs.stan, loo.13.fs.stan, loo.14.fs.stan, loo.15.fs.stan, loo.16.fs.stan, loo.17.fs.stan, loo.18.fs.stan, loo.19.fs.stan), digits = 2)
               elpd_diff se_diff
mod.1.fs.stan    0.00      0.00
mod.12.fs.stan  -3.44      3.69
mod.10.fs.stan  -4.38      3.85
mod.11.fs.stan  -4.55      3.13
mod.8.fs.stan   -5.29      3.34
mod.6.fs.stan   -5.33      3.82
mod.4.fs.stan   -6.01      3.35
mod.9.fs.stan   -6.10      4.55
mod.13.fs.stan  -6.21      3.78
mod.3.fs.stan   -6.36      3.30
mod.7.fs.stan   -7.23      4.13
mod.5.fs.stan   -7.51      4.77
mod.2.fs.stan   -8.27      4.15
mod.18.fs.stan -10.44      4.18
mod.19.fs.stan -10.80      4.36
mod.16.fs.stan -11.71      4.33
mod.17.fs.stan -11.86      4.89
mod.14.fs.stan -12.68      4.32
mod.15.fs.stan -13.37      5.15


# adult male survival
loo.1.ms.stan <- loo(mod.1.ms.stan, k_threshold = 0.7)
loo.2.ms.stan <- loo(mod.2.ms.stan, k_threshold = 0.7)
loo.3.ms.stan <- loo(mod.3.ms.stan, k_threshold = 0.7)
loo.4.ms.stan <- loo(mod.4.ms.stan, k_threshold = 0.7)
loo.5.ms.stan <- loo(mod.5.ms.stan, k_threshold = 0.7)
loo.6.ms.stan <- loo(mod.6.ms.stan, k_threshold = 0.7)
loo.7.ms.stan <- loo(mod.7.ms.stan, k_threshold = 0.7)
loo.8.ms.stan <- loo(mod.8.ms.stan, k_threshold = 0.7)
loo.9.ms.stan <- loo(mod.9.ms.stan, k_threshold = 0.7)
loo.10.ms.stan <- loo(mod.10.ms.stan, k_threshold = 0.7)
loo.11.ms.stan <- loo(mod.11.ms.stan, k_threshold = 0.7)
loo.12.ms.stan <- loo(mod.12.ms.stan, k_threshold = 0.7)
loo.13.ms.stan <- loo(mod.13.ms.stan, k_threshold = 0.7)
loo.14.ms.stan <- loo(mod.14.ms.stan, k_threshold = 0.7)
loo.15.ms.stan <- loo(mod.15.ms.stan, k_threshold = 0.7)
loo.16.ms.stan <- loo(mod.16.ms.stan, k_threshold = 0.7)
loo.17.ms.stan <- loo(mod.17.ms.stan, k_threshold = 0.7)
loo.18.ms.stan <- loo(mod.18.ms.stan, k_threshold = 0.7)
loo.19.ms.stan <- loo(mod.19.ms.stan, k_threshold = 0.7)

print(loo_compare(loo.1.ms.stan, loo.2.ms.stan, loo.3.ms.stan, loo.4.ms.stan, loo.5.ms.stan, loo.6.ms.stan, loo.7.ms.stan, loo.8.ms.stan, loo.9.ms.stan, loo.10.ms.stan,
                  loo.11.ms.stan, loo.12.ms.stan, loo.13.ms.stan, loo.14.ms.stan, loo.15.ms.stan, loo.16.ms.stan, loo.17.ms.stan, loo.18.ms.stan, loo.19.ms.stan), digits = 2)
               elpd_diff se_diff
mod.11.ms.stan -0.00      0.00
mod.8.ms.stan  -0.16      1.71
mod.5.ms.stan  -0.81      3.39
mod.7.ms.stan  -1.29      0.47
mod.12.ms.stan -1.51      2.13
mod.2.ms.stan  -1.65      2.07
mod.3.ms.stan  -2.23      1.46
mod.1.ms.stan  -2.26      1.08
mod.10.ms.stan -2.48      2.06
mod.9.ms.stan  -2.81      2.09
mod.4.ms.stan  -2.97      1.70
mod.19.ms.stan -3.26      3.12
mod.18.ms.stan -3.42      2.91
mod.17.ms.stan -3.88      3.28
mod.15.ms.stan -4.12      3.30
mod.16.ms.stan -4.17      2.96
mod.6.ms.stan  -4.35      2.38
mod.14.ms.stan -4.61      2.46
mod.13.ms.stan -5.19      3.09
```

```
#================================================================================
# 8) OLS MODEL SELECTION
#================================================================================
```

```
#-----------------------------------------------------------------------------
# 8.1) USING AICc
#-----------------------------------------------------------------------------

# birth rate
selection.br.lm <- model.sel(mod.1.br.lm, mod.2.br.lm, mod.3.br.lm, mod.4.br.lm, mod.5.br.lm, mod.6.br.lm, mod.7.br.lm, mod.8.br.lm, mod.9.br.lm,
                             mod.10.br.lm, mod.11.br.lm, mod.12.br.lm, mod.13.br.lm, mod.14.br.lm, mod.15.br.lm, mod.16.br.lm, mod.17.br.lm, mod.18.br.lm, mod.19.br.lm)
selection.br.lm
```

Models name	AICc	delta	weight	Models name	AICc	delta	weight
mod.4.br.lm	-96.0	0.00	0.670	mod.18.br.lm	-82.7	23.85	0.001
mod.6.br.lm	-93.7	2.25	0.217	mod.12.br.lm	-80.6	26.59	0.000
mod.3.br.lm	-90.1	5.88	0.035	mod.16.br.lm	-80.5	28.63	0.000
mod.11.br.lm	-89.8	6.15	0.031	mod.5.br.lm	-79.1	28.72	0.000
mod.2.br.lm	-88.9	7.03	0.020	mod.9.br.lm	-77.9	29.37	0.000
mod.8.br.lm	-88.3	7.65	0.015	mod.19.br.lm	-76.9	31.19	0.000
mod.7.br.lm	-86.5	9.42	0.006	mod.17.br.lm	-74.5	32.32	0.000
mod.10.br.lm	-84.1	11.90	0.002	mod.15.br.lm	-74.3	33.52	0.000
mod.1.br.lm	-83.4	12.58	0.001	mod.13.br.lm	-71.9	23.85	0.000
mod.14.br.lm	-83.0	12.97	0.001				

```
# kid survival
selection.ks.lm <- model.sel(mod.1.ks.lm, mod.2.ks.lm, mod.3.ks.lm, mod.4.ks.lm,mod.5.ks.lm, mod.6.ks.lm, mod.7.ks.lm, mod.8.ks.lm, mod.9.ks.lm,
                             mod.10.ks.lm, mod.11.ks.lm, mod.12.ks.lm, mod.13.ks.lm, mod.14.ks.lm, mod.15.ks.lm, mod.16.ks.lm, mod.17.ks.lm, mod.18.ks.lm, mod.19.ks.lm)
selection.ks.lm
```

Models name	AICc	delta	weight	Models name	AICc	delta	weight
mod.17.ks.lm	-100.6	0.00	0.244	mod.16.ks.lm	-95.9	4.78	0.022
mod.13.ks.lm	-99.9	0.74	0.169	mod.6.ks.lm	-95.3	5.35	0.017
mod.12.ks.lm	-99.0	1.60	0.110	mod.14.ks.lm	-94.6	6.08	0.012
mod.18.ks.lm	-98.9	1.77	0.101	mod.5.ks.lm	-94.5	6.09	0.012
mod.19.ks.lm	-98.8	1.82	0.098	mod.7.ks.lm	-94.3	6.36	0.010
mod.9.ks.lm	-97.6	3.02	0.054	mod.8.ks.lm	-92.7	7.89	0.005
mod.15.ks.lm	-97.6	3.04	0.053	mod.4.ks.lm	-91.8	8.87	0.003
mod.1.ks.lm	-96.8	3.79	0.037	mod.3.ks.lm	-91.0	9.63	0.002
mod.10.ks.lm	-96.1	4.55	0.025	mod.2.ks.lm	-90.9	9.73	0.002
mod.11.ks.lm	-96.0	4.64	0.024				

```
# adult female survival
selection.fs.lm <- model.sel(mod.1.fs.lm, mod.2.fs.lm, mod.3.fs.lm, mod.4.fs.lm, mod.5.fs.lm, mod.6.fs.lm, mod.7.fs.lm, mod.8.fs.lm, mod.9.fs.lm,
                             mod.10.fs.lm, mod.11.fs.lm, mod.12.fs.lm,mod.13.fs.lm, mod.14.fs.lm, mod.15.fs.lm, mod.16.fs.lm, mod.17.fs.lm, mod.18.fs.lm, mod.19.fs.lm)
selection.fs.lm
```

Models name	AICc	delta	weight	Models name	AICc	delta	weight
mod.1.fs.lm	-65.8	0.00	0.795	mod.4.fs.lm	-53.2	12.64	0.001
mod.12.fs.lm	-61.7	4.13	0.101	mod.3.fs.lm	-52.9	12.97	0.001
mod.11.fs.lm	-59.3	6.57	0.030	mod.2.fs.lm	-52.6	13.26	0.001
mod.10.fs.lm	-58.8	6.99	0.024	mod.17.fs.lm	-47.5	18.37	0.000
mod.9.fs.lm	-58.7	7.16	0.022	mod.18.fs.lm	-47.4	18.45	0.000
mod.8.fs.lm	-56.5	9.30	0.008	mod.19.fs.lm	-47.4	18.45	0.000
mod.7.fs.lm	-56.0	9.86	0.006	mod.15.fs.lm	-44.4	21.40	0.000
mod.6.fs.lm	-55.6	10.22	0.005	mod.16.fs.lm	-44.4	21.48	0.000
mod.5.fs.lm	-55.4	10.45	0.004	mod.14.fs.lm	-41.2	24.60	0.000
mod.13.fs.lm	-54.0	11.86	0.002				

```
# adult male survival
selection.ms.lm <- model.sel(mod.1.ms.lm, mod.2.ms.lm, mod.3.ms.lm, mod.4.ms.lm, mod.5.ms.lm, mod.6.ms.lm, mod.7.ms.lm, mod.8.ms.lm, mod.9.ms.lm,
                             mod.10.ms.lm, mod.11.ms.lm, mod.12.ms.lm,mod.13.ms.lm, mod.14.ms.lm, mod.15.ms.lm, mod.16.ms.lm, mod.17.ms.lm, mod.18.ms.lm, mod.19.ms.lm)
selection.ms.lm
```

Models name	AICc	delta	weight	Models name	AICc	delta	weight
mod.11.ms.lm	-63.1	0.00	0.396	mod.3.ms.lm	-56.8	6.28	0.017
mod.12.ms.lm	-61.0	2.11	0.138	mod.19.ms.lm	-56.6	6.42	0.016
mod.8.ms.lm	-60.4	2.72	0.102	mod.17.ms.lm	-55.5	7.59	0.009
mod.7.ms.lm	-60.1	2.99	0.089	mod.18.ms.lm	-55.4	7.72	0.008

mod.5.ms.lm	-59.2	3.84	0.058	mod.6.ms.lm	-54.6	8.43	0.006
mod.2.ms.lm	-58.8	4.25	0.047	mod.15.ms.lm	-54.2	8.82	0.005
mod.10.ms.lm	-58.0	5.11	0.031	mod.16.ms.lm	-53.6	9.44	0.004
mod.9.ms.lm	-57.9	5.14	0.030	mod.13.ms.lm	-51.1	11.99	0.001
mod.1.ms.lm	-57.5	5.54	0.025	mod.14.ms.lm	-50.4	12.68	0.001
mod.4.ms.lm	-56.8	6.26	0.017				

```
#------------------------------------------------------------------------------
# 8.2) USING RMSE
#------------------------------------------------------------------------------
RMSE consists of 4 steps: 1) first, a given model is fitted to the original dataset, with free estimation of regression coefficients, and an ‘optimistic’ RMSE is calculated; 2) the original dataset is
then boostrapped 1000 times with replacement, and the model with the same structure as in point 1 is fitted to each bootstrap sample, with free estimation of regression coefficients, to obtain 1000 ‘tr
ain’ RMSE values; 3) next, the 1000 models obtained in point 2 are fitted to the original dataset, keeping both the structure and the regression coefficient estimates fixed, to obtain 1000 ‘test’ RMSE
values. The difference between the 1000 ‘train’ and ‘test’ RMSE values is averaged to obtain an estimate of the ‘optimism’ in RMSE for the given model. Finally, 4) the model performance corrected for o
ptimism-bias is calculated as: ‘optimistic’ RMSE - [average(‘train’ RMSE - ‘test’ RMSE)] (Steyenberg et al., 2001).

# Define function to extract the bias-corrected RMSE
boot.validation = function(model, dataset){ # create function to calculate bias-corrected RMSE
  valid.model <- ols(formula(model), data = dataset, x = TRUE, y = TRUE)
  validation.model <- validate(valid.model, B = 1000, bw = FALSE) # run bootstrap model validation (1000 bootstraps)
  RMSE.optimistic <- sqrt(validation.model[2,1]) # 'optimistic' RMSE on full data, free estimation of parameter
  RMSE.train <- sqrt(validation.model[2,2]) # mean 'train' RMSE on bootstrap samples, free estimation of parameter
  RMSE.test <- sqrt(validation.model[2,3]) # mean 'test' RMSE on full data, freezing parameter estimates for each bootstrap sample
  round((RMSE.optimistic - (RMSE.train-RMSE.test)), 3) # bias-corrected RMSE estimate
}

# birth rate
br.lm.model.name <- list("mod.1.br.lm", "mod.2.br.lm", "mod.3.br.lm", "mod.4.br.lm","mod.5.br.lm", "mod.6.br.lm", "mod.7.br.lm","mod.8.br.lm", "mod.9.br.lm", "mod.10.br.lm", "mod.11.br.lm",
                         "mod.12.br.lm","mod.13.br.lm", "mod.14.br.lm", "mod.15.br.lm", "mod.16.br.lm","mod.17.br.lm","mod.18.br.lm","mod.19.br.lm")

for (i in br.lm.model.name){
  br.lm.model.name[i] = boot.validation(get(i), data_dem_chamois_deer)} # apply bootstrap validation recursively to all the models in the list and return bias-corrected RMSE values

model.br <- (as.character(br.lm.model.name))[1:19] # extract model names
BIAS.CORRECTED.RMSE.br <- as.numeric((as.vector(br.lm.model.name))[20:38]) # extract bias-corrected RMSE values
RMSE.br.df <- as.data.frame(cbind(model.br, BIAS.CORRECTED.RMSE.br)) # create dataframe with model names and bias-corrected RMSE values
RMSE.br.df <- arrange(RMSE.br.df, BIAS.CORRECTED.RMSE.br) # sort bias-corrected RMSE values in ascending order
RMSE.br.df # mod.4.br.lm has the lowest RMSE value
       model.br BIAS.CORRECTED.RMSE.br
1   mod.4.br.lm                  0.041
2   mod.6.br.lm                  0.042
3   mod.3.br.lm                  0.044
4   mod.8.br.lm                  0.047
5  mod.11.br.lm                  0.047
6   mod.2.br.lm                  0.049
7   mod.7.br.lm                  0.049
8  mod.14.br.lm                  0.051
9   mod.1.br.lm                  0.052
10 mod.10.br.lm                  0.052
11 mod.18.br.lm                  0.053
12 mod.16.br.lm                  0.055
13 mod.12.br.lm                  0.057
14  mod.9.br.lm                  0.059
15  mod.5.br.lm                  0.059
16 mod.19.br.lm                  0.059
17 mod.15.br.lm                  0.062
18 mod.17.br.lm                  0.062
19 mod.13.br.lm                  0.064

# kid survival
ks.lm.model.name <- list("mod.1.ks.lm", "mod.2.ks.lm", "mod.3.ks.lm", "mod.4.ks.lm","mod.5.ks.lm", "mod.6.ks.lm", "mod.7.ks.lm", "mod.8.ks.lm", "mod.9.ks.lm", "mod.10.ks.lm", "mod.11.ks.lm",
                         "mod.12.ks.lm","mod.13.ks.lm", "mod.14.ks.lm", "mod.15.ks.lm", "mod.16.ks.lm","mod.17.ks.lm","mod.18.ks.lm","mod.19.ks.lm")

for (i in ks.lm.model.name){
  ks.lm.model.name[i] = boot.validation(get(i), data_dem_chamois_deer)} # apply bootstrap validation recursively to all the models in the list and return bias-corrected RMSE values

model.ks <- (as.character(ks.lm.model.name))[1:19] # extract model names
BIAS.CORRECTED.RMSE.ks <- as.numeric((as.vector(ks.lm.model.name))[20:38]) # extract bias-corrected RMSE values
```

```
RMSE.ks.df <- as.data.frame(cbind(model.ks, BIAS.CORRECTED.RMSE.ks)) # create dataframe with model names and bias-corrected RMSE values
RMSE.ks.df <- arrange(RMSE.ks.df, BIAS.CORRECTED.RMSE.ks) # sort bias-corrected RMSE values in ascending order
RMSE.ks.df # mod.12.ks.lm has the lowest value
       model.ks BIAS.CORRECTED.RMSE.ks
1  mod.12.ks.lm                  0.037
2  mod.13.ks.lm                  0.037
3  mod.18.ks.lm                  0.037
4  mod.19.ks.lm                  0.037
5   mod.1.ks.lm                  0.038
6   mod.6.ks.lm                  0.038
7  mod.10.ks.lm                  0.038
8  mod.16.ks.lm                  0.038
9  mod.17.ks.lm                  0.039
10  mod.9.ks.lm                  0.039
11 mod.11.ks.lm                  0.039
12 mod.15.ks.lm                  0.039
13  mod.4.ks.lm                  0.039
14  mod.5.ks.lm                  0.040
15  mod.8.ks.lm                  0.040
16 mod.14.ks.lm                  0.041
17  mod.3.ks.lm                  0.041
18  mod.7.ks.lm                  0.041
19  mod.2.ks.lm                  0.042

# adult female survival
fs.lm.model.name <- list("mod.1.fs.lm", "mod.2.fs.lm", "mod.3.fs.lm", "mod.4.fs.lm","mod.5.fs.lm", "mod.6.fs.lm", "mod.7.fs.lm", "mod.8.fs.lm", "mod.9.fs.lm", "mod.10.fs.lm", "mod.11.fs.lm",
                         "mod.12.fs.lm","mod.13.fs.lm", "mod.14.fs.lm", "mod.15.fs.lm", "mod.16.fs.lm","mod.17.fs.lm","mod.18.fs.lm","mod.19.fs.lm")

for (i in fs.lm.model.name){
  fs.lm.model.name[i] = boot.validation(get(i), data_dem_chamois_deer)} # apply bootstrap validation recursively to all the models in the list and return bias-corrected RMSE values

model.fs <- (as.character(fs.lm.model.name))[1:19] # extract model names
BIAS.CORRECTED.RMSE.fs <- as.numeric((as.vector(fs.lm.model.name))[20:38]) # extract bias-corrected RMSE values
RMSE.fs.df <- as.data.frame(cbind(model.fs, BIAS.CORRECTED.RMSE.fs)) # create dataframe with model names and bias-corrected RMSE values
RMSE.fs.df <- arrange(RMSE.fs.df, BIAS.CORRECTED.RMSE.fs) # sort bias-corrected RMSE values in ascending order
RMSE.fs.df # mod.12.fs.lm has the lowest value
       model.fs BIAS.CORRECTED.RMSE.fs
1   mod.1.fs.lm                  0.065
2  mod.12.fs.lm                  0.070
3  mod.10.fs.lm                  0.072
4  mod.11.fs.lm                  0.073
5   mod.8.fs.lm                  0.075
6   mod.6.fs.lm                  0.075
7   mod.3.fs.lm                  0.078
8   mod.4.fs.lm                  0.079
9   mod.9.fs.lm                  0.079
10  mod.7.fs.lm                  0.081
11 mod.13.fs.lm                  0.081
12  mod.5.fs.lm                  0.085
13  mod.2.fs.lm                  0.086
14 mod.18.fs.lm                  0.091
15 mod.19.fs.lm                  0.094
16 mod.16.fs.lm                  0.095
17 mod.17.fs.lm                  0.098
18 mod.14.fs.lm                  0.100
19 mod.15.fs.lm                  0.102

# adult male survival
ms.lm.model.name <- list("mod.1.ms.lm", "mod.2.ms.lm", "mod.3.ms.lm", "mod.4.ms.lm","mod.5.ms.lm", "mod.6.ms.lm", "mod.7.ms.lm", "mod.8.ms.lm", "mod.9.ms.lm", "mod.10.ms.lm", "mod.11.ms.lm ",
                        "mod.12.ms.lm","mod.13.ms.lm", "mod.14.ms.lm", "mod.15.ms.lm", "mod.16.ms.lm","mod.17.ms.lm","mod.18.ms.lm","mod.19.ms.lm")

for (i in ms.lm.model.name){
  ms.lm.model.name[i] = boot.validation(get(i), data_dem_chamois_deer)} # apply bootstrap validation recursively to all the models in the list and return bias-corrected RMSE values

model.ms <- (as.character(ms.lm.model.name))[1:19] # extract model names
BIAS.CORRECTED.RMSE.ms <- as.numeric((as.vector(ms.lm.model.name))[20:38]) # extract bias-corrected RMSE values
RMSE.ms.df <- as.data.frame(cbind(model.ms, BIAS.CORRECTED.RMSE.ms)) # create dataframe with model names and bias-corrected RMSE values
RMSE.ms.df <- arrange(RMSE.ms.df, BIAS.CORRECTED.RMSE.ms) # sort bias-corrected RMSE values in ascending order
RMSE.ms.df # mod.11.ms.lm has the lowest value
```

```
       model.ms BIAS.CORRECTED.RMSE.ms
1  mod.11.ms.lm                  0.072
2  mod.8.ms.lm                   0.074
3  mod.7.ms.lm                   0.075
4  mod.2.ms.lm                   0.076
5  mod.12.ms.lm                  0.076
6   mod.3.ms.lm                  0.077
7   mod.5.ms.lm                  0.078
8  mod.10.ms.lm                  0.078
9   mod.4.ms.lm                  0.080
10  mod.9.ms.lm                  0.080
11 mod.19.ms.lm                  0.080
12 mod.15.ms.lm                  0.081
13 mod.17.ms.lm                  0.081
14 mod.18.ms.lm                  0.081
15 mod.16.ms.lm                  0.082
16  mod.1.ms.lm                  0.083
17  mod.6.ms.lm                  0.084
18 mod.14.ms.lm                  0.085
19 mod.13.ms.lm                  0.087
```

```
#==============================================================================
# 9) BAYESIAN MODEL VALIDATION
#==============================================================================

# birth rate
par(mfrow = c(1,1))
plot(mod.4.br.stan , "trace") # trace plots
pp_check(mod.4.br.stan) # posterior predictive check
launch_shinystan(mod.4.br.stan) # we can thoroughly check the fit of the model with Shinystan

predict.mod.4.br.stan  <- t(posterior_predict(mod.4.br.stan)) # residual diagnostics
sim.resid.mod.4.br.stan  <- createDHARMa(simulatedResponse = predict.mod.4.br.stan , observedResponse = data_dem_chamois_deer$Birth_rate_t)
plot(sim.resid.mod.4.br.stan) # overall fit
par(mfrow=c(3,1)) # plot the predicted residuals against each variable in the model
plotResiduals(sim.resid.mod.4.br.stan, form=data_dem_chamois_deer$P1_apr.jul, xlab = "P1_apr.jul")
plotResiduals(sim.resid.mod.4.br.stan, form=data_dem_chamois_deer$N_deer_1, xlab = "N_deer_1")
plotResiduals(sim.resid.mod.4.br.stan, form=data_dem_chamois_deer$N_chamois_1, xlab = "N_chamois_1")
testTemporalAutocorrelation(sim.resid.mod.4.br.stan, time = data_dem_chamois_deer$Year_N) # test temporal correlation
check_autocorrelation(mod.4.br.stan) # test autocorrelation
OK: Residuals appear to be independent and not autocorrelated (p = 0.852).
```

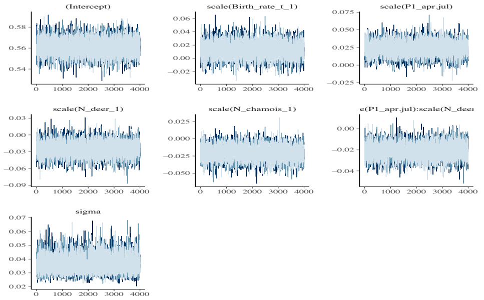

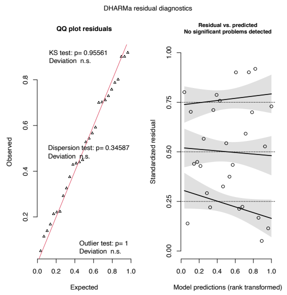

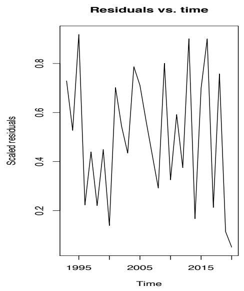

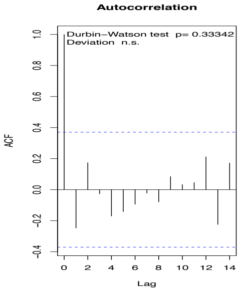


```
# kid survival
par(mfrow = c(1,1))
plot(mod.13.ks.stan , "trace") # trace plots
pp_check(mod.13.ks.stan) # posterior predictive check
launch_shinystan(mod.13.ks.stan) # we can thoroughly check the fit of the model with Shinystan

predict.mod.13.ks.stan  <- t(posterior_predict(mod.13.ks.stan)) # residual diagnostics
sim.resid.mod.13.ks.stan  <- createDHARMa(simulatedResponse = predict.mod.19.ks.stan , observedResponse = data_dem_chamois_deer$Kid_survival)
plot(sim.resid.mod.13.ks.stan) # overall fit
```

```
par(mfrow=c(2,1)) # plot the predicted residuals against each variable in the model
plotResiduals(sim.resid.mod.13.ks.stan, form=data_dem_chamois_deer$P1_jan.mar, xlab = "P_jan.mar")
plotResiduals(sim.resid.mod.13.ks.stan, form=data_dem_chamois_deer$N_chamois_1, xlab = "N_chamois")
testTemporalAutocorrelation(sim.resid.mod.13.ks.stan, time = (na.omit(data_dem_chamois_deer)$Year_N) # test temporal correlation
check_autocorrelation(mod.13.ks.stan) # test autocorrelation
OK: Residuals appear to be independent and not autocorrelated (p = 0.416).
```

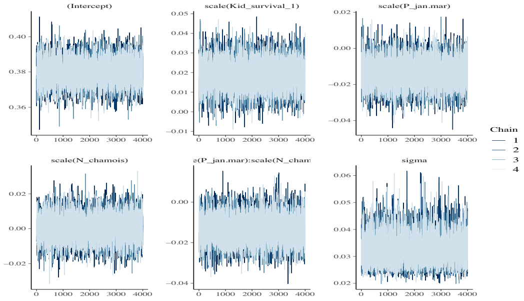

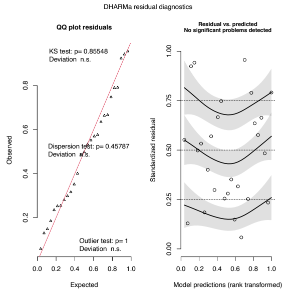

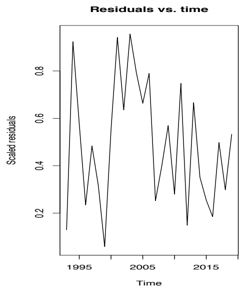

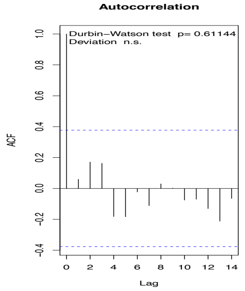


```
# adult female survival

par(mfrow = c(1,1))
plot(mod.1.fs.stan , "trace") # trace plots
pp_check(mod.1.fs.stan) # posterior predictive check
launch_shinystan(mod.1.fs.stan) # we can thoroughly check the fit of the model with Shinystan

predict.mod.1.fs.stan  <- t(posterior_predict(mod.1.fs.stan)) # residual diagnostics
sim.resid.mod.1.fs.stan  <- createDHARMa(simulatedResponse = predict.mod.1.fs.stan , observedResponse = data_dem_chamois_deer$Female_survival)
plot(sim.resid.mod.1.fs.stan) # overall fit
par(mfrow=c(3,1)) # plot the predicted residuals against each variable in the model
plotResiduals(sim.resid.mod.1.fs.stan, form=data_dem_chamois_deer$P_jan.mar, xlab = "P_jan.mar")
plotResiduals(sim.resid.mod.1.fs.stan, form=data_dem_chamois_deer$N_chamois, xlab = "N_chamois")
plotResiduals(sim.resid.mod.1.fs.stan, form=data_dem_chamois_deer$N_deer_1, xlab = "N_deer_1")
testTemporalAutocorrelation(sim.resid.mod.1.fs.stan, time = (na.omit(data_dem_chamois_deer)$Year_N) # test temporal correlation
check_autocorrelation(mod.1.fs.stan) # # test autocorrelation
OK: Residuals appear to be independent and not autocorrelated (p = 0.554).
```

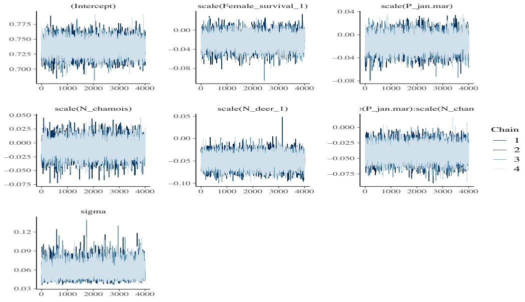

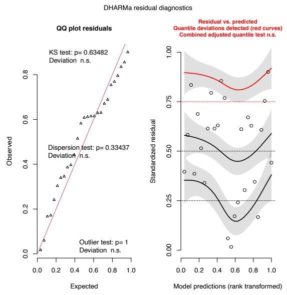

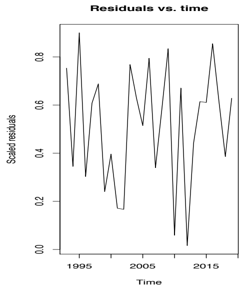

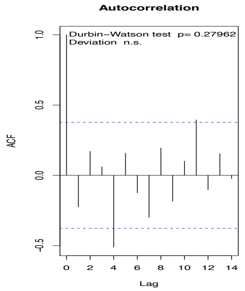


```
# adult male survival

par(mfrow = c(1,1))
plot(mod.11.ms.stan , "trace") # trace plots
pp_check(mod.11.ms.stan) # posterior predictive check
launch_shinystan(mod.11.ms.stan) # we can thoroughly check the fit of the model with Shinystan

predict.mod.11.ms.stan  <- t(posterior_predict(mod.11.ms.stan)) # residual diagnostics
```

```
sim.resid.mod.11.ms.stan  <- createDHARMa(simulatedResponse = predict.mod.11.ms.stan , observedResponse = data_dem_chamois_deer$Male_survival)
plot(sim.resid.mod.11.ms.stan) # overall fit
par(mfrow=c(2,1))
plotResiduals(sim.resid.mod.11.ms.stan, form=data_dem_chamois_deer$N_chamois, xlab = "N_chamois")
plotResiduals(sim.resid.mod.11.ms.stan, form=data_dem_chamois_deer$N_deer_1, xlab = "N_deer_1")
testTemporalAutocorrelation(sim.resid.mod.11.ms.stan, time = (na.omit(data_dem_chamois_deer)$Year_N) # test temporal correlation
check_autocorrelation(mod.11.ms.stan) # # test autocorrelation
OK: Residuals appear to be independent and not autocorrelated (p = 0.138).
```

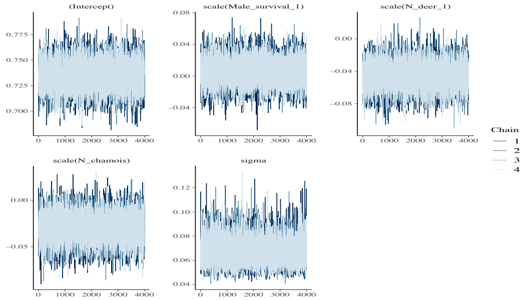

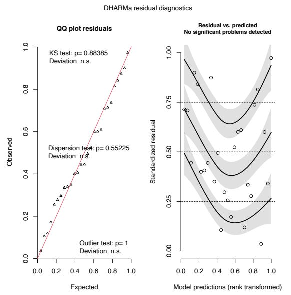

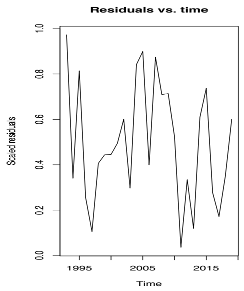

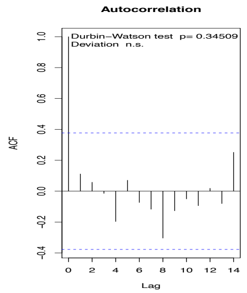


```
#==============================================================================
# 10) OLS MODEL VALIDATION
#==============================================================================

# model validation made for OLS models with delta AICc < 4

# birth rate
check_model(mod.4.br.lm)
sim.mod.4.br.lm <- simulateResiduals(mod.4.br.lm, n=5000)
plot(sim.mod.4.br.lm)
testTemporalAutocorrelation(sim.mod.4.br.lm, time = data_dem_chamois_deer)$Year_N # test temporal correlation
```

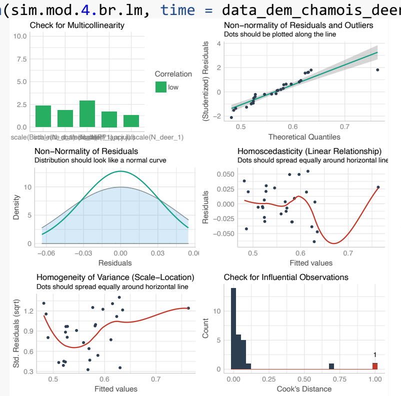

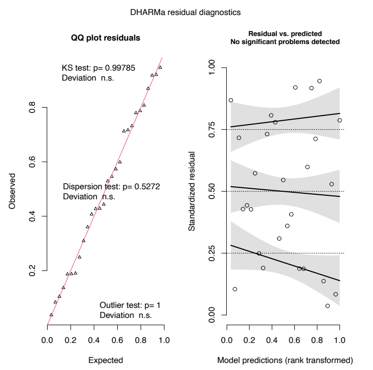

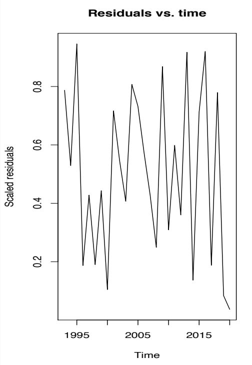

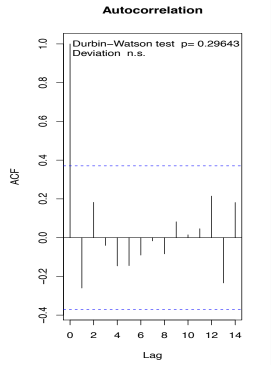


```
check_model(mod.6.br.lm)
sim.mod.6.br.lm <- simulateResiduals(mod.6.br.lm, n=5000)
plot(sim.mod.6.br.lm)
testTemporalAutocorrelation(sim.mod.6.br.lm, time = data_dem_chamois_deer$Year_N) # test temporal correlation
```

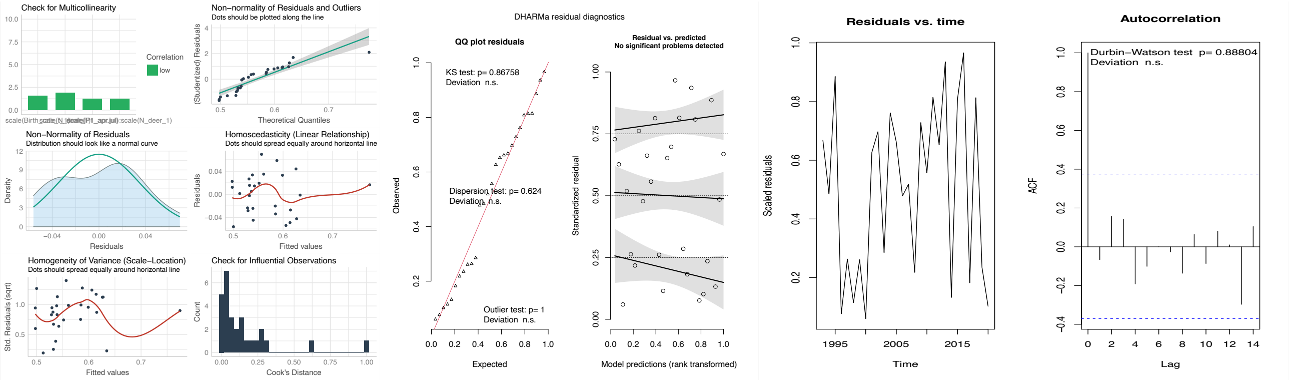


```
# kid survival

check_model(mod.17.ks.lm)
sim.mod.17.ks.lm <- simulateResiduals(mod.17.ks.lm)
plot(sim.mod.17.ks.lm)
testTemporalAutocorrelation(sim.mod.17.ks.lm, time = (na.omit(data_dem_chamois_deer)$Year_N) # test temporal correlation
```

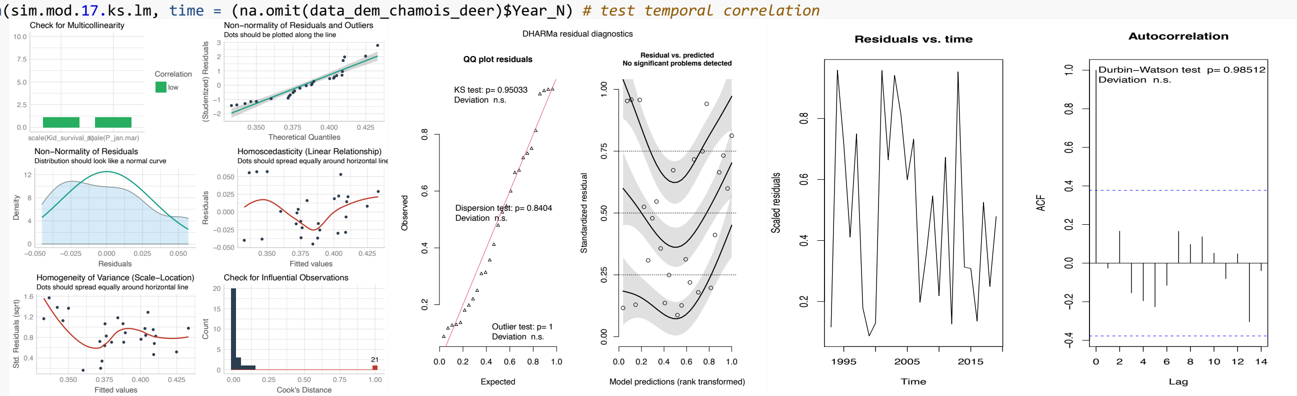


```
check_model(mod.13.ks.lm)
sim.mod.13.ks.lm <- simulateResiduals(mod.13.ks.lm)
plot(sim.mod.13.ks.lm)
testTemporalAutocorrelation(sim.mod.13.ks.lm, time = (na.omit(data_dem_chamois_deer)$Year_N) # test temporal correlation
```

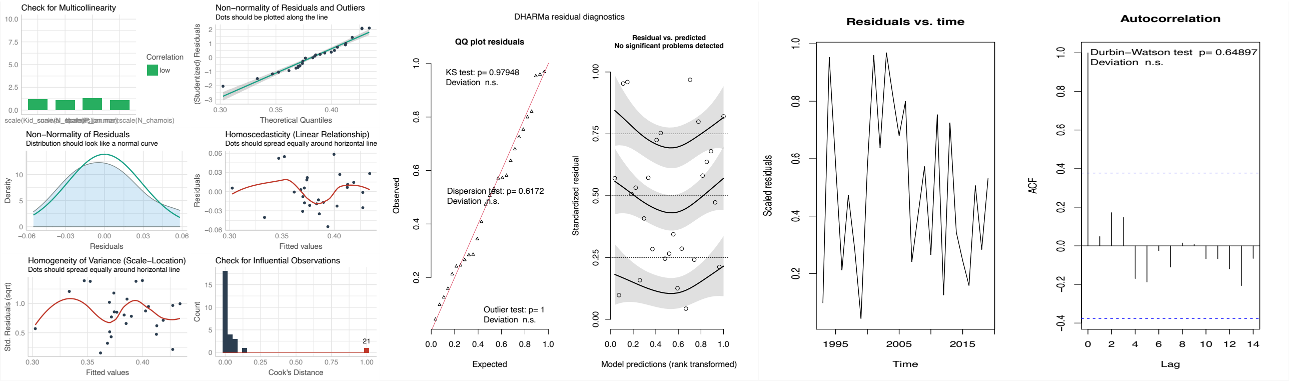


```
check_model(mod.12.ks.lm)
sim.mod.12.ks.lm <- simulateResiduals(mod.12.ks.lm)
plot(sim.mod.12.ks.lm)
testTemporalAutocorrelation(sim.mod.12.ks.lm, time = (na.omit(data_dem_chamois_deer)$Year_N) # test temporal correlation
```

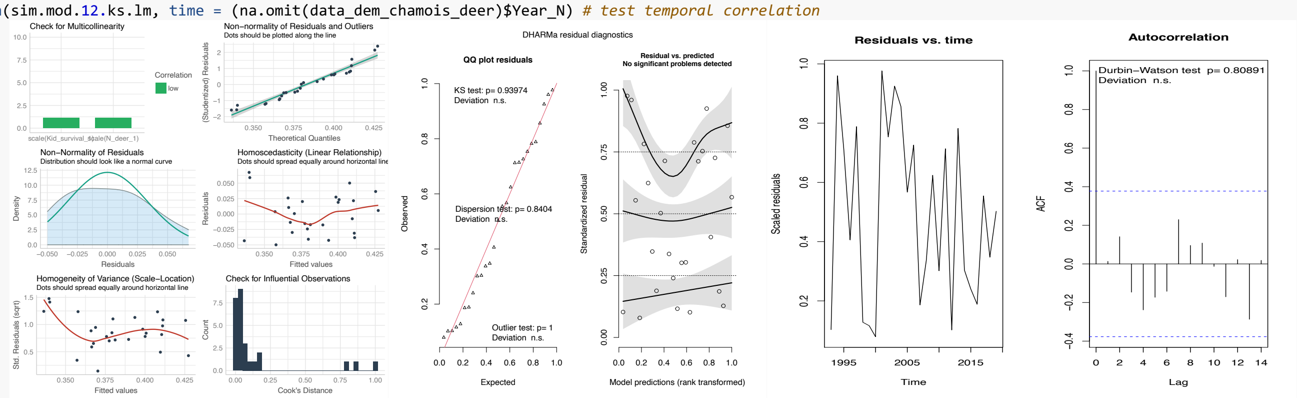


```
check_model(mod.18.ks.lm)
sim.mod.18.ks.lm <- simulateResiduals(mod.18.ks.lm)
plot(sim.mod.18.ks.lm)
testTemporalAutocorrelation(sim.mod.18.ks.lm, time = (na.omit(data_dem_chamois_deer)$Year_N) # test temporal correlation
```

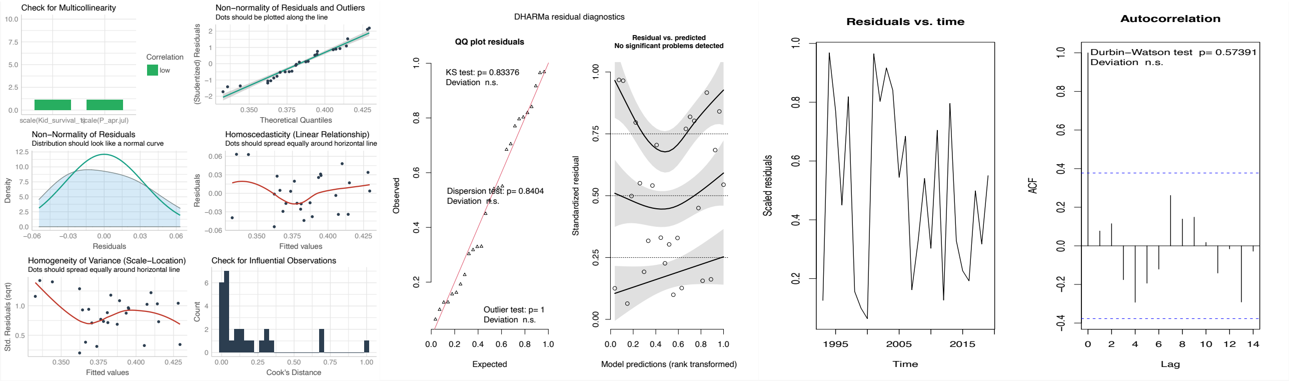


```
check_model(mod.19.ks.lm)
sim.mod.19.ks.lm <- simulateResiduals(mod.19.ks.lm)
plot(sim.mod.19.ks.lm)
testTemporalAutocorrelation(sim.mod.19.ks.lm, time = (na.omit(data_dem_chamois_deer)$Year_N) # test temporal correlation
```

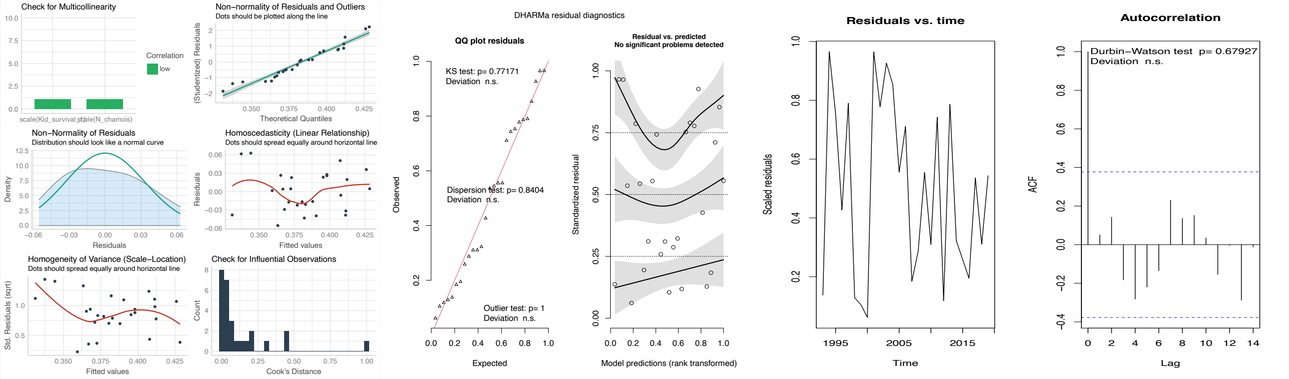


```
check_model(mod.9.ks.lm)
sim.mod.9.ks.lm <- simulateResiduals(mod.9.ks.lm)
plot(sim.mod.9.ks.lm)
testTemporalAutocorrelation(sim.mod.9.ks.lm, time = (na.omit(data_dem_chamois_deer)$Year_N) # test temporal correlation
```

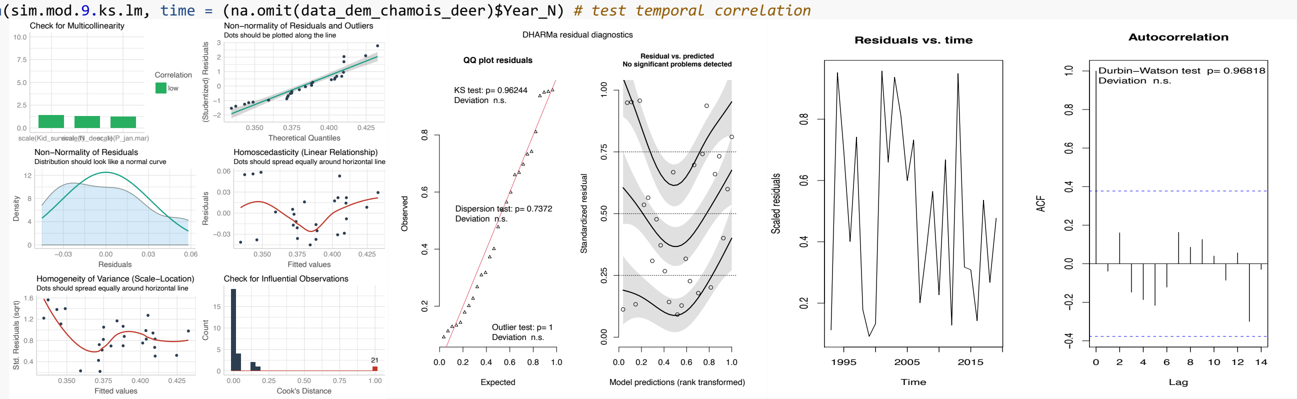

```
check_model(mod.15.ks.lm)
sim.mod.15.ks.lm <- simulateResiduals(mod.15.ks.lm)
plot(sim.mod.15.ks.lm)
testTemporalAutocorrelation(sim.mod.15.ks.lm, time = (na.omit(data_dem_chamois_deer)$Year_N) # test temporal correlation
```

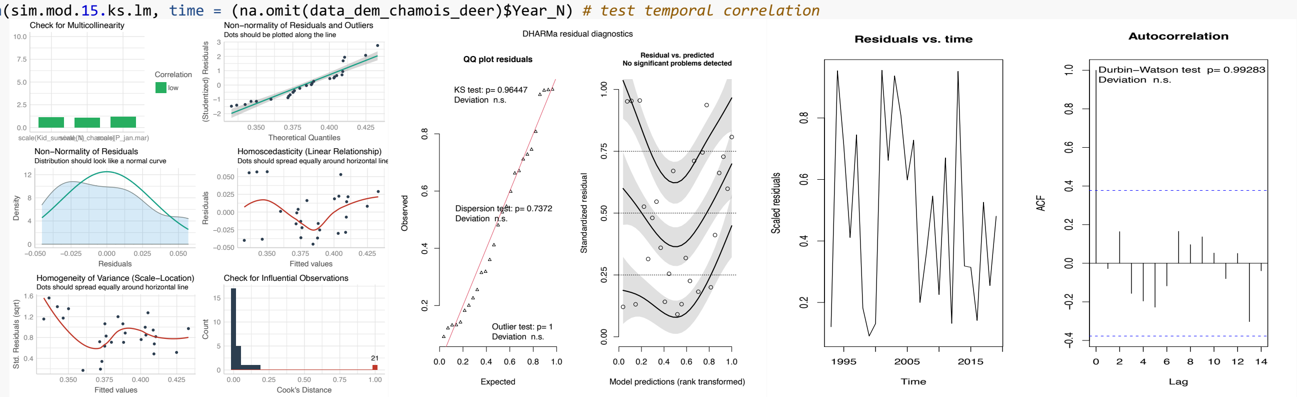


```
check_model(mod.1.ks.lm)
sim.mod.1.ks.lm <- simulateResiduals(mod.11.ks.lm)
plot(sim.mod.11.ks.lm)
testTemporalAutocorrelation(sim.mod.11.ks.lm, time = (na.omit(data_dem_chamois_deer)$Year_N) # test temporal correlation
```

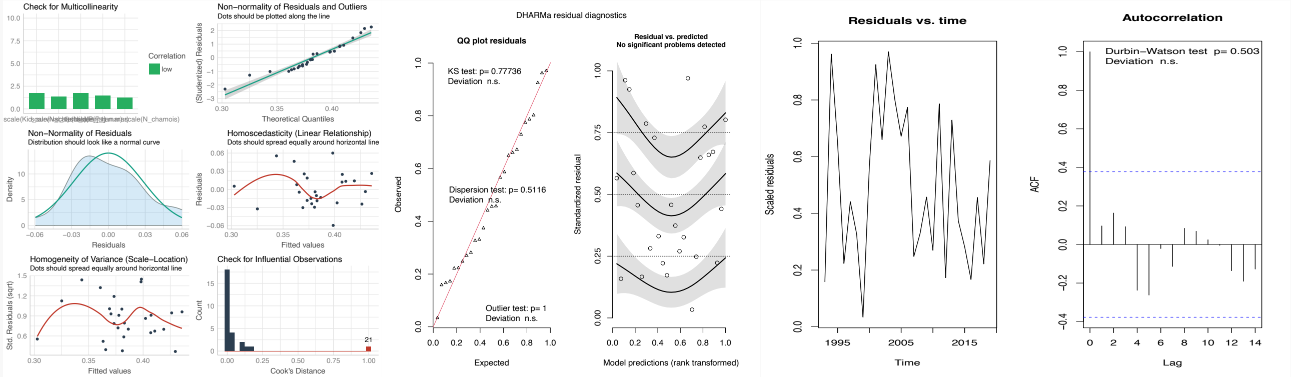


```
# adult female survival
check_model(mod.1.fs.lm)
sim.mod.1.fs.lm <- simulateResiduals(mod.1.fs.lm)
plot(sim.mod.1.fs.lm)
testTemporalAutocorrelation(sim.mod.1.fs.lm, time = (na.omit(data_dem_chamois_deer)$Year_N) # test temporal correlation
```

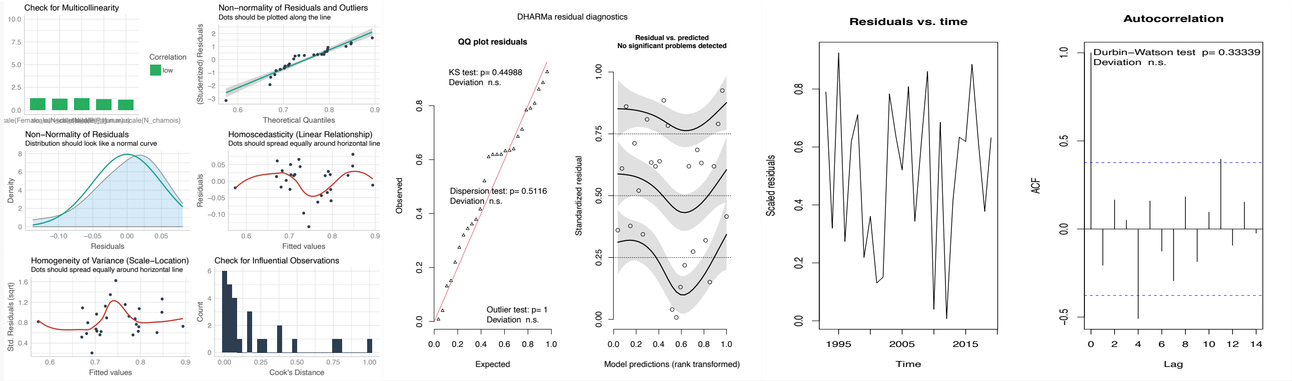


```
# adult male survival
check_model(mod.11.ms.lm)
```

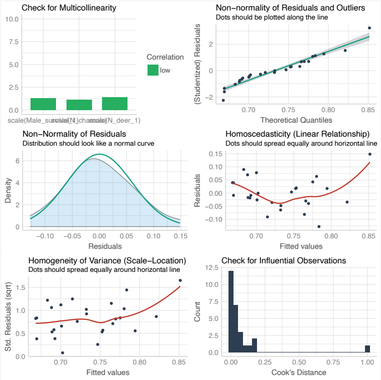


```
# a close inspection suggests that mod.11.ms.lm might suffer heteroskedasticity issues
check_heteroscedasticity(mod.11.ms.lm)
Warning: Heteroscedasticity (non-constant error variance) detected (p = 0.024).
# to check if this is consequential for inference, we run a wild bootstrap, which allows to maintain the
# nominal error rate in small samples under homoskedasticity, moderate heteroskedasticity and severe heteroskedasticity
Male_survival <- data_dem_chamois_deer$Male_survival
Male_survival_1.st <- scale(data_dem_chamois_deer$Male_survival_1)
N_deer_1.st <- scale(data_dem_chamois_deer$N_deer_1)
N_chamois.st <- scale(data_dem_chamois_deer$N_chamois)
mod.11.ms.lm.wild <- lm(Male_survival ~ Male_survival_1.st + N_deer_1.st + N_chamois.st)
print(Pboot(model = mod.11.ms.lm.wild, J = 1000, K = 100), digits = 2)
parameters(mod.11.ms.lm, ci=0.95, digits = 3, ci_digits=3)
Parameter       | Coefficient |    SE |           95% CI |  t(23) |      p
--------------------------------------------------------------------------
(Intercept)     |       0.736 | 0.012 | [ 0.710,  0.761] | 59.378 | < .001
Male_survival_1 |       0.006 | 0.014 | [-0.023,  0.035] |  0.423 | 0.676
N_deer_1        |      -0.046 | 0.015 | [-0.076, -0.015] | -3.106 | 0.005
N_chamois       |      -0.029 | 0.013 | [-0.057, -0.002] | -2.199 | 0.038
# the results are consistent with mod.11.ms.lm

sim.mod.11.ms.lm <- simulateResiduals(mod.11.ms.lm)
plot(sim.mod.11.ms.lm)
testTemporalAutocorrelation(sim.mod.11.ms.lm, time = (na.omit(data_dem_chamois_deer)$Year_N) # test temporal correlation
```

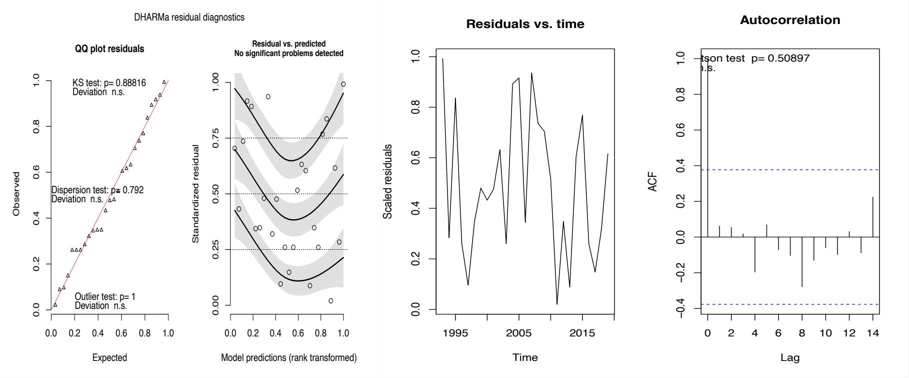


```
check_model(mod.12.ms.lm)
sim.mod.12.ms.lm <- simulateResiduals(mod.12.ms.lm)
plot(sim.mod.12.ms.lm)
testTemporalAutocorrelation(sim.mod.12.ms.lm, time = (na.omit(data_dem_chamois_deer)$Year_N) # test temporal correlation
```

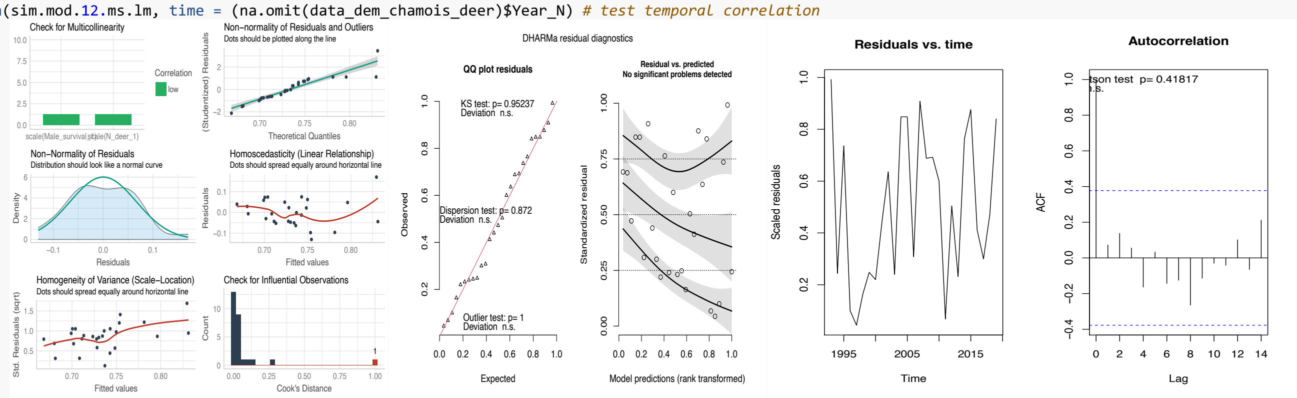


```
check_model(mod.8.ms.lm)
sim.mod.8.ms.lm <- simulateResiduals(mod.8.ms.lm)
plot(sim.mod.8.ms.lm)
testTemporalAutocorrelation(sim.mod.8.ms.lm, time = (na.omit(data_dem_chamois_deer)$Year_N) # test temporal correlation
```

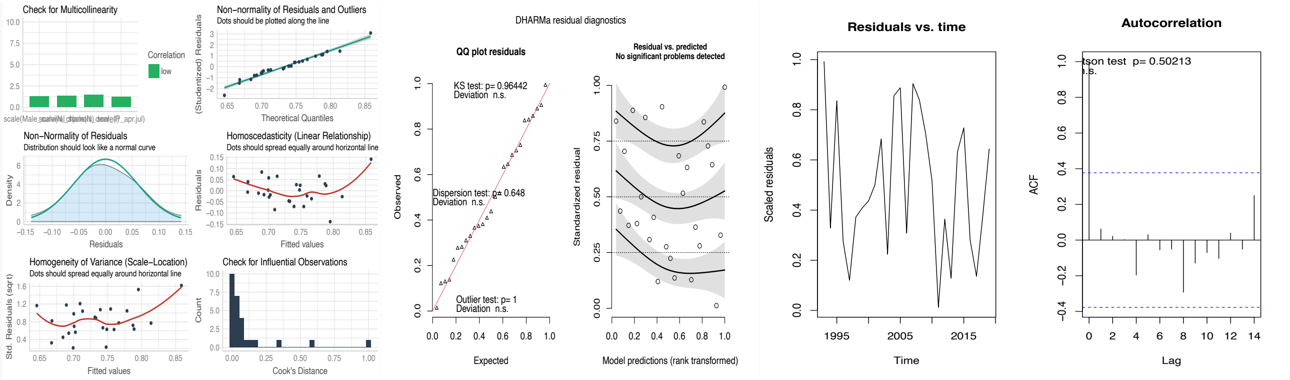


```
check_model(mod.7.ms.lm)
sim.mod.7.ms.lm <- simulateResiduals(mod.7.ms.lm)
plot(sim.mod.7.ms.lm)
testTemporalAutocorrelation(sim.mod.7.ms.lm, time = (na.omit(data_dem_chamois_deer)$Year_N) # test temporal correlation
```

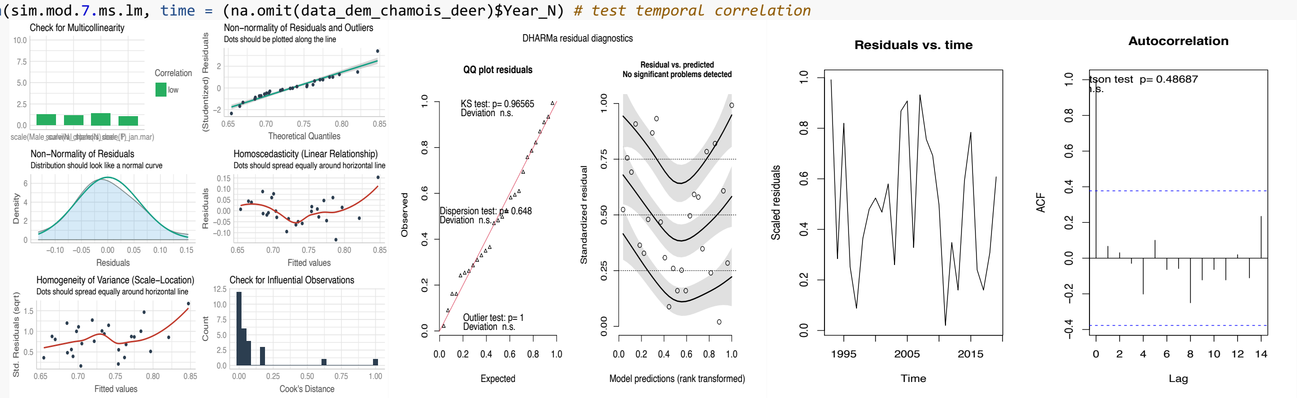


```
check_model(mod.5.ms.lm)
sim.mod.5.ms.lm <- simulateResiduals(mod.5.ms.lm)
plot(sim.mod.5.ms.lm)
testTemporalAutocorrelation(sim.mod.5.ms.lm, time = (na.omit(data_dem_chamois_deer)$Year_N) # test temporal correlation
```

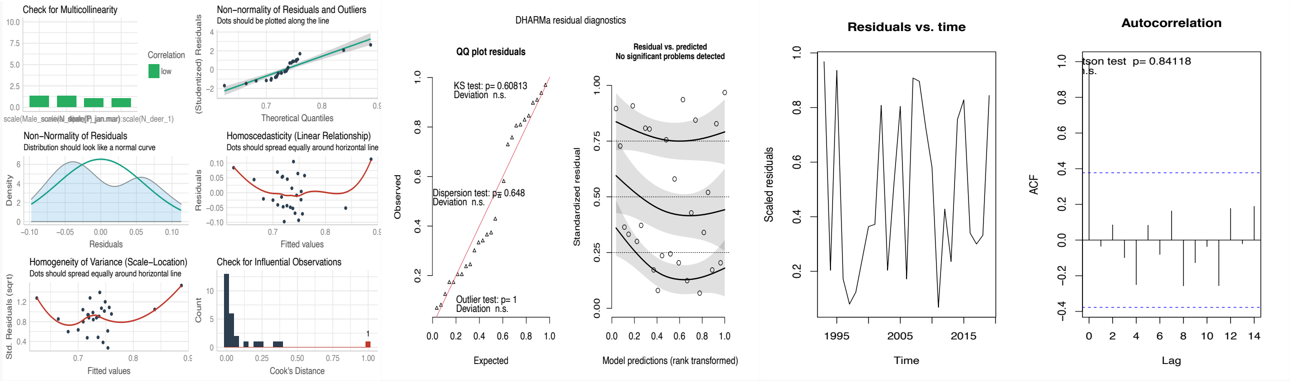


```
#==============================================================================
# 11) COMPARING BAYESIAN & OLS MODEL RESULTS
#==============================================================================

# birth rate

parameters(mod.4.br.stan, ci = 0.95, digits = 3, ci_digits=3)
Possible multicollinearity between scale(N_deer_1) and scale(Birth_rate_t_1) (r = 0.71). This might lead to inappropriate results. See 'Details' in '?rope'.
Parameter                          | Median |            95% CI |     pd | % in ROPE |  Rhat |      ESS |                 Prior
-----------------------------------------------------------------------------------------------------------------------------
(Intercept)                        |  0.561 | [ 0.547,  0.576] |   100% |        0% | 1.000 | 13905.47 | Normal (0.57 +- 0.17)
scale(Birth_rate_t_1)              |  0.013 | [-0.008,  0.035] | 89.19% |    24.52% | 1.001 |  7358.65 | Normal (0.00 +- 0.17)
scale(P1_apr.jul)                  |  0.024 | [ 0.006,  0.042] | 99.30% |     3.23% | 1.000 |  8132.51 | Normal (0.00 +- 0.17)
scale(N_deer_1)                    | -0.026 | [-0.049, -0.002] | 98.17% |     5.38% | 1.001 |  6742.93 | Normal (0.00 +- 0.17)
scale(N_chamois_1)                 | -0.021 | [-0.040, -0.002] | 98.32% |     6.92% | 1.001 |  6977.55 | Normal (0.00 +- 0.17)
scale(P1_apr.jul):scale(N_deer_1) | -0.021 | [-0.034, -0.008] | 99.84% |     1.60% | 1.001 |  8957.81 | Normal (0.00 +- 0.14)

parameters(mod.4.br.lm, ci = 0.95, digits = 3, ci_digits=3)
Parameter              | Coefficient |    SE |            95% CI |  t(22) |      p
---------------------------------------------------------------------------------
(Intercept)            |       0.561 | 0.007 | [ 0.547,  0.575] | 82.508 | < .001
Birth_rate_t_1         |       0.013 | 0.010 | [-0.008,  0.034] |  1.280 | 0.214
P1_apr.jul             |       0.024 | 0.009 | [ 0.006,  0.042] |  2.720 | 0.013
N_deer_1               |      -0.025 | 0.011 | [-0.049, -0.002] | -2.249 | 0.035
N_chamois_1            |      -0.021 | 0.009 | [-0.039, -0.002] | -2.262 | 0.034
P1_apr.jul * N_deer_1 |      -0.021 | 0.006 | [-0.034, -0.008] | -3.297 | 0.003

# kid survival

parameters(mod.17.ks.stan, ci=0.95, digits = 3, ci_digits=3)
Parameter              | Median |          95% CI |     pd | % in ROPE |  Rhat |      ESS |                 Prior
--------------------------------------------------------------------------------------------------------------
(Intercept)            |  0.383 | [ 0.369, 0.396] |   100% |        0% | 1.000 | 18145.21 | Normal (0.38 +- 0.10)
scale(Kid_survival_1) |  0.022 | [ 0.008, 0.036] | 99.79% |     0.74% | 1.000 | 13615.00 | Normal (0.00 +- 0.10)
scale(P_jan.mar)       | -0.009 | [-0.023, 0.005] | 90.26% |    20.78% | 1.000 | 13703.75 | Normal (0.00 +- 0.10)

parameters(mod.17.ks.lm, ci=0.95, digits = 3, ci_digits=3)
Parameter      | Coefficient |    SE |          95% CI |  t(24) |      p
--------------------------------------------------------------------------
(Intercept)    |       0.383 | 0.006 | [ 0.370, 0.396] | 59.977 | < .001
Kid_survival_1 |       0.022 | 0.007 | [ 0.008, 0.036] |  3.223 | 0.004
P_jan.mar      |      -0.009 | 0.007 | [-0.023, 0.005] | -1.320 | 0.199
```

```
# adult female  survival

parameters(mod.1.fs.stan, ci=0.95, digits = 3, ci_digits=3)
Parameter                         | Median |           95% CI |     pd | % in ROPE |  Rhat |      ESS |                 Prior
---------------------------------------------------------------------------------------------------------------------------
(Intercept)                       |  0.737 | [ 0.713,  0.760] |   100% |        0% | 1.000 | 17774.52 | Normal (0.75 +- 0.21)
scale(Female_survival_1)          | -0.023 | [-0.049,  0.003] | 95.92% |    12.11% | 1.000 | 13501.91 | Normal (0.00 +- 0.21)
scale(P_jan.mar)                  | -0.014 | [-0.038,  0.012] | 87.20% |    28.71% | 1.000 | 13137.74 | Normal (0.00 +- 0.21)
scale(N_chamois)                  | -0.010 | [-0.036,  0.016] | 77.26% |    38.32% | 1.000 | 12548.49 | Normal (0.00 +- 0.22)
scale(N_deer_1)                   | -0.050 | [-0.075, -0.023] | 99.94% |     0.18% | 1.000 | 13204.97 | Normal (0.00 +- 0.21)
scale(P_jan.mar):scale(N_chamois) | -0.040 | [-0.062, -0.017] | 99.96% |     0.48% | 1.000 | 13725.58 | Normal (0.00 +- 0.20)

parameters(mod.1.fs.lm, ci=0.95, digits = 3, ci_digits=3)
Parameter             | Coefficient |    SE |           95% CI |  t(21) |      p
--------------------------------------------------------------------------------
(Intercept)           |       0.737 | 0.011 | [ 0.714,  0.760] | 66.388 | < .001
Female_survival_1     |      -0.023 | 0.013 | [-0.049,  0.003] | -1.847 | 0.079
P_jan.mar             |      -0.014 | 0.012 | [-0.039,  0.011] | -1.165 | 0.257
N_chamois             |      -0.010 | 0.012 | [-0.035,  0.016] | -0.795 | 0.435
N_deer_1              |      -0.050 | 0.012 | [-0.076, -0.024] | -4.044 | < .001
P_jan.mar * N_chamois |      -0.040 | 0.011 | [-0.062, -0.017] | -3.701 | 0.001

# adult male  survival

parameters(mod.11.ms.stan, ci=0.95, digits = 3, ci_digits=3)
Parameter               | Median |           95% CI |     pd | % in ROPE |  Rhat |      ESS |                 Prior
-----------------------------------------------------------------------------------------------------------------
(Intercept)             |  0.736 | [ 0.710,  0.761] |   100% |        0% | 1.000 | 15475.16 | Normal (0.73 +- 0.19)
scale(Male_survival_1)  |  0.006 | [-0.024,  0.035] | 66.38% |    37.04% | 1.000 | 12123.30 | Normal (0.00 +- 0.19)
scale(N_deer_1)         | -0.045 | [-0.077, -0.015] | 99.67% |     0.83% | 1.001 | 10875.18 | Normal (0.00 +- 0.19)
scale(N_chamois)        | -0.029 | [-0.056,  0.000] | 97.97% |     5.38% | 1.001 | 12082.45 | Normal (0.00 +- 0.20)

parameters(mod.11.ms.lm, ci=0.95, digits = 3, ci_digits=3)
Parameter       | Coefficient |    SE |           95% CI |  t(23) |      p
--------------------------------------------------------------------------
(Intercept)     |       0.736 | 0.012 | [ 0.710,  0.761] | 59.378 | < .001
Male_survival_1 |       0.006 | 0.014 | [-0.023,  0.035] |  0.423 | 0.676
N_deer_1        |      -0.046 | 0.015 | [-0.076, -0.015] | -3.106 | 0.005
N_chamois       |      -0.029 | 0.013 | [-0.057, -0.002] | -2.199 | 0.038

#=============================================================================
# 12) OLS MODEL AVERAGING RESULTS
#=============================================================================

# birth rate

avg.model.br.lm <- model.avg(selection.br.lm, delta < 4) # nothing to average!

parameters(avg.model.br.lm, ci=0.95, digits = 3, ci_digits=3)
Parameter             | Coefficient |    SE |           95% CI |      z |      p
--------------------------------------------------------------------------------
(Intercept)           |       0.561 | 0.007 | [ 0.546,  0.575] | 76.251 | < .001
Birth_rate_t_1        |       0.016 | 0.012 | [-0.007,  0.040] |  1.363 | 0.173
P1_apr.jul            |       0.026 | 0.010 | [ 0.006,  0.046] |  2.603 | 0.009
N_deer_1              |      -0.022 | 0.013 | [-0.048,  0.004] |  1.633 | 0.103
N_chamois_1           |      -0.021 | 0.010 | [-0.039, -0.002] |  2.137 | 0.033
N_deer_1 * P1_apr.jul |      -0.022 | 0.007 | [-0.035, -0.008] |  3.129 | 0.002

r2(mod.4.br.lm)
# R2 for Linear Regression

       R2: 0.785
  adj. R2: 0.737
r2(mod.6.br.lm)
# R2 for Linear Regression

       R2: 0.736
  adj. R2: 0.690
```

```
# kid survival

avg.model.ks.lm <- model.avg(selection.ks.lm, delta < 4)
parameters(avg.model.ks.lm, ci=0.95, digits = 3, ci_digits=3)
Parameter             | Coefficient |    SE |           95% CI |      z |      p
--------------------------------------------------------------------------------
(Intercept)           |       0.382 | 0.007 | [ 0.369,  0.396] | 55.257 | < .001
Kid_survival_1        |       0.022 | 0.008 | [ 0.007,  0.037] |  2.874 | 0.004
P_jan.mar             |      -0.011 | 0.008 | [-0.026,  0.004] |  1.394 | 0.163
N_chamois             |   9.248e-04 | 0.007 | [-0.013,  0.015] |  0.129 | 0.897
N_chamois * P_jan.mar |      -0.014 | 0.006 | [-0.026, -0.001] |  2.140 | 0.032
N_deer_1              |      -0.001 | 0.008 | [-0.018,  0.015] |  0.137 | 0.891
P_apr.jul             |      -0.002 | 0.007 | [-0.017,  0.012] |  0.317 | 0.751

r2(mod.17.ks.lm)
# R2 for Linear Regression

       R2: 0.412
  adj. R2: 0.363

r2(mod.13.ks.lm)
# R2 for Linear Regression

       R2: 0.523
  adj. R2: 0.436

r2(mod.12.ks.lm)
# R2 for Linear Regression

       R2: 0.376
  adj. R2: 0.324

r2(mod.18.ks.lm)
# R2 for Linear Regression

       R2: 0.372
  adj. R2: 0.320

r2(mod.19.ks.lm)
# R2 for Linear Regression

       R2: 0.371
  adj. R2: 0.319

r2(mod.9.ks.lm)
# R2 for Linear Regression

       R2: 0.412
  adj. R2: 0.336

r2(mod.15.ks.lm)
# R2 for Linear Regression

       R2: 0.412
  adj. R2: 0.335

r2(mod.1.ks.lm)
# R2 for Linear Regression

       R2: 0.534
  adj. R2: 0.423
# adult female survival

avg.model.fs.lm <- model.avg(selection.fs.lm, delta < 4) # nothing to select!
parameters(avg.model.fs.lm, ci=0.95, digits = 3, ci_digits=3)
Parameter             | Coefficient |    SE |           95% CI |  t(21) |      p
--------------------------------------------------------------------------------
(Intercept)           |       0.737 | 0.011 | [ 0.714,  0.760] | 66.388 | < .001
Female_survival_1     |      -0.023 | 0.013 | [-0.049,  0.003] | -1.847 | 0.079
P_jan.mar             |      -0.014 | 0.012 | [-0.039,  0.011] | -1.165 | 0.257
```

```
N_chamois             |      -0.010 | 0.012 | [-0.035,  0.016] | -0.795 | 0.435
N_deer_1              |      -0.050 | 0.012 | [-0.076, -0.024] | -4.044 | < .001
P_jan.mar * N_chamois |      -0.040 | 0.011 | [-0.062, -0.017] | -3.701 | 0.001

r2(mod.1.fs.lm)
# R2 for Linear Regression

       R2: 0.655
  adj. R2: 0.573

# adult male survival

avg.model.ms.lm <- model.avg(selection.ms.lm, delta < 4)
parameters(avg.model.ms.lm, ci=0.95, digits = 3, ci_digits=3)
Parameter            | Coefficient |    SE |           95% CI |      z |      p
--------------------------------------------------------------------------------
(Intercept)          |       0.736 | 0.013 | [ 0.709,  0.762] | 54.549 | < .001
Male_survival_1      |       0.007 | 0.015 | [-0.024,  0.037] |  0.427 | 0.669
N_deer_1             |      -0.043 | 0.016 | [-0.075, -0.011] |  2.652 | 0.008
N_chamois            |      -0.030 | 0.015 | [-0.059, -0.002] |  2.087 | 0.037
P_apr.jul            |      -0.010 | 0.014 | [-0.038,  0.019] |  0.678 | 0.498
P_jan.mar            |      -0.003 | 0.015 | [-0.032,  0.026] |  0.232 | 0.817
N_deer_1 * P_jan.mar |      -0.047 | 0.025 | [-0.096,  0.001] |  1.920 | 0.055

r2(mod.11.ms.lm)
# R2 for Linear Regression

       R2: 0.395
  adj. R2: 0.317

r2(mod.12.ms.lm)
# R2 for Linear Regression

       R2: 0.268
  adj. R2: 0.207

r2(mod.8.ms.lm)
# R2 for Linear Regression

       R2: 0.409
  adj. R2: 0.302

r2(mod.7.ms.lm)
# R2 for Linear Regression

       R2: 0.403
  adj. R2: 0.295

r2(mod.5.ms.lm)
# R2 for Linear Regression

       R2: 0.384
  adj. R2: 0.272


#==============================================================================
# 13) PLOT MARGINAL EFFECTS SIGNIFICANT PREDICTORS FROM OLS MODELS
#==============================================================================

# birth rate

# significant effects to plot:
# 1) P1_apr.jul
# 2) N_deer_1
# 3) N_deer_1 : scale(P1_apr.jul)
# 4) N_chamois_1

quantile(data_dem_chamois_deer$N_deer_1, probs = c(0.1, 0.5, 0.9)) #  check the 10%, 50% and 90% quantiles of red deer abundance
```

```r
par(mfrow = c(2,2))

visreg(mod.4.br.lm, xvar="P1_apr.jul",
       rug=FALSE,
       ylim = c(0.5,1.0),
       overlay = TRUE,
       xlab="Spring-summer precipitation [t-1] (in mm)",
       ylab="Chamois birth rate [t]",
       fill=list(col=grey(c(0.7), alpha=0.4)),
       line=list(lty=1:3, col = "black", lwd = 1.5),
       points=list(cex=1, pch=16, col = "black"), # partial residuals
       partial = FALSE,
       cex.lab = 1.25)
with(data_dem_chamois_deer, points(P1_apr.jul, Birth_rate_t, pch=16, cex=1, col = "black")) # real data
text(500, 0.975, "A", cex = 1, font = 2)

visreg(mod.4.br.lm, xvar="N_deer_1",
       rug=FALSE,
       ylim = c(0.5,1.0),
       overlay = TRUE,
       xlab="Red deer abundance [t-1]",
       ylab="Chamois birth rate [t]",
       fill=list(col=grey(c(0.7), alpha=0.4)),
       line=list(lty=1:3, col = "black", lwd = 1.5),
       points=list(cex=1, pch=16, col = "black"), # partial residuals
       partial = FALSE,
       cex.lab = 1.25)
with(data_dem_chamois_deer, points(N_deer_1, Birth_rate_t, pch=16, cex=1, col = "black")) # real data
text(1850, 0.975, "B", cex = 1, font = 2)

visreg(mod.4.br.lm , xvar = "P1_apr.jul",  by = "N_deer_1",
       rug = FALSE,
       ylim = c(0.5,1.0),
       overlay = TRUE,
       strip.names = NULL,
       breaks = c(773.8, 1281, 1674.2), # 10%, 50% and 90% quantiles of red deer abundance
       xlab = "Spring-summer precipitation [t-1] (in mm)",
       ylab = "Chamois birth rate [t]",
       fill = list(col = grey(c(0.7), alpha = 0.4)),
       line = list(lty = 1:3, col = "black", lwd = 1.5),
       points = list(cex = 1, pch = 16, col = gray((27:1)/27)[as.factor(data_dem_chamois_deer$N_deer_1)]), # partial residuals
       partial = FALSE,
       cex.lab = 1.25)
legend("topleft", c("10th perc. deer abundance [t-1]", "50th perc. deer abundance [t-1]", "90th perc. deer abundance [t-1]"),
       lty = 1:3, col = "black", lwd = 1.5, cex = 0.8, bty = "n")
with(data_dem_chamois_deer, points(P1_apr.jul, Birth_rate_t, pch= 16, cex = 1, col = gray((27:1)/40)[as.factor(data_dem_chamois_deer$N_deer_1)])) # real data
text(500, 0.975, "C", cex = 1, font = 2)

visreg(mod.4.br.lm, xvar="N_chamois_1",
       rug=FALSE,
       ylim = c(0.5,1.0),
       overlay = TRUE,
       xlab="Chamois abundance [t-1]",
       ylab="Chamois birth rate [t]",
       fill=list(col=grey(c(0.7), alpha=0.4)),
       line=list(lty=1:3, col = "black", lwd = 1.5),
       points=list(cex=1, pch=16, col = "black"), # partial residuals
       partial = FALSE,
       cex.lab = 1.25)
with(data_dem_chamois_deer, points(N_chamois_1, Birth_rate_t, pch=16, cex=1, col = "black")) # real data
text(2125, 0.975, "D", cex = 1, font = 2)
```

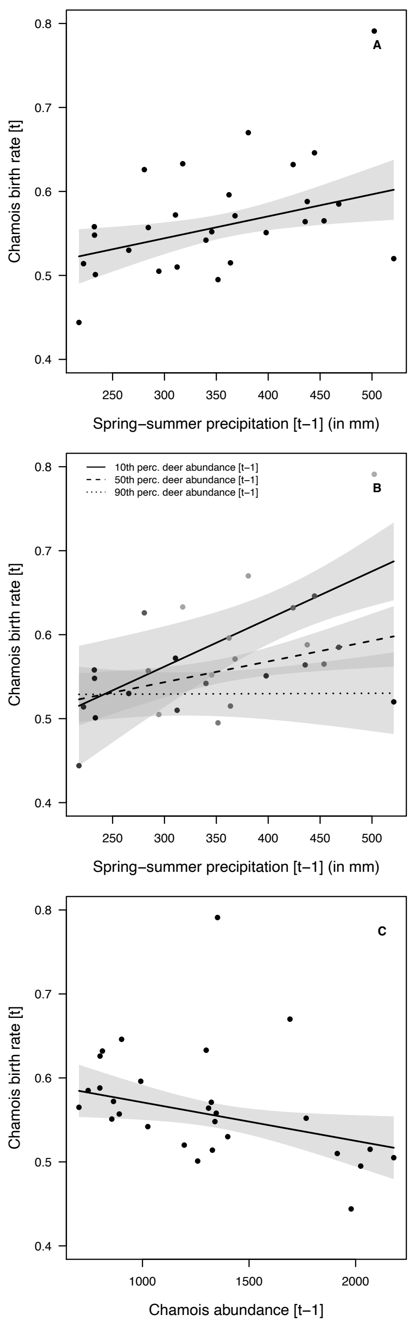
A
Chamois birth rate [t]
Spring−summer precipitation [t−1] (in mm)
0.4
0.5
0.6
0.7
0.8
250
300
350
400
450
500
B
10th perc. deer abundance [t−1]
50th perc. deer abundance [t−1]
90th perc. deer abundance [t−1]
Chamois birth rate [t]
Spring−summer precipitation [t−1] (in mm)
0.4
0.5
0.6
0.7
0.8
250
300
350
400
450
500
C
Chamois birth rate [t]
Chamois abundance [t−1]
0.4
0.5
0.6
0.7
0.8
1000
1500
2000

```
# kid survival
# significant effects to plot:
# 1) N_chamois : scale(P_jan.mar)

quantile(data_dem_chamois_deer$N_chamois, probs = c(0.1, 0.5, 0.9)) # just check the 10%, 50% and 90% quantiles of chamois

par(mfrow = c(1,1))
visreg(mod.13.ks.lm, xvar = "P_jan.mar",  by = "N_chamois",
       rug = FALSE,
       ylim = c(0.1,0.5),
       overlay = TRUE,
       strip.names = NULL,
       breaks = c(799.2, 1304, 1997.8), # 10%, 50% and 90% quantiles of chamois abundance
       xlab = "Winter precipitation [t+1] (in mm)",
       ylab = "Chamois kid survival [(t+1)/t]",
       fill = list(col  =grey(c(0.7), alpha = 0.4)),
       line = list(lty=1:3, col = "black", lwd = 1.5),
       points = list(cex = 1, pch = 16, col = gray((27:1)/30)[as.factor(data_dem_chamois_deer$N_chamois)]), # partial residuals
       partial =FALSE,
       cex.lab = 1.25)
legend("bottomleft", c("10th perc. chamois abundance [t]", "50th perc. chamois abundance [t]", "90th perc. chamois abundance [t]"),
       lty = 1:3, col = "black", lwd = 1.5, cex = 0.8, bty = "n")
with(data_dem_chamois_deer, points(P_jan.mar, Kid_survival, pch=16, cex=1, col=gray((27:1)/40)[as.factor(data_dem_chamois_deer$N_chamois)])) # real data
```

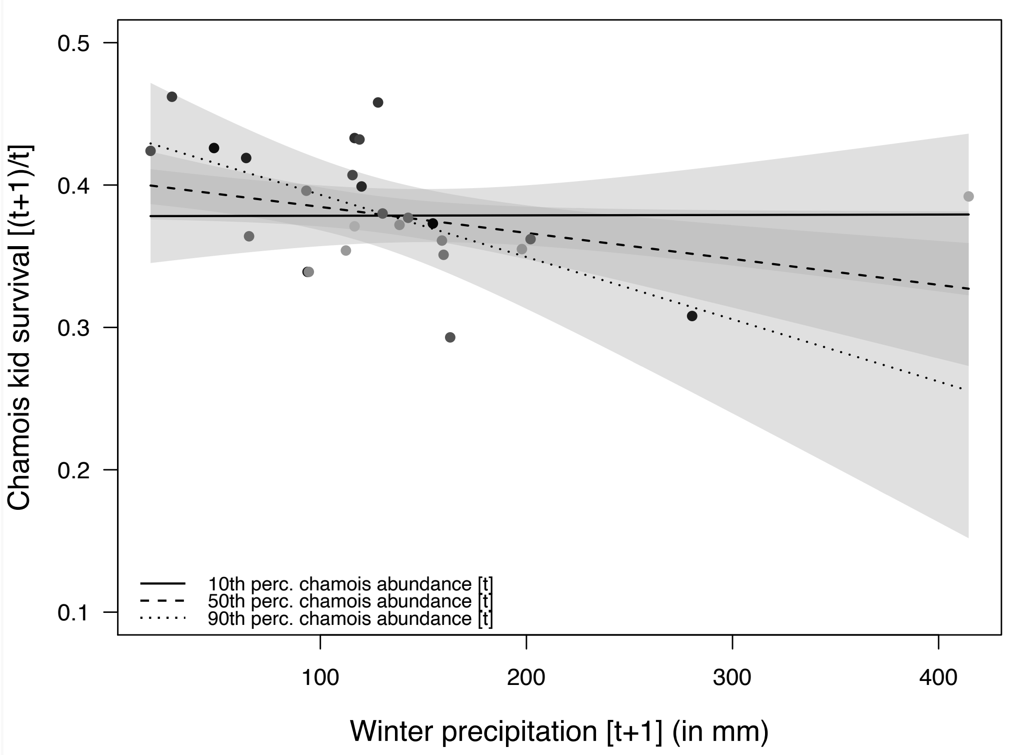

```
# adult female  survival

# significant effects to plot:
# 1) N_chamois : scale(P_jan.mar)
# 2) N_deer_1

quantile(data_dem_chamois_deer$N_chamois, probs = c(0.1, 0.5, 0.9)) # just check the 10%, 50% and 90% quantiles of chamois

par(mfrow = c(2,1))
visreg(mod.1.fs.lm, xvar = "P_jan.mar",  by = "N_chamois",
       rug = FALSE,
       ylim = c(0.0,1.0),
       overlay = TRUE,
       strip.names = NULL,
       breaks = c(799, 1284, 1987), # 10%, 50% and 90% quantiles of chamois abundance
       xlab = "Winter precipitation [t+1] (in mm)",
       ylab = "Chamois female survival [(t+1)/t]",
       fill = list(col  =grey(c(0.7), alpha = 0.4)),
       line = list(lty=1:3, col = "black", lwd = 1.5),
       points = list(cex = 1, pch = 16, col = gray((27:1)/30)[as.factor(data_dem_chamois_deer$N_chamois)]), # partial residuals
       partial =FALSE,
       cex.lab = 1.25)
legend("bottomleft", c("10th perc. chamois abundance [t]", "50th perc. chamois abundance [t]", "90th perc. chamois abundance [t]"),
       lty = 1:3, col = "black", lwd = 1.5, cex = 0.8, bty = "n")
with(data_dem_chamois_deer, points(P_jan.mar, Female_survival, pch=16, cex=1, col=gray((27:1)/40)[as.factor(data_dem_chamois_deer$N
_deer_1)])) # real data
text(400, 0.975, "A", cex = 1, font = 2)

visreg(mod.1.fs.lm, xvar = "N_deer_1",
       rug = FALSE,
       ylim = c(0.5,1),
       overlay = TRUE,
       xlab = "Red deer abundance [t-1]",
       ylab = "Chamois female survival [(t+1)/t]",
       fill = list(col = grey(c(0.7), alpha = 0.4)),
       line = list(lty = 1:3, col = "black", lwd = 1.5),
       points = list(cex = 1, pch = 16, col = "black"), # partial residuals
       partial = FALSE,
       cex.lab = 1.25)
with(data_dem_chamois_deer, points(N_deer_1, Female_survival, pch=16, cex=1, col="black")) # real data
text(1875, 0.975, "B", cex = 1, font = 2)
```

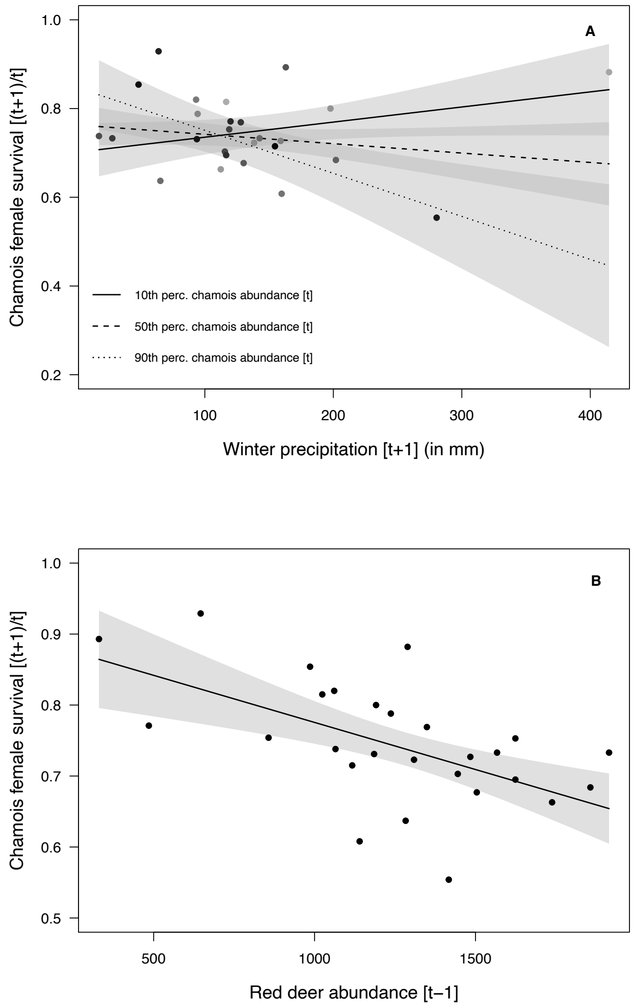

```r
# adult male  survival

# significant effects to plot:
# 1) N_chamois
# 2) N_deer_1

par(mfrow = c(3,1))

visreg(mod.11.ms.lm, xvar = "N_chamois",
       rug = FALSE,
       ylim = c(0.5,1),
       overlay = TRUE,
       strip.names = NULL,
       xlab = "Chamois abundance [t]",
       ylab = "Chamois male survival [(t+1)/t]",
       fill = list(col = grey(c(0.7), alpha = 0.4)),
       line = list(lty = 1:3, col = "black", lwd = 1.5),
       points = list(cex = 1, pch = 16, col = "black"), # partial residuals
       partial = FALSE,
       cex.lab = 1.25)
with(data_dem_chamois_deer, points(N_chamois, Male_survival, pch = 16, cex = 1, col = "black")) # real data
text(2175, 0.975, "A", cex = 1, font = 2)

visreg(mod.11.ms.lm, xvar = "N_deer_1",
       rug = FALSE,
       ylim = c(0.5,1),
       overlay = TRUE,
       strip.names = NULL,
       xlab = "Red deer abundance [t-1]",
       ylab = "Chamois male survival [(t+1)/t]",
       fill = list(col = grey(c(0.7), alpha = 0.4)),
       line = list(lty = 1:3, col = "black", lwd = 1.5),
       points = list(cex = 1, pch = 16, col = "black"), # partial residuals
       partial = FALSE,
       cex.lab = 1.25)
with(data_dem_chamois_deer, points(N_deer_1, Male_survival, pch = 16, cex = 1, col = "black")) # real data
text(1875, 0.975, "B", cex = 1, font = 2)

# there is a datapoint that might influence the slope of the regression lines
# we refit the model using a robust approach
mod.11.ms.rlm <- lmrob(formula(mod.11.ms.lm), data = data_dem_chamois_deer, method="SMDM")
parameters(mod.11.ms.lm, ci=0.95, digits = 3, ci_digits=3)
parameters(mod.11.ms.rlm, ci=0.95, digits = 3, ci_digits=3) # the datapoint doesn't have an influence on the the red deer effect, but on the chamois

visreg(mod.11.ms.rlm, xvar = "N_deer_1",
       rug = FALSE,
       ylim = c(0.5,1),
       overlay = TRUE,
       strip.names = NULL,
       xlab = "Red deer abundance [t-1]",
       ylab = "Chamois male survival [(t+1)/t]",
       fill = list(col = grey(c(0.7), alpha = 0.4)),
       line = list(lty = 1:3, col = "black", lwd = 1.5),
       points = list(cex = 1, pch = 16, col = "black"), # partial residuals
       partial = FALSE,
       cex.lab = 1.25)
with(data_dem_chamois_deer, points(N_deer_1, Male_survival, pch = 16, cex = 1, col = "black")) # real data
text(1875, 0.975, "C", cex = 1, font = 2)
text(1100, 0.975, "Robust estimation", cex = 1)
```

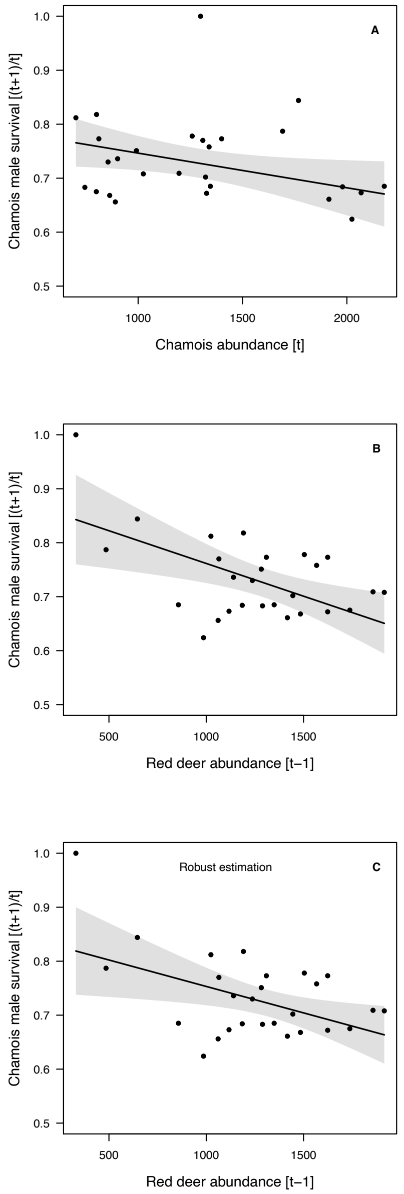

```r
#==============================================================================
# 14) PATH ANALYSIS
#==============================================================================

# HYPOTHESIS H5:

# Red deer density at [t-1] has a negative effect on chamois growth rate, mediated by:
# a) birth rate
# b) kid survival
# c) adult female survival
# d) adult male survival

# add standardized red deer, as in regression analyses

data_dem_chamois_deer$N_deer_1.st <- scale(data_dem_chamois_deer$N_deer_1)

# define path models

model.a.lav <- '# regression between deer density at [t-1] and chamois birth rate at [t]
                Birth_rate_t ~ a * N_deer_1.st
                Y_chamois ~ b * Birth_rate_t

                # indirect effect of deer density at [t-1] on chamois growth rate between [t] and [t+1] mediated by birth rate
                indirect := a * b'

model.b.lav <- '# regression between deer density at [t-1] and chamois kid survival between [t] and [t+1]
                Kid_survival ~ a * N_deer_1.st
                Y_chamois ~ b * Kid_survival

                # indirect effect of deer density at [t-1] on chamois growth rate between [t] and [t+1] mediated by kid survival
                indirect := a * b'

model.c.lav <- '# regression between deer density at [t-1] and chamois adult female survival between [t] and [t+1]
                Female_survival ~ a * N_deer_1.st
                Y_chamois ~ b * Female_survival

                # indirect effect of deer density at [t-1] on chamois growth rate between [t] and [t+1] mediated by female survival
                indirect := a * b'

model.d.lav <- '# regression between deer density at [t-1] and chamois adult male survival between [t] and [t+1]
                Male_survival ~ a * N_deer_1.st
                Y_chamois ~ b * Male_survival

                # indirect effect of deer density at [t-1] on chamois growth rate between [t] and [t+1] mediated by male survival
                indirect := a * b'

# fit path models

fit.model.a.lav <- sem(model.a.lav, data = data_dem_chamois_deer)
fit.model.b.lav <- sem(model.b.lav, data = data_dem_chamois_deer)
fit.model.c.lav <- sem(model.c.lav, data = data_dem_chamois_deer)
fit.model.d.lav <- sem(model.d.lav, data = data_dem_chamois_deer)

# return fit measures

fitMeasures(fit.model.a.lav, c("chisq", "df", "pvalue", "rmsea", "rmsea.pvalue", "aic"))
       chisq           df       pvalue        rmsea rmsea.pvalue          aic
       0.529        1.000        0.467        0.000        0.482     -140.364
fitMeasures(fit.model.b.lav, c("chisq", "df", "pvalue", "rmsea", "rmsea.pvalue", "aic"))
       chisq           df       pvalue        rmsea rmsea.pvalue          aic
      13.027        1.000        0.000        0.667        0.000     -128.776
fitMeasures(fit.model.c.lav, c("chisq", "df", "pvalue", "rmsea", "rmsea.pvalue", "aic"))
       chisq           df       pvalue        rmsea rmsea.pvalue          aic
       1.010        1.000        0.315        0.020        0.331     -142.231
fitMeasures(fit.model.d.lav, c("chisq", "df", "pvalue", "rmsea", "rmsea.pvalue", "aic"))
       chisq           df       pvalue        rmsea rmsea.pvalue          aic
       4.082        1.000        0.043        0.338        0.050     -114.510
```

```
# show standardized coefficients for the best models
standardizedSolution(fit.model.a.lav)
            lhs op          rhs est.std    se      z pvalue ci.lower ci.upper
1 Birth_rate_t  ~  N_deer_1.st  -0.637 0.102 -6.242  0.000   -0.837   -0.437
2    Y_chamois  ~ Birth_rate_t   0.791 0.070 11.265  0.000    0.653    0.928
3 Birth_rate_t ~~ Birth_rate_t   0.594 0.130  4.568  0.000    0.339    0.849
4    Y_chamois ~~    Y_chamois   0.375 0.111  3.373  0.001    0.157    0.592
5  N_deer_1.st ~~  N_deer_1.st   1.000 0.000     NA     NA    1.000    1.000
6     indirect :=          a*b  -0.504 0.099 -5.100  0.000   -0.697   -0.310

standardizedSolution(fit.model.c.lav)
               lhs op             rhs est.std    se      z pvalue ci.lower ci.upper
1 Female_survival  ~     N_deer_1.st  -0.556 0.122 -4.552  0.000   -0.796   -0.317
2       Y_chamois  ~ Female_survival   0.898 0.036 24.615  0.000    0.827    0.970
3 Female_survival ~~ Female_survival   0.691 0.136  5.080  0.000    0.424    0.957
4       Y_chamois ~~       Y_chamois   0.193 0.066  2.950  0.003    0.065    0.322
5     N_deer_1.st ~~     N_deer_1.st   1.000 0.000     NA     NA    1.000    1.000
6        indirect :=             a*b  -0.500 0.115 -4.330  0.000   -0.726   -0.273


################################################################################
# END OF ANALYSIS
################################################################################
```
